# Supplementary material for: Novel lipid nanoparticle in a mRNA cancer vaccine drives tumor control via type I IFNs and effector CD8+ T cells
Source: Mol Ther Nucleic Acids. 2026 Aug 27;37(4):103076. doi: 10.1016/j.omtn.2026.103076 (PMC13594926; doi:10.1016/j.omtn.2026.103076)
Supplement: Supplementary file 1 [file mmc1.pdf]

OMTN, Volume *37*

Supplemental information

Novel lipid nanoparticle in a mRNA cancer vaccine drives tumor control via type I IFNs and effector CD8$^+$ T cells

Matthew J. Gold, Douglas G. Millar, Jun Liu, Haritha Menon, Yury Karpov, Kirstin Olsen, Liliam Teixeira Oliveira, Charlotte Dubé, Charles W. Tran, Alisha R. Elford, Jordan A. Schwartz, Rajesh Krishnan Gopalakrishna Panicker, Eric G. Marcusson, Pamela S. Ohashi, and Natalia Martin Orozco

Table S1 – Summary of physiochemical characteristics of mRNA-LNP formulations (mean±SEM)

**Formulation**	**INTENT-1.1**	**INTENT-2.1**	**ALC-0315**	**SM-102**
Size (nm)	79 ± 12	71±4	140	80±2
PDI	0.23±0.09	0.09±0.02	0.09	0.06±0.01
mRNA encapsulation (%)	91±3	96±0.3	79	94±0.8
Zeta Potential (mV)	5±0.6	4±3	-10.3	-1±7
mRNA integrity (%)	96±1	97±1	99	96±2

Table S2 – Flow Cytometry Antibodies

**Antibody**	**Vendor**	**Clone**
Fixable viability dye e506	Thermo Fisher	n/a
CD16/32	BD Pharmingen	2.4G2
CD4	Thermo Fisher	GK1.5
CD8a	Thermo Fisher	53-6.7
CD11b	Thermo Fisher	M1/70
CD11c	Thermo Fisher	N418
CD45	Thermo Fisher	30-F11
CD80	Thermo Fisher	16-10A1
CD137/4-1BB	Thermo Fisher	17B5
CD279/PD-1	Thermo Fisher	J43
TCRβ	BD Pharmingen	H57-597
MHC-II	Thermo Fisher	AF6-120.1
SiglecH	Thermo Fisher	eBio440c
Ly6C	Thermo Fisher	HK1.4
Ly6G	Thermo Fisher	1A8
iNOS	Thermo Fisher	CXNFT
IFNγ	Thermo Fisher	XMG1.2

TNFα	Thermo Fisher	MPX-XT22

Table S3 – qRT-PCR Primers

Gene	Forward	Reverse
Actb	GGCTGTATTCCCCTCCATCG	CCAGTTGGTAACAATGCCATGT
Ccl2	CTCGGACTGTGATGCCTTAAT	TGGATCCACACCTTGCATTTA
Ccl3	TTCTCTGTACCATGACACTCTGC	CGTGGAATCTTCCGGCTGTAG
Cxcl1	GCTGGGATTCACCTCAAGAA	TGGCTATGACTTCGGTTTGG
Cxcl2	AGTGAACTGCGCTGTCAATGC	AGGCAAACTTTTTGACCGCC
Cxcl9	GGAGTTCGAGGAACCCTAGTG	GGGATTTGTAGTGGATCGTGC
Cxcl10	CCAAGTGCTGCCGTCATTTTC	GGCTCGCAGGGATGATTTCAA
Il1a	GCTTGAGTCGGCAAAGAAATC	GAGAGATGGTCAATGGCAGAA
Il1b	AAGGAGAACCAAGCAACGACAAAA	TGGGGAACTCTGCAGACTCAAACT
Il1rn	TTGTGCCAAGTCTGGAGATG	CTCAGAGCGGATGAAGGTAAAG
Il6	TAG TCC TTC CTA CCC CAA TTT CC	TTG GTC CTT AGC CAC TCC TTC
Ifna-pan	CCTGAGAAAGAAGAAACACAGCC	GGCTCTCCAGACTTCTGCTCTG
	CCTGAGAGAGAAGAAACACAGCC	GGCTCTCCAGATTTCTGCTCTG
Ifnb1	GCACTGGGTGGAATGAGACT	AGTGGAGAGCAGTTGAGGACA
Ifng	CTCTTCCTCATGGCTGTTTCT	TTCTTCCACATCTATGCCACTT
Mx1	CCAACTGGAATCCTCCTGGAA	GCCGCACCTTCTCCTCATAG

Table S4 – Outcomes for Repeat-Dose Phase of the INTENT-2.11 Tolerability Study in Rats

Parameter	Time of Assessment	Outcomes	
		Dosing Period	Recovery Period
**Mortality**	Daily	No mortality observed in any animal group.	No mortality observed in<br>any animal group.
**Clinical observations**	Prior and daily for 3 days post each administration, otherwise twice weekly until the end of dosing/recovery period.	No systemic clinical signs were observed.	No systemic clinical signs were observed.

**Parameter**	**Time of Assessment**	**Outcomes**	
		**Dosing Period**	**Recovery Period**
**Injection Site Observations (Draize Score)**	Daily for 3 days post each administration, otherwise weekly until the end of dosing/recovery period.	Dose-proportionate edema (slight to moderate) associated with abnormal gait (up to 2 days post-dose) occurred after each administration, with a higher severity in females and fully resolving between doses. No erythema, except for few female animals (3/10) at 200 µg dose.	No longer present by the end of the recovery period.
**Body Weight**	Prior and daily for 3 days post each administration, otherwise twice weekly until the end of dosing/recovery period.	Body weight gain over time with a transient decrease (1-2 days) following each administration (males only).	Similar body weight gain between all groups.
**Food Consumption**	Weekly until the end of dosing/recovery period.	Decrease of food intake during the first week following each administration (males and females).	Similar food intake between all groups.
**Hematology** [(1)]	End of dosing /recovery period.	Marked increases in white blood cell counts (neutrophils and monocytes) observed at all doses (males and females).<br>Slight reduction in reticulocyte counts in males (all doses) and females (100 and 200 µg doses).	Persistent increases in white blood cell counts at higher doses with a lower magnitude indicating an ongoing recovery.
**Clinical Chemistry** [(2)]	End of dosing /recovery period.	Slight decrease in cholesterol levels (male only) at higher doses.	Return to normal at the end of the recovery.
**Gross Examination** [(3)]	End of dosing /recovery period.	Enlargement of the popliteal lymph node (1/10 male, 100 µg), dark area at the injection site (1/10 female, 200 µg).	No abnormal findings.

Parameter	Time of Assessment	Outcomes	
		Dosing Period	Recovery Period
**Organ Weight** [(4)]	End of dosing /recovery period.	No changes.	No changes.
**Histopathology** [(5)]	End of dosing /recovery period.<br><br>*(Performed on control and 200 µg dose group animals; (n=2/sex))*	Injection site: Minimal to marked inflammation of the perimysium and/or the endomysium in all animals (2/2 males and 2/2 females) dosed at 200 µg.<br>Lymph nodes: Increased incidence of mild increased cellularity of the inguinal lymph node noted in animals dosed at 200 µg (1/2 male and 1/2 female) when compared to controls (1/2 male).<br>Liver: Minimal to mild periportal vacuolation of the hepatocytes was noted in all animals (2/2 males and 2/2 females) dosed at 200 µg. The same observation was also noted in a single control female animal at a lower severity (minimal).	Microscopic findings in the liver had resolved by the end of the recovery period. Inflammation at the injection site and vacuolated macrophages in the lymph nodes were still present with a decreased severity, suggesting an ongoing recovery.

(1) Hematology: Red blood cell, hematocrit, hemoglobin, white blood cell, mean corpuscular hemoglobin, morphology of cells, platelet, reticulocyte

(2) Clinical chemistry: A/G ratio, alanine aminotransferase, albumin, alkaline phosphatase, aspartate aminotransferase, bilirubin, calcium, chloride, cholesterol, gamma-glutamyl transferase, creatinine, creatine kinase, globulin, glucose, phosphorus, potassium, sodium, total protein, triglycerides, urea

(3) Gross examination: Adrenals, aorta, brain, cecum, colon, epididymis, esophagus, eyes, femur and marrow, heart, injection sites, kidneys, liver, lungs, axillary/inguinal/mandibular/mesenteric/popliteal lymph nodes, mammary glands, optic nerves, ovaries, pancreas, pituitary, prostate, rectum, salivary glands, sciatic nerve, seminal vesicles, skeletal muscle, skin and subcutis, duodenum, jejunum, ileum, spinal cord (cervical), spleen, sternum and marrow, stomach, testes, thymus, thyroid gland, tongue, trachea, urinary bladder, uterus, vagina

(4) Organ weight: Adrenals, brain, heart, kidneys, liver, lungs, ovaries, pituitary, prostate, spleen, testes, thymus, thyroid gland and parathyroids, uterus

(5) Histopathology: Liver, spleen, biceps femoris, inguinal lymph nodes

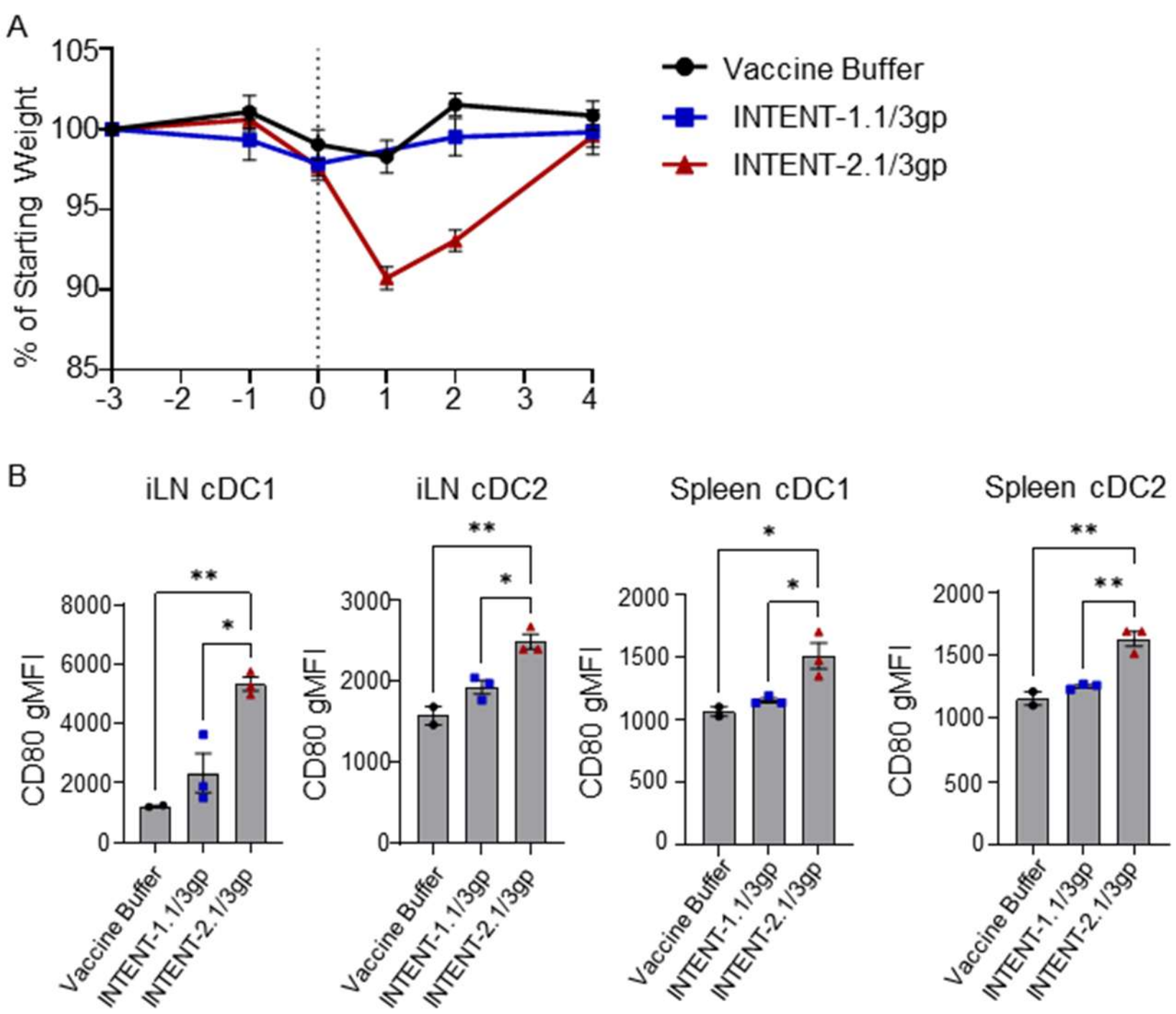

<u>Figure S1 – INTENT-1.1 and INTENT-2.1 Toxicity and APC Activation</u>

(A) Total body mass was measured prior to and following vaccine buffer or LNP administration (day 0) and shown as a percentage of starting weight (n=6-12). (B) Draining inguinal lymph nodes (iLN) and spleens were collected from C57BL/6 mice 24hrs after vaccine buffer or INTENT LNP administration (n=2-3). Dendritic cells were identified as CD11c$^+$MHC-II$^+$CD3$^-$CD19$^-$ and defined as cDC1 or cDC2 based on CD8a and CD11b expression, respectively. Statistics in (B) were performed by one-way ANOVA with Tukey's multiple comparison test, *$p<0.05$, **$p<0.01$.

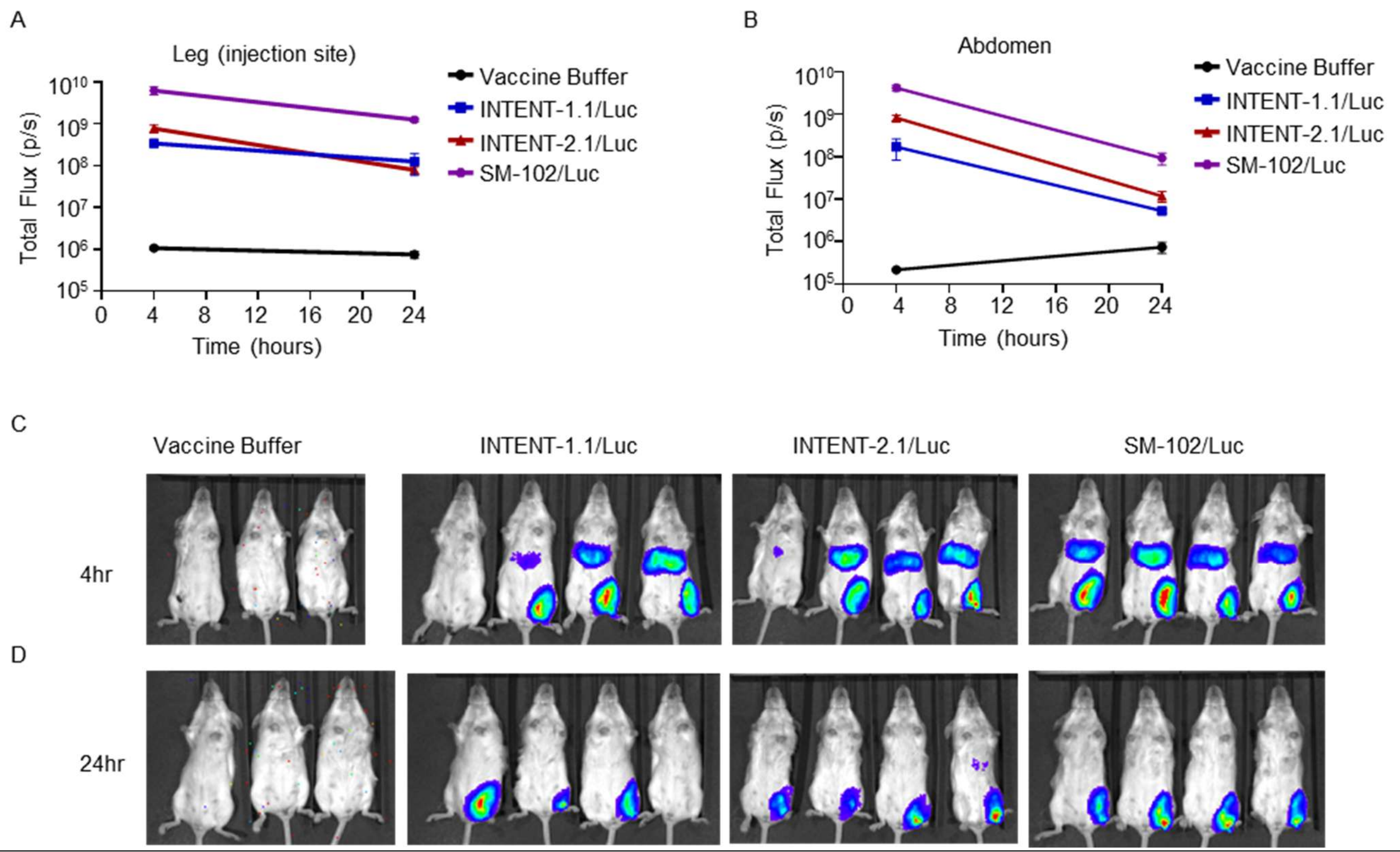

Figure S2 – *In vivo* biodistribution

BALBc mice were administered the indicated LNPs and 4hr or 24hr post-injection luciferin substrate was delivered prior to imaging. (A) Luminescence in the injection site area or (B) abdomen was quantified. Representative images (C) 4hrs and (D) 24hrs after LNP administration.

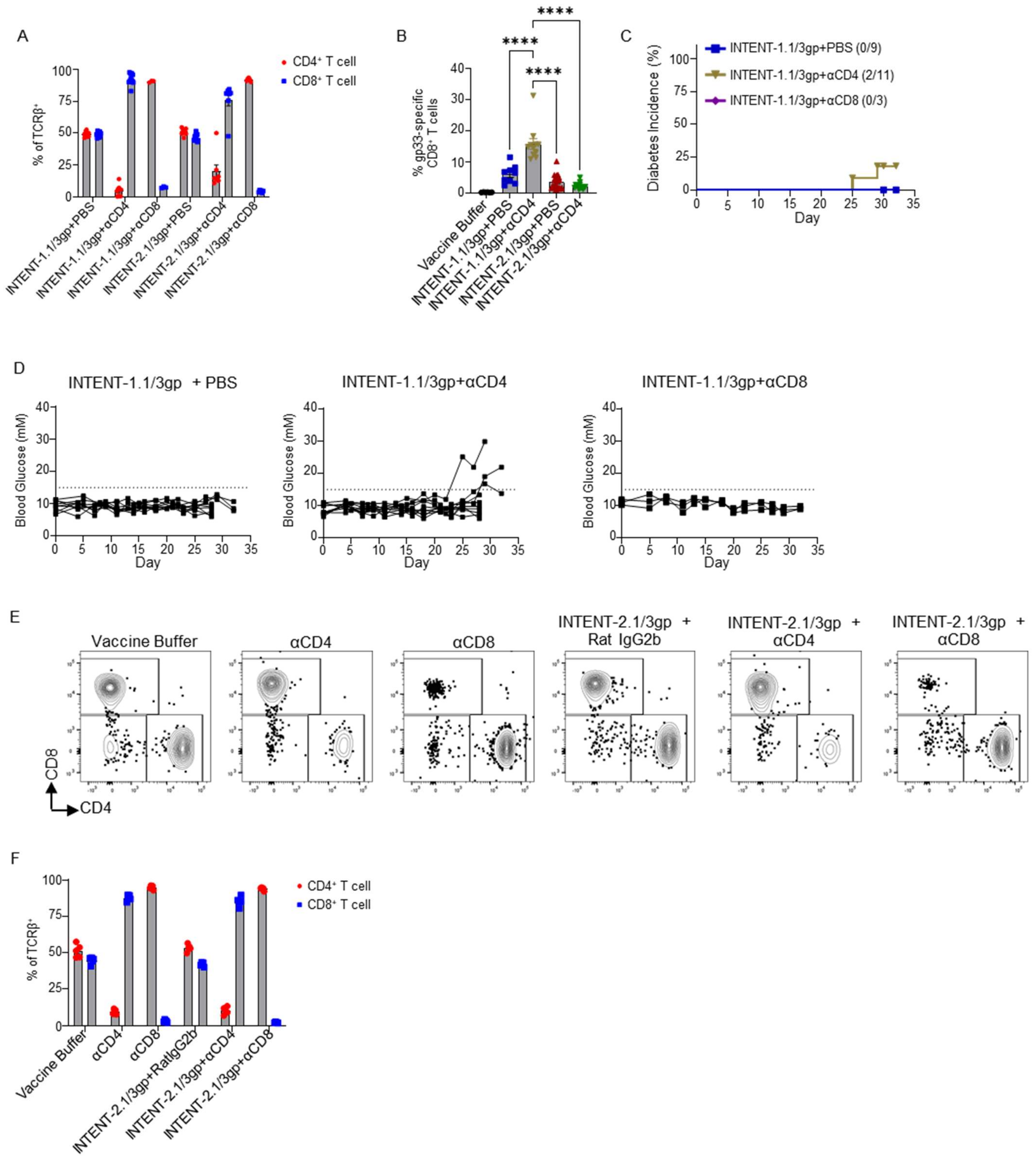

Figure S3 – CD4⁺ and CD8⁺ T cell depletion in RIPgp and MC38gp models

(A) Peripheral blood was collected on day 6-8 from RIPgp animals treated as in Figure 3A and depletion of $CD4^+$ and $CD8^+$ T cells was confirmed by flow cytometry, gated on $TCR\beta^+CD45^+$ cells . (B) Frequency of gp33-specific $CD8^+$ T cells in the blood from RIPgp animals treated with the indicated LNPs. (C) Diabetes incidence and (D) individual animal blood glucose from RIPgp mice. (E) Peripheral blood was collected on day 9 from MC38gp tumor bearing animals as treated in Figure 4E-G and shown gated on $TCR\beta^+CD45^+$ cells from the indicated treatment groups and summarized in (F). Statistics in (B) was performed by one-way ANOVA and Tukey's multiple comparisons test, ****$p<0.0001$.

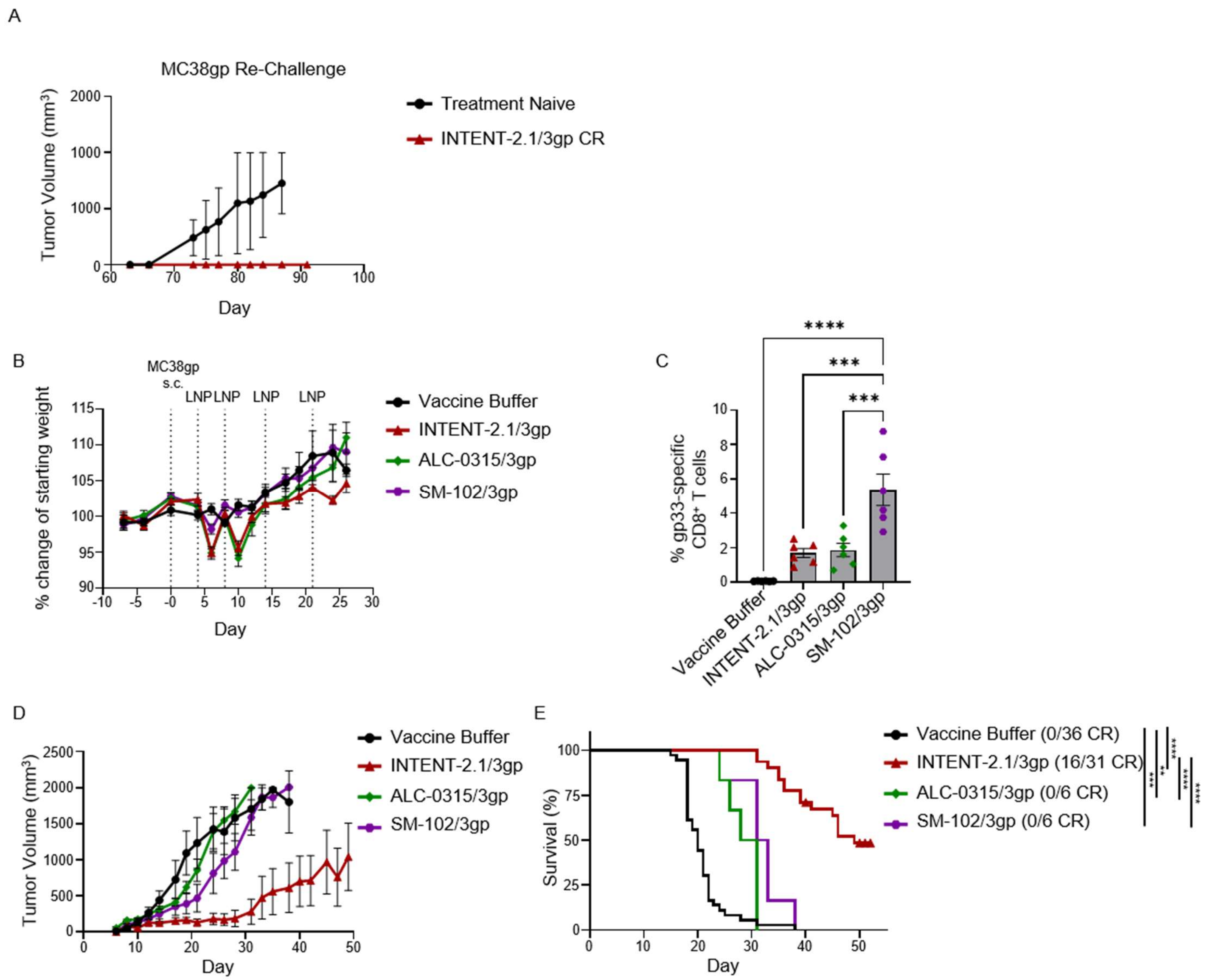

<u>Figure S4 – INTENT-2.1/3gp provides immune memory to tumor re-challenge and superior tumor control to benchmark LNPs</u>

(A) INTENT-2.1/3gp treated C57BL/6 mice that successfully cleared established MC38gp tumor (n=3) or naïve controls (n=2) were re-challenged with MC38gp tumor cells (1x10^6 cells s.c.) and tumor growth was monitored. (B) Percent change in body weight from C57BL/6 mice administered MC38gp cells (day 0) and the indicated LNPs (days 4, 8, 14 & 21). (C) Frequency of gp33-specific $CD8^+$ T cells in the blood on day 11, shown as frequency of total $CD8^+$ T cells. (D) Tumor volume and (E) survival of MC38gp tumor bearing animals treated with the indicated LNP. Statistics in (C) was performed by one-way ANOVA and Tukey's multiple comparison test and in (E) performed by log-rank test, **p<0.01, ***p<0.001 and ****p<0.0001.

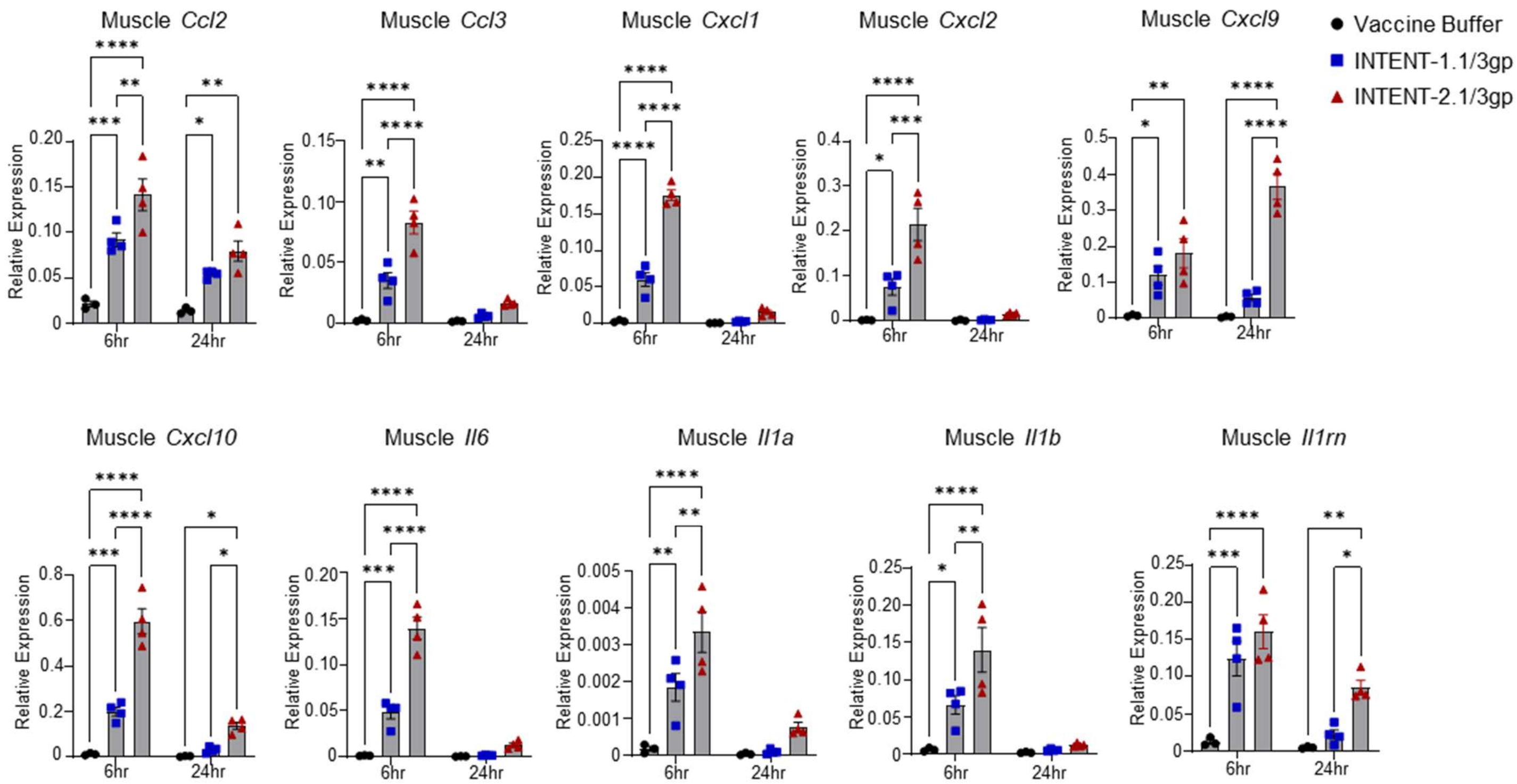

<u>Figure S5 – Adjuvant activity of INTENT LNPs in muscle tissue</u>

Expression data of the indicated genes in muscle tissue collected from the muscle tissue (injection site) following either vaccine formulation buffer, INTENT-1.1/3gp or INTENT-2.1/3gp immunization (n=3-4/group). Expression data is shown relative to *Actb*. Statistics was performed by two-way ANOVA followed by Tukey's multiple comparison test, *$p<0.05$, **$p<0.01$, ***$p<0.001$ and ****$p<0.0001$.

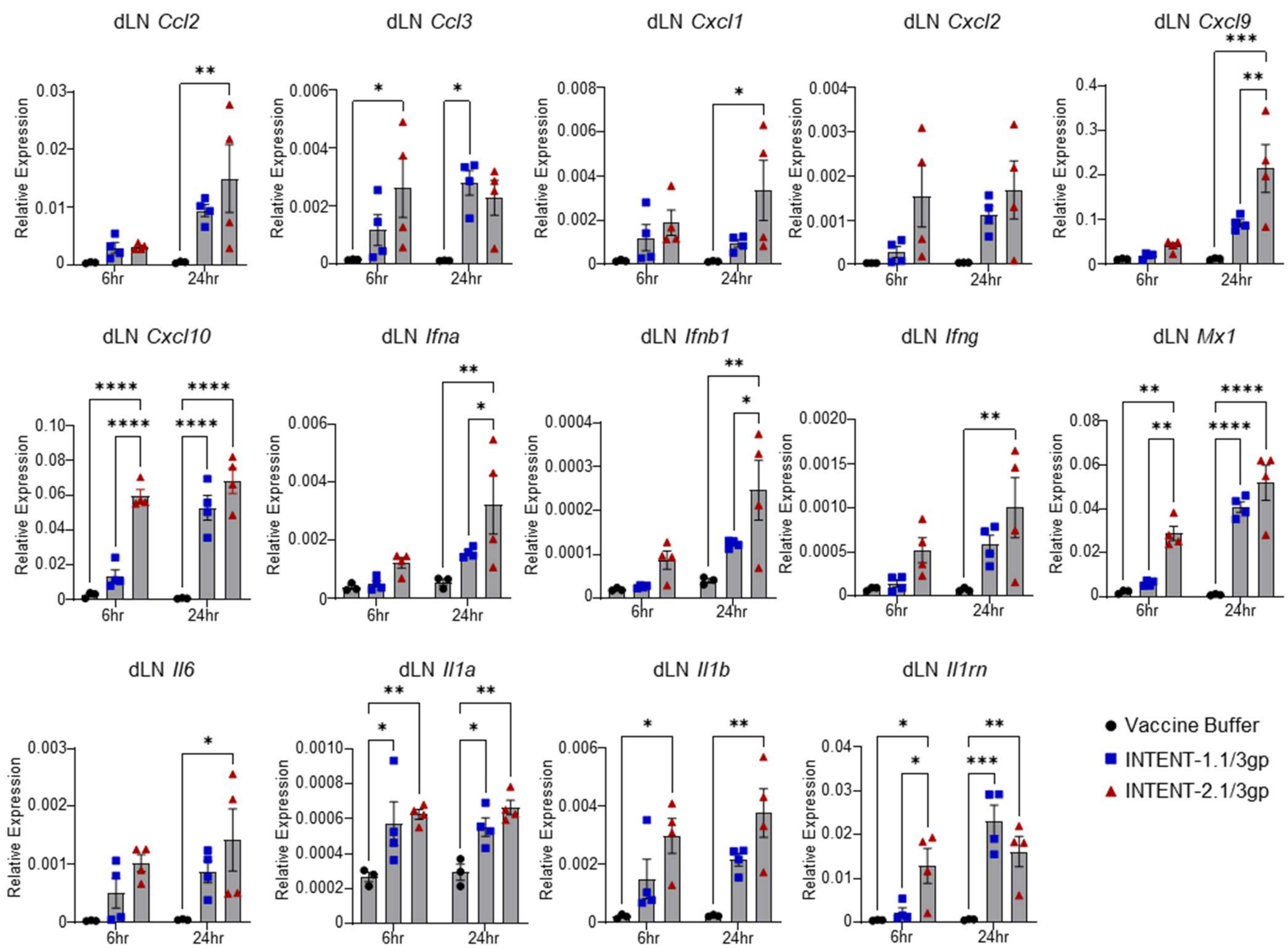

Figure S6 – Adjuvant activity of INTENT LNPs in the dLN

Expression data of the indicated genes in the draining inguinal lymph node (dLN) following either vaccine formulation buffer, INTENT-1.1/3gp or INTENT-2.1/3gp immunization (n=3-4/group). Expression data is shown relative to *Actb*. Statistics was performed by two-way ANOVA followed by Tukey's multiple comparison test, *p<0.05, **p<0.01, ***p<0.001 and ****p<0.0001.

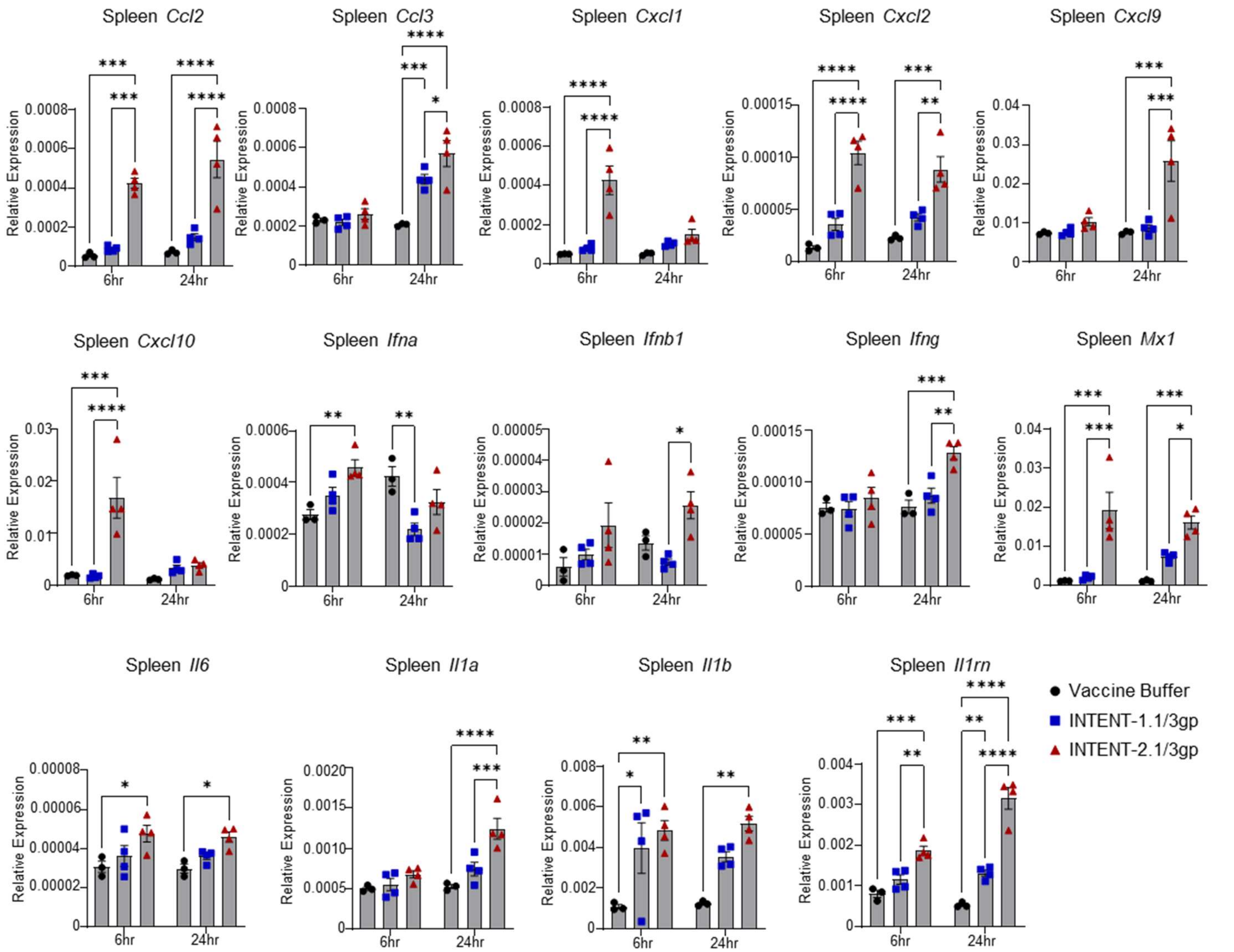

<u>Figure S7 – Adjuvant activity of INTENT LNPs in the spleen</u>

Expression data of the indicated genes in the spleen following either vaccine formulation buffer, INTENT-1.1/3gp or INTENT-2.1/3gp immunization (n=3-4/group). Expression data is shown relative to *Actb*. Statistics was performed by two-way ANOVA followed by Tukey's multiple comparison test, *p<0.05, **p<0.01, ***p<0.001 and ****p<0.0001.

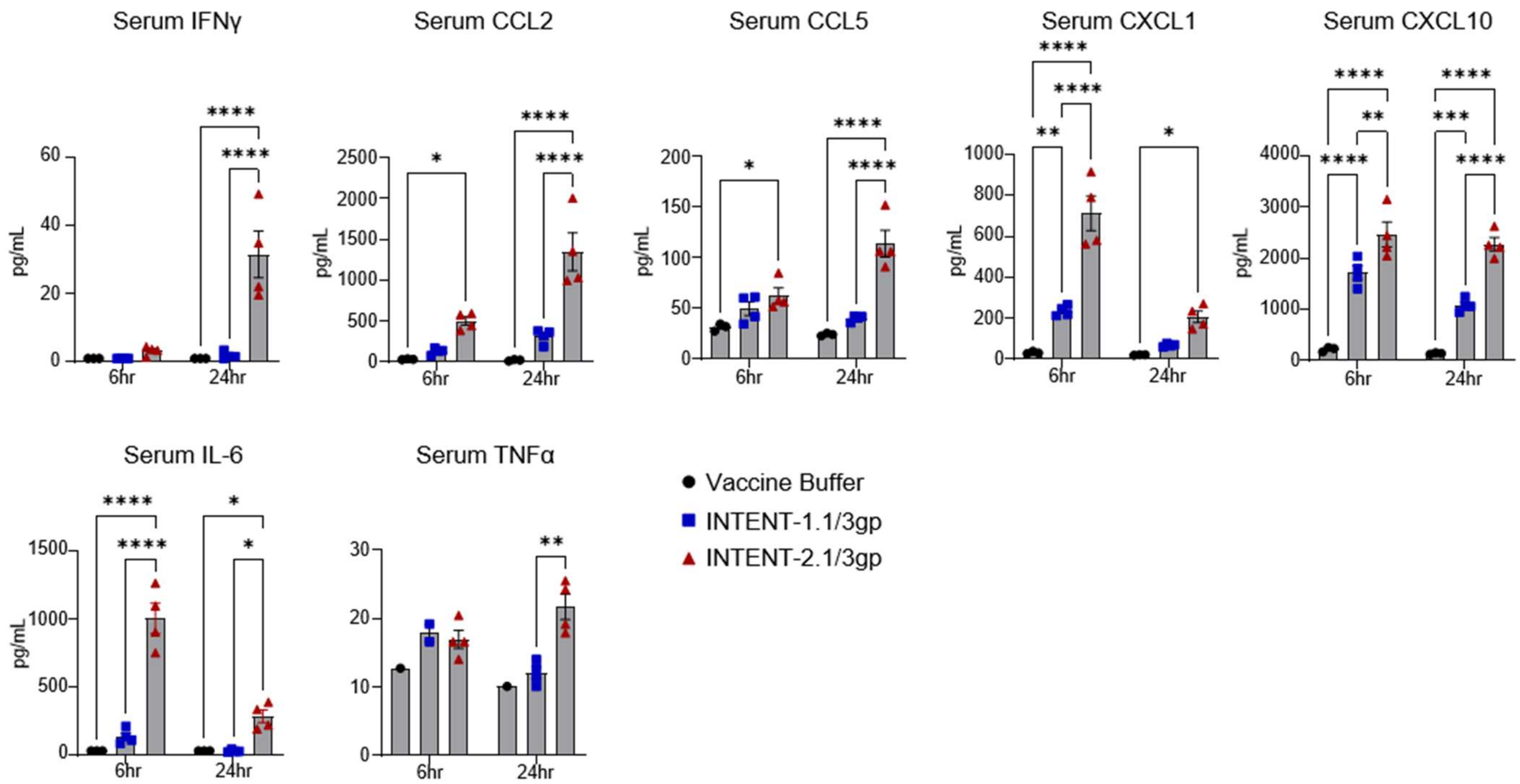

Figure S8 – Serum cytokine levels following INTENT LNP injection

Serum protein expression of the indicated cytokines/chemokines following either vaccine formulation buffer, INTENT-1.1/3gp or INTENT-2.1/3gp immunization (n=3-4/group). Statistics was performed by two-way ANOVA followed by Tukey's multiple comparison test, *p<0.05, **p<0.01, ***p<0.001 and ****p<0.0001.

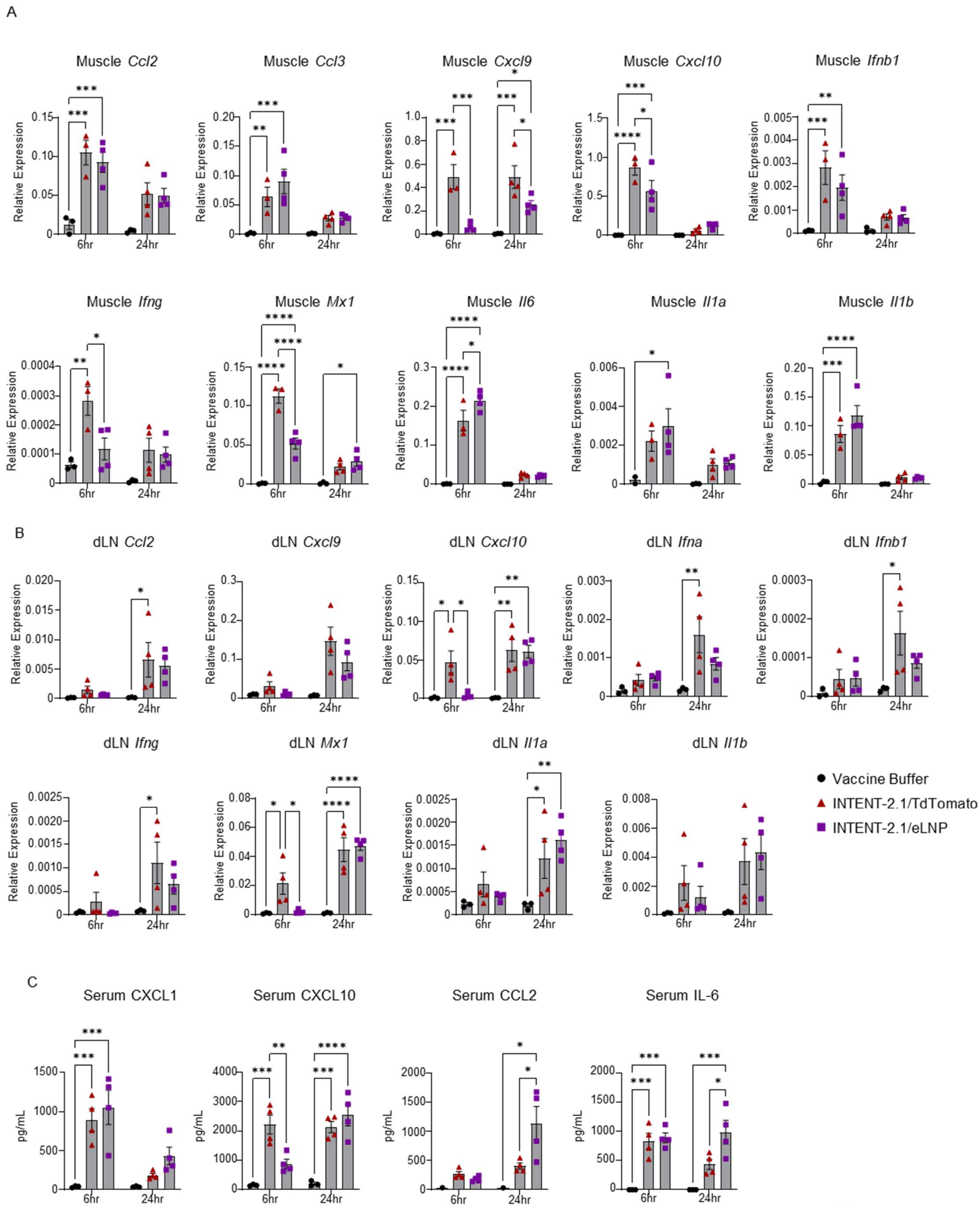
A
Muscle Ccl2
Muscle Ccl3
Muscle Cxcl9
Muscle Cxcl10
Muscle Ifnb1
Muscle Ifng
Muscle Mx1
Muscle Il6
Muscle Il1a
Muscle Il1b
B
dLN Ccl2
dLN Cxcl9
dLN Cxcl10
dLN Ifna
dLN Ifnb1
dLN Ifng
dLN Mx1
dLN Il1a
dLN Il1b
Vaccine Buffer
INTENT-2.1/TdTomato
INTENT-2.1/eLNP
C
Serum CXCL1
Serum CXCL10
Serum CCL2
Serum IL-6
Relative Expression
pg/mL
6hr
24hr

<u>Figure S9 – INTENT-2.1/eLNP immunogenicity</u>

Expression data of the indicated genes in the (A) muscle and (B) dLN and (C) protein levels in the serum following either vaccine formulation buffer, INTENT-2.1/3gp or INTENT-2.1/eLNP immunization (n=3-4/group). Expression data is shown relative to *Actb*. Statistics was performed by two-way ANOVA followed by Tukey's multiple comparison test, *p<0.05, **p<0.01, ***p<0.001 and ****p<0.0001.

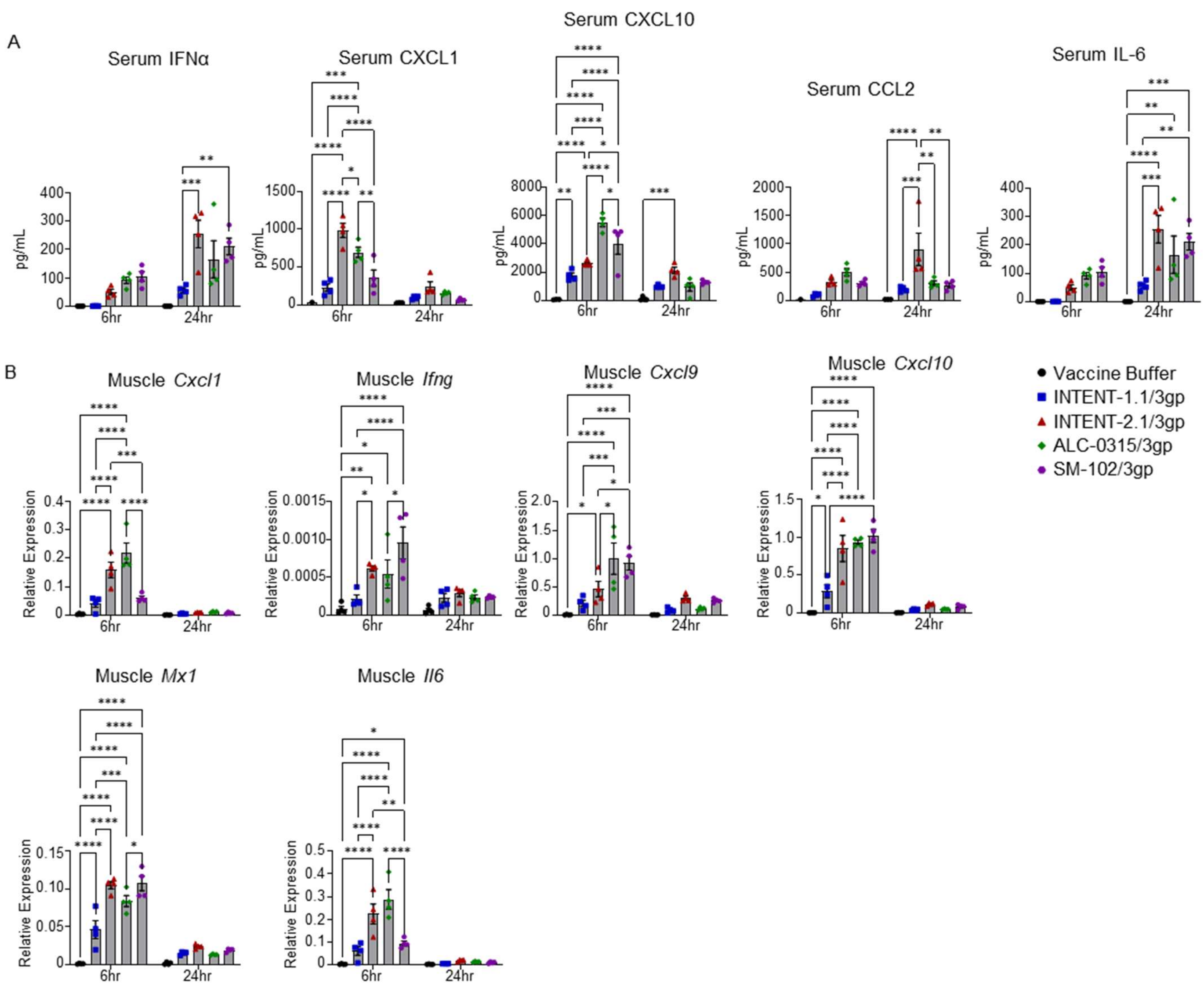

Figure S10 – Induction of cytokines and chemokines by INTENT, ALC-0315 and SM-102 LNPs in serum and muscle

C57BL/6 mice were administered vaccine buffer or the indicated LNP and (A) serum or (B) muscle tissue was collected 6 and 24 hours post injection for cytokine quantification (n=3-4). Statistics was performed by two-way ANOVA followed by Tukey's multiple comparison test, *p<0.05, **p<0.01, ***p<0.001 and ****p<0.0001.

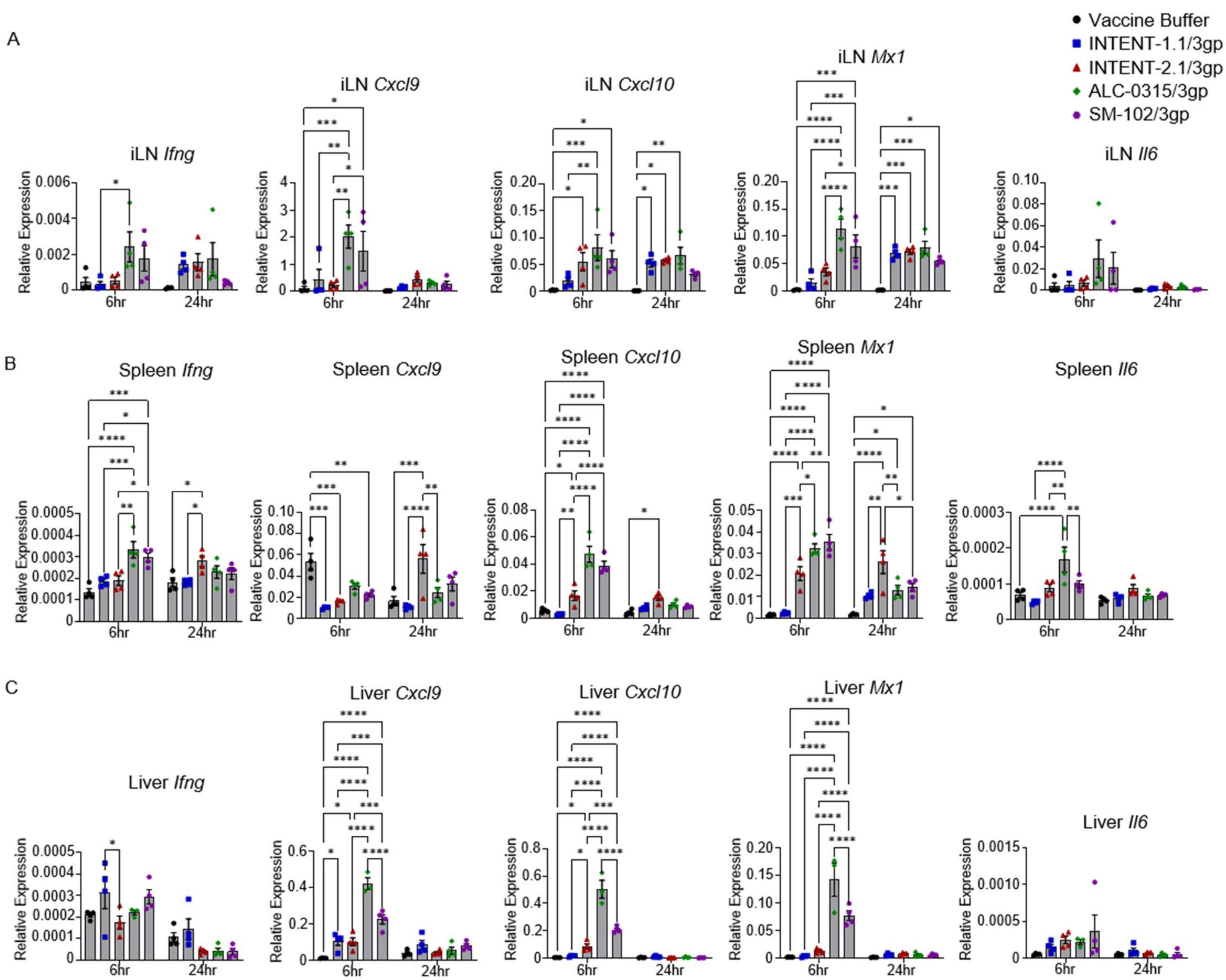

Figure S11 – Induction of cytokines and chemokines by INTENT, ALC-0315 and SM-102 LNPs in iLN, spleen and liver

C57BL/6 mice were administered vaccine buffer or the indicated LNP and (A) iLN, (B) spleen and (C) liver tissue was collected 6 and 24 hours post injection for cytokine quantification (n=3-4). Statistics was performed by two-way ANOVA followed by Tukey's multiple comparison test, *p<0.05, **p<0.01, ***p<0.001 and ****p<0.0001.

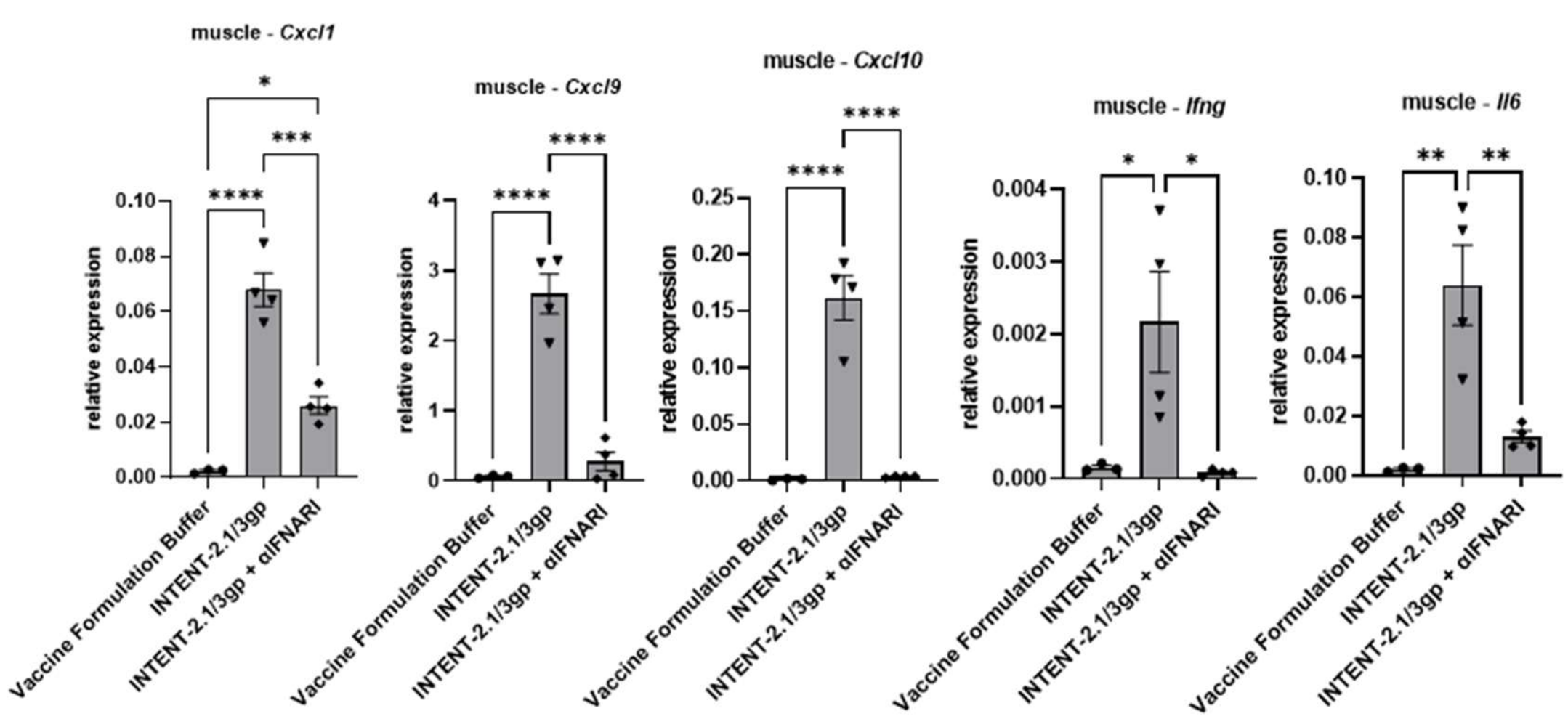

Figure S12 – INTENT-2.1/3gp immunogenicity is regulated by type I IFNs

C57BL/6 mice were administered PBS (200µl/i.p.) or αIFNARI (500µg/i.p.) prior to vaccine formulation buffer or INTENT-2.1/3gp administration (12.5µg/50µl i.m.) and expression of the indicated genes was quantified by qRT-PCR and shown relative to *Actb*. Statistics was performed by one-way ANOVA followed by Tukey's multiple comparison test, *p<0.05, **p<0.01, ***p<0.001 and ****p<0.0001.

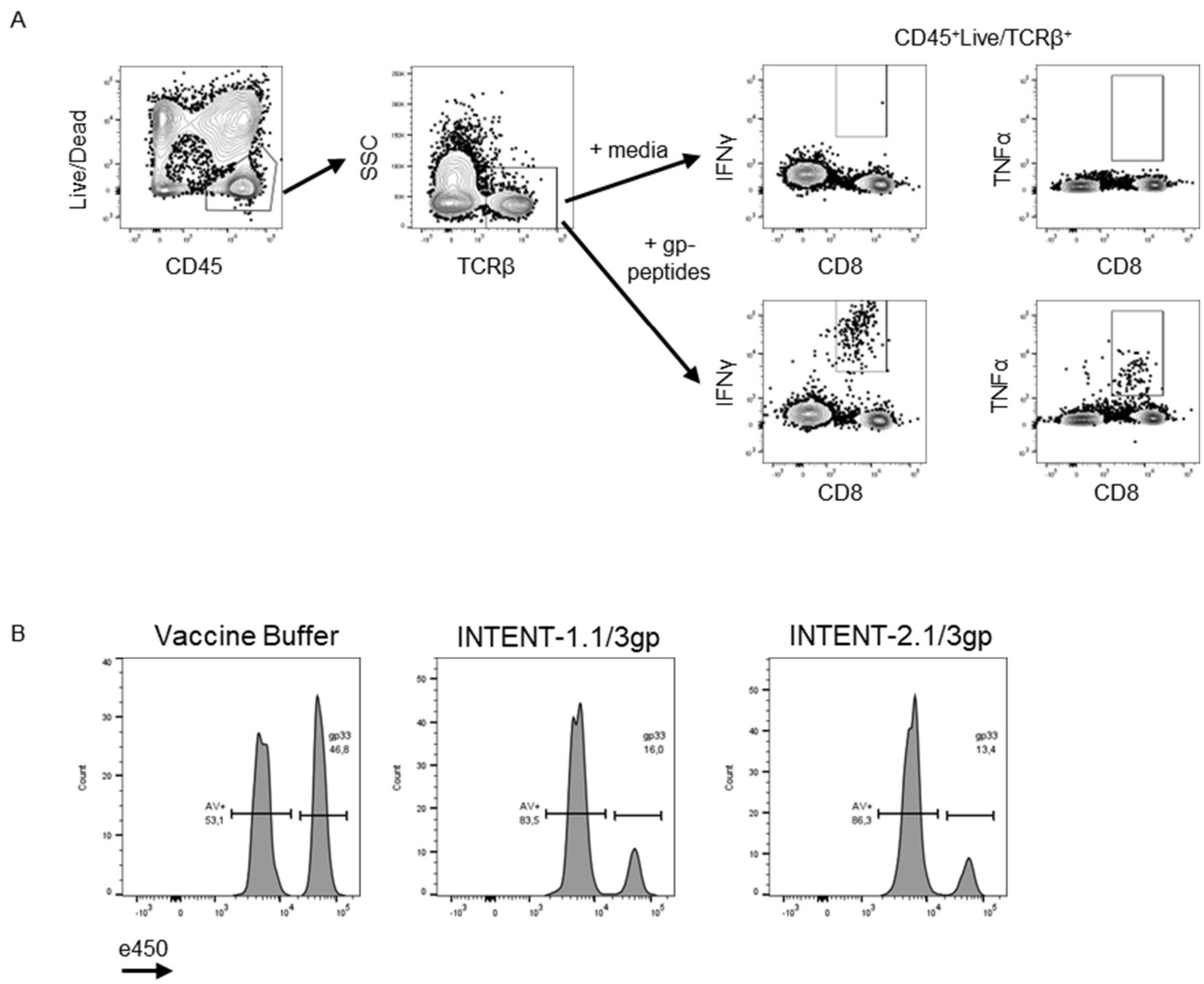

Figure S13 – gp-peptide re-stimulation ICS & CTL assay

(A) C57BL/6J mice were injected on days 0 and 4 with INTENT-2.1/3gp and splenocytes were collected on day 8. Cells were cultured in media alone or with the addition of gp33, gp61 and gp276 peptides (1µg/mL of each) in the presence of BrefeldinA and monensin and cultured at 37°C for 4-6 hours. Representative flow plot and gating of IFN-γ and TNF-α producing $CD8^+$ T cells. (B) Representative flow plot of splenocytes harvested from buffer or INTENT treated C57BL/6 mice following transfer of AV ($e450^{dim}$) or gp33 ($e450^{bright}$)-peptide pulsed target cells.

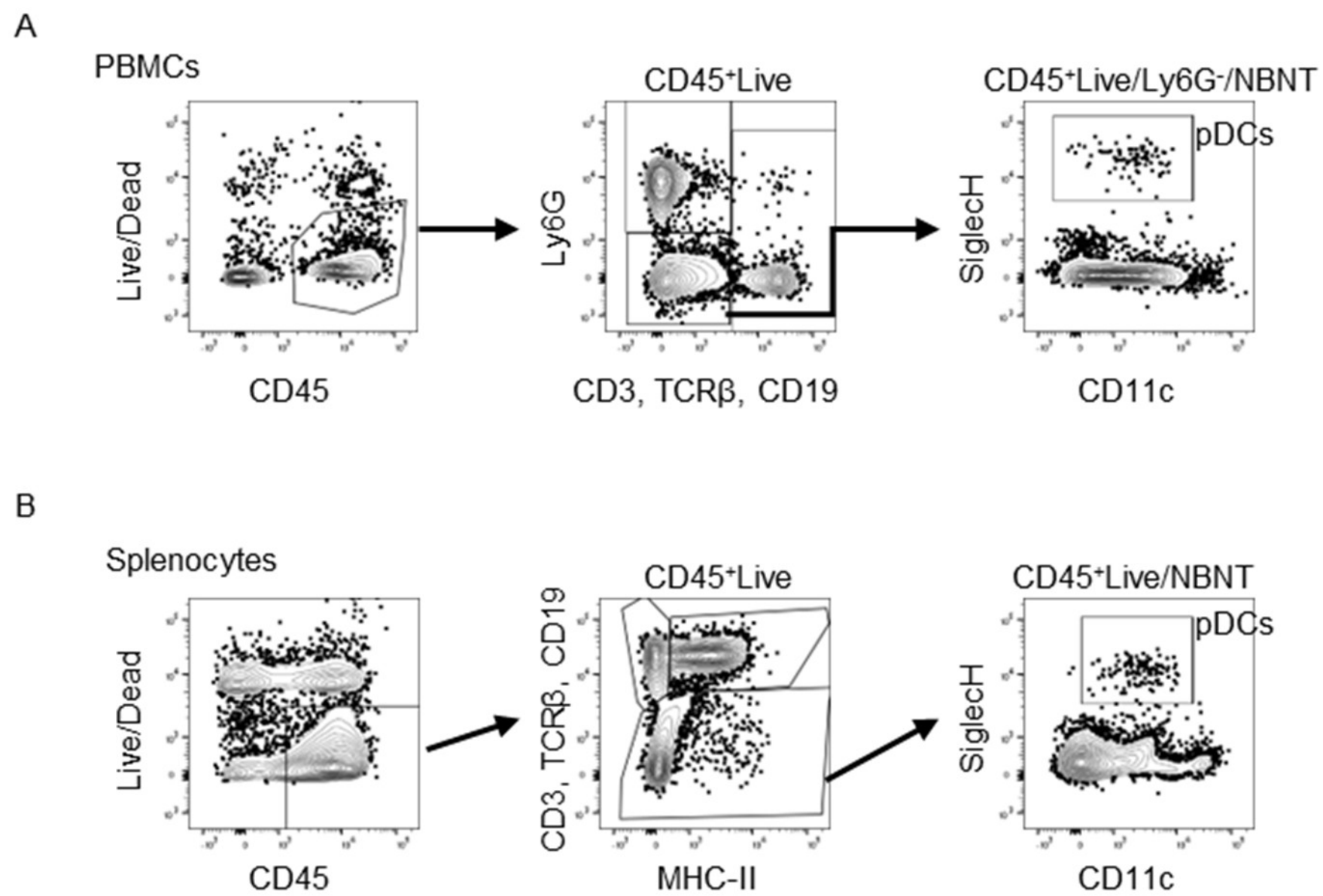

Figure S14 – Representative pDC gating

pDC frequency in the (A) peripheral blood and (B) splenocytes was quantified by flow cytometry and identified as SiglecH$^+$CD11c^{mid} and lacking expression of neutrophil (Ly6G) and B and T cell markers (NBNT).

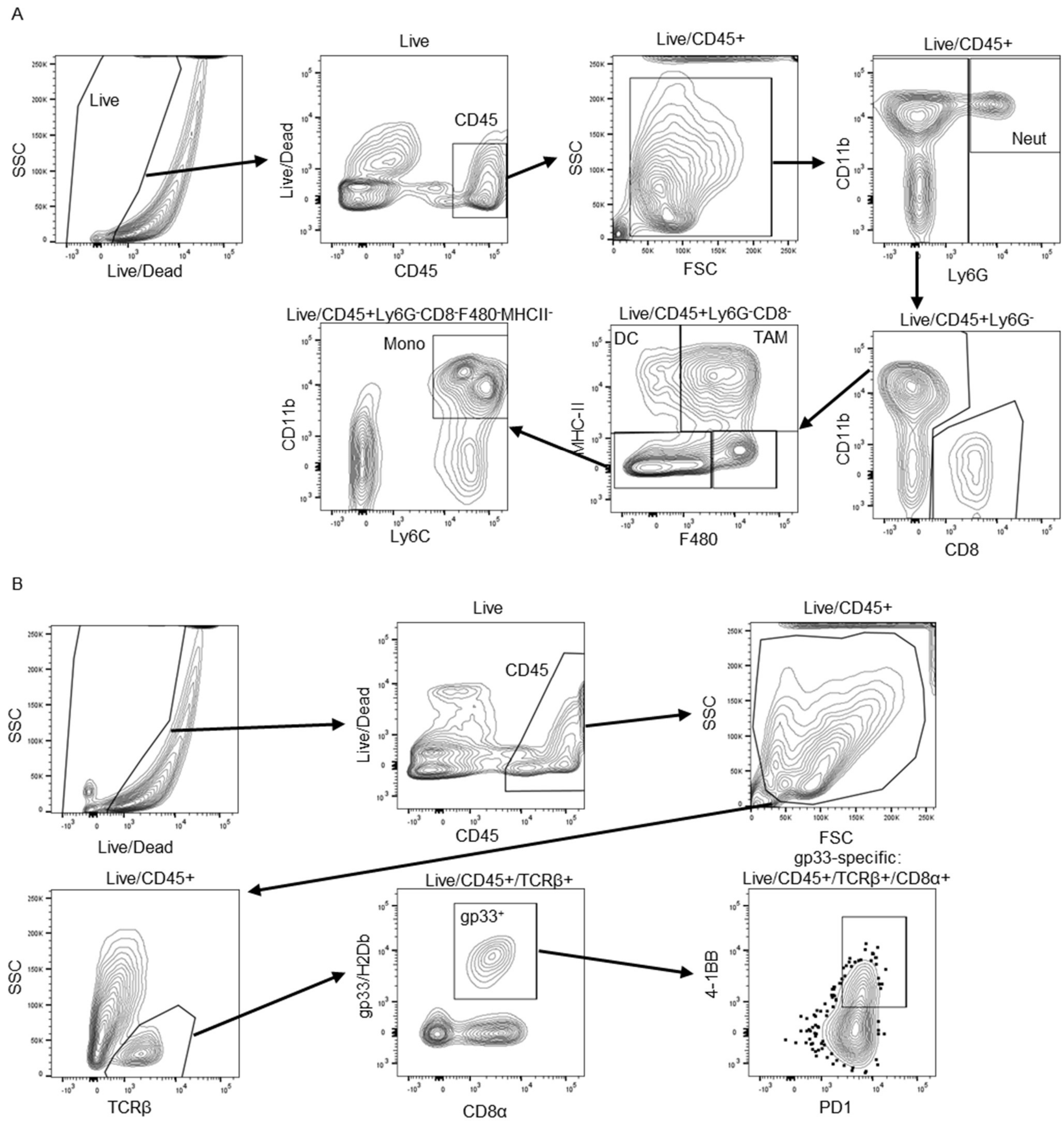

Figure S15 – Representative gating of MC38gp TME

Representative flow gating from cells isolated from MC38gp tumors with gating for (A) myeloid cells including neutrophils (Neut), tumor associated macrophages (TAM), dendritic cells (DC) and monocytes (Mono).(B) 4-1BB expression on gp33-specific CD8+ tumor infiltrating lymphocytes (TIL).
