# Supplementary material for: Novel lipid nanoparticle in a mRNA cancer vaccine drives tumor control via type I IFNs and effector CD8+ T cells
Source: Mol Ther Nucleic Acids. 2026 Aug 27;37(4):103076. doi: 10.1016/j.omtn.2026.103076 (PMC13594926; doi:10.1016/j.omtn.2026.103076)
Supplement: Supplementary file 2 [file mmc2.pdf]

Molecular Therapy
Nucleic Acids
Original Article

Novel lipid nanoparticle in a mRNA cancer vaccine drives tumor control via type I IFNs and effector CD8$^+$ T cells

Matthew J. Gold,[1,4] Douglas G. Millar,[2,4] Jun Liu,[1] Haritha Menon,[1] Yury Karpov,[1] Kirstin Olsen,[1] Liliam Teixeira Oliveira,[1] Charlotte Dubé,[1] Charles W. Tran,[3] Alisha R. Elford,[2] Jordan A. Schwartz,[1] Rajesh Krishnan Gopalakrishna Panicker,[1] Eric G. Marcusson,[1] Pamela S. Ohashi,[2,3] and Natalia Martin Orozco[1]

[1]Providence Therapeutics Holdings, 8832 Blackfoot Trail SE, Ste 120, Calgary, AB T2J 3J1, Canada; [2]Tumor Immunotherapy Program, Princess Margaret Cancer Centre, 610 University Avenue, Toronto, ON M5G 2M9, Canada; [3]Department of Immunology, University of Toronto, Toronto, ON, Canada

mRNA based vaccines utilizing lipid-based delivery systems have been used for cancer therapy with some success targeting tumor-associated antigens (TAAs) or neoantigens. However, in many patients, T cell exhaustion or tumor suppression impedes the induction of a protective anti-tumor T cell response, which suggests that additional activation signals are required to reduce antagonistic tumor effects and reinvigorate the T cells. Here we sought to evaluate novel ionizable lipids for lipid nanoparticle (LNP)-formulations containing a tumor antigen mRNA as a therapeutic treatment for solid tumors and identify key immunogenic features of successful ionizable lipid candidates. Focusing on the induction of a tissue-destructive immune response became the ideal *in vivo* screening to compare two novel ionizable lipids, INTENT-1 and INTENT-2. Both LNPs carrying the same mRNA- encoded antigen induced similar CD8$^+$ T cell responses, but only INTENT-2.1 LNPs could cause tissue destruction. Mechanism-of-action studies revealed that INTENT-2.1, but not INTENT-1.1 LNP, induced type I interferons independently of the mRNA to enhance anti-tissue CD8$^+$ T cell responses. These results demonstrate the efficacy of INTENT-2.1 LNP for cancer vaccines, shed light on its mechanism of action, and support its use in clinical trials to treat solid tumors.

INTRODUCTION

Messenger RNA (mRNA)-based vaccines have proven to be effective at inducing both humoral and cell-mediated immune responses with the introduction of approved vaccines against severe acute respiratory syndrome coronavirus 2 (SARS-CoV2).[1,2] In an oncology setting, mRNA vaccines expressing tumor antigens offer several advantages over other platforms, such as DNA or peptide vaccines.[3–7] DNA-based vaccines are poorly immunogenic and require cellular transfection and translocation to the nucleus, an inefficient process that comes with the risk of insertional mutagenesis.[8] Peptide vaccines are poorly immunogenic and require the addition of adjuvants such as aluminum salts or MF59 to evoke an effective immune response.[9,10] mRNA vaccines overcome several of these obstacles, they can be rapidly synthesized and tailored to individual patient tumor antigen expression as well as be strongly immunogenic through the production of type I IFNs (IFN-I). The encapsulation of mRNA into lipid nanoparticles (LNPs) further improves its stability as well as aiding the uptake by host cells prior to endosomal escape mechanisms allowing mRNA release into the cytosol.[11]

LNPs are comprise four main components: an ionizable lipid for complexing with mRNA and initiating endosomal escape, cholesterol and helper lipid such as 1,2-distearoyl-sn-glycero-3-phosphocholine (DSPC) for providing structure stability, and a polyethylene glycol (PEG)-linked lipid to improve stability and half-life.[12,13] The ionizable lipid component, in addition to the route of injection, influence the biodistribution of the LNPs. Ionizable lipids such as Dlin-MC3-DMA (used in ONPATTRO) and ALC-0315 (used in COMIRNATY: **4-hydroxybutyl)azanediyl]di(hexane-6,1-diyl) bis(2-hexyldecanoate**) result in a primary liver accumulation of mRNA when delivered intravenously (i.v.), in part through their association with ApoE facilitating endocytosis by hepatocytes via the binding to LDL-R.[14] For priming T cell responses desired for cancer immunity, biodistribution to lymphoid organs may facilitate stronger and more efficacious T cell immunity.[15,16] The ionizable lipid component has been found to be highly pro-inflammatory and is thus responsible for the adjuvant activity of LNP vaccines, particularly those utilizing nucleoside-modified mRNAs lacking the type I IFN response elicited by unmodified mRNAs critical for effective anti-tumor immunity.[17] While LNPs have been found to elicit IL-1 and IL-6 cytokines that can support germinal center formation facilitating humoral immunity,[18,19] the production of type I IFNs has been found to be important to facilitate CD8$^+$ T cell responses and tumor immunity.[20–23] Stimulation of pattern recognition receptors (PRRs) such as TLRs, retinoic acid-inducible gene I (RIG-I)-like

Received 7 August 2025; accepted 26 August 2026;
https://doi.org/10.1016/j.omtn.2026.103076.

[4]These authors contributed equally

Correspondence: Natalia Martin Orozco, Providence Therapeutics Holdings, 8832 Blackfoot Trail SE, Ste 120, Calgary, AB T2J 3J1, Canada.
E-mail: natalia@providencetherapeutics.com

Published by Elsevier Inc. on behalf of The American Society of Gene and Cell Therapy.
This is an open access article under the CC BY license (http://creativecommons.org/licenses/by/4.0/).

receptors (RLRs)[24] and cyclic guanosine monophosphate-adenosine monophosphate (GMP-AMP) synthase (cGAS) in coordination with stimulator of interferon genes (STING) can all promote production of type I IFNs. While the induction of type I IFNs by unmodified RNA is well characterized,[25] the mechanism by which ionizable lipids and LNP formulations can stimulate type I IFN production is not completely understood. There are reports of STING activation and induction of IFNs independent of the DNA sensing agent cGAS,[26] and some ionizable lipids have been shown to stimulate STING in a cGAS dependent or independent manner,[16,23] while other LNP formulations induce T cell responses dependent on IFN-Is mediated by the MDA5 pathway.[20] Adjuvants that drive IFN-I production are often used for cancer vaccines and are associated with anti-tumor immunity; however, chronic IFN signaling can lead to immunosuppression.[27–30] Effective cancer vaccines against tumor-associated antigens (TAAs) such as cancer testis antigens (CTAs)[31–33] requires the breaking of tolerance of T cells that have undergone central and peripheral tolerance to these "self" antigens. Induction of an effective anti-self-tissue T cell response likely requires specific adjuvant signals tailored for anti-tumor immunity than those required for a neutralizing antibody response against an infectious agent, suggesting selection of the ionizable lipid for its adjuvant and biodistribution effects should be carefully performed for the desired end use.

Here, we describe an *in vivo* screening platform of INTENT ionizable lipid candidates to select LNP formulations capable of breaking immunological ignorance to a self-antigen. We show that both candidates are capable of rapidly expanding an antigen-specific $CD8^+$ T cell pool; however, only LNPs that induce type I IFNs promote an environment that allow $CD8^+$ T cell-mediated tissue or tumor destruction. This was accompanied by upregulation of MHC-I on the target tissue, allowing TCR-pMHC interaction and cytotoxic T lymphocyte (CTL) activity. LNP-mediated induction of IFN-I was also found to significantly alter the tumor microenvironment (TME), with an increase in tumor-associated neutrophil (TAN) content and in 4-1BB expression on LNP-elicited antigen-specific $CD8^+$ T cells. These findings highlight a novel lipid adjuvant effect with broad implications for imparting therapeutic benefit of antigen-specific $CD8^+$ T cells in response to mRNA vaccination.

RESULTS

mRNA-LNP formulation candidates similarly induce T cell function

Tumor antigen-specific T cells, especially $CD8^+$ T cells, are pivotal in killing tumor cells and thus indispensable for effective cancer immunotherapies.[34] To assess the induction of T cell responses by our LNPs, model antigenic epitopes from the lymphocytic choriomeningitis virus glycoprotein (LCMV gp), including $gp33_{33\text{-}41}$, $gp61_{61\text{-}80}$, and $gp276_{276\text{-}286}$, denoted as 3gp,[35–37] were encoded into an mRNA produced with N1-methylpseudouridine and purified from dsRNA contamination to increase antigen translation. The 3gp mRNA was formulated into LNPs containing novel INTENT ionizable lipids. LNPs were formulated with either the INTENT-1 lipid, to form INTENT-1.1/3gp LNP, or the INTENT-2 lipid to form INTENT-2.1/3gp LNP. C57BL/6 mice were immunized intramuscularly (i.m.) on day 0 and 4 and the resulting induction of antigen-specific $CD8^+$ T cell responses in the peripheral blood was evaluated on day 8 post-injection by tetramer staining (Figure 1A). The greatest T cell response was found to be $H\text{-}2D^b$-restricted and $gp33_{33\text{-}41}$-specific, with both INTENT-1.1/3gp and INTENT-2.1/3gp inducing a significant increase in antigen-specific $CD8^+$ T cells compared to control (vaccine buffer-injected animals, Figure 1B). INTENT-2.1/3gp induced a significant response as well to an epitope overlapping with $gp33_{33\text{-}41}$ but instead presented by $H\text{-}2K^b$, $gp34_{34\text{-}41}$ (Figure 1C). To further explore the induction of antigen-specific $CD8^+$ T cells, we collected both lymphoid and non-lymphoid tissues following INTENT LNP administration. Both INTENT-1.1/3gp and INTENT-2.1/3gp induced a significant expansion of gp33-specific $CD8^+$ T cells in the blood, spleen, draining inguinal lymph nodes (iLNs) and lungs (Figure 1D). INTENT-1.1/3gp induced a significantly higher level of gp33-specific $CD8^+$ T cells in the liver than INTENT-2.1/3gp but levels were otherwise equivalent in the blood, spleen, iLN, and lung (Figure 1D). Following peptide re-stimulation *ex vivo*, T cells from vaccinated animals produced increased amounts of IFN-γ and TNF-α, with INTENT-1.1/3gp inducing significantly higher levels of $IFN\text{-}\gamma^+$ $CD8^+$ T cells than INTENT-2.1/3gp (Figures 1E and 1F). The cytolytic capacity of the LNP-elicited T cells was then evaluated utilizing an *in vivo* killing assay for CTLs. Animals vaccinated with INTENT-1.1/3gp or INTENT-2.1/3gp both showed significant and comparable lysis of adoptively transferred gp33-peptide-pulsed target cells compared to non-specific AV-peptide-pulsed target cells collected from the spleen (Figure 1G), suggesting both LNP formulations can elicit functional antigen-specific cytotoxic T cells. Body weight was monitored after the first LNP injection to assess the overall health status of the animals and INTENT-2.1/3gp induced a modest yet significant drop in body weight of 10% at 24 h post-injection but mice recovered to pre-dosing weight by day 4, while INTENT-1.1/3gp had negligible effects on body weight (Figure S1). The safety of INTENT-2.1-formulated LNPs was evaluated in a dose-range-finding tolerability study in rats, which confirmed that INTENT-2.1-formulated LNPs are well tolerated in animals at high doses, with no vaccine-related mortality or abnormal findings upon macroscopic examination of tissues and organs (Table S4). The activation of dendritic cell (DC) populations 24 h post-injection in the draining iLN and spleen found INTENT-2.1/3gp LNP induced a significant increase in CD80 expression in both cDC1 and cDC2 populations (Figure S1B). Biodistribution of LNPs formulated with luciferase (Luc) mRNA was conducted and found similar expression and kinetics of INTENT-1.1/Luc and INTENT-2.1/Luc LNPs in the muscle (injection site) and abdomen (Figure S2); however, the expression at these sites was significantly lower than that observed with a control LNP formulated with the ionizable lipid SM-102.

INTENT-2.1 LNPs induce autoimmunity in RIP-gp transgenic mice

Recent clinical evaluation of therapeutic cancer vaccines has demonstrated success at inducing antigen-specific $CD8^+$ T cells responses,[5,38]

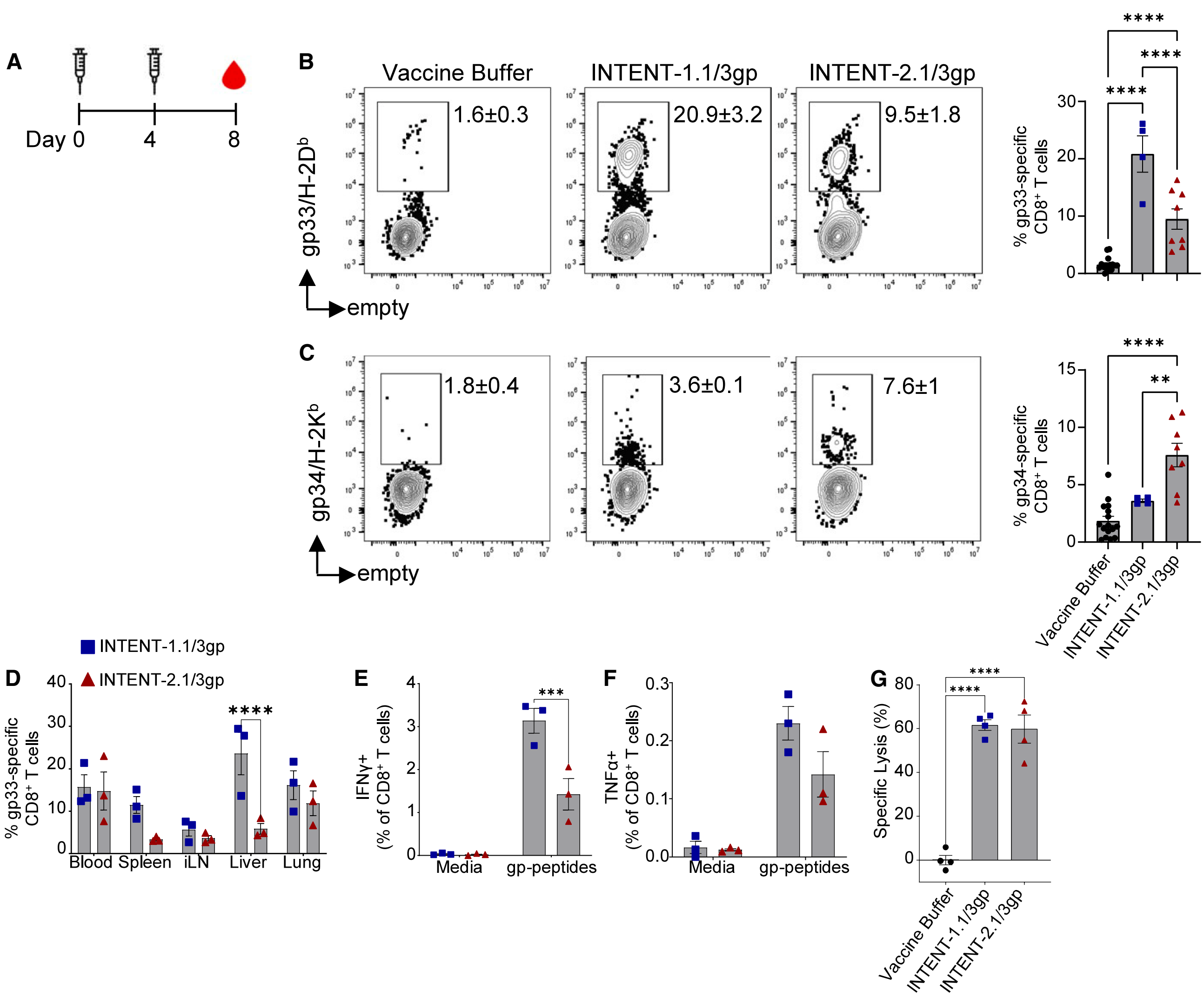

Figure 1. INTENT LNPs induce CD8⁺ T cell responses

(A) Experimental timeline of LNP administration. Naive C57BL/6 mice were injected i.m. with 12.5 μg/50 μL of INTENT LNP or vaccine formulation buffer on days 0 and 4. On day 8, peripheral blood was collected and incubated with tetramers to (B) gp33 or (C) gp34 (n = 4–16). (D) On day 8, the indicated tissues were collected, dissociated, and incubated with tetramers to gp33 prior to flow cytometry (n = 3). Splenocytes were collected on day 8 and re-stimulated with gp-peptides and production of (E) IFNγ or (F) TNFα was quantified by flow cytometry (n = 3, representative gating shown in Figure S13A). (G) On day 8, immunized animals were injected with gp33 or AV-pulsed target cells i.v. and 4 h later, splenocytes were collected to evaluate lysis of the target cells (n = 4, representative gating in Figure S13B). Data are shown as mean ± SEM and analyzed by one-way or two-way ANOVA followed by Tukey's multiple comparison test, *$p < 0.05$, **$p < 0.01$, ***$p < 0.001$, ****$p < 0.0001$.

yet these responses do not always correlate with improved survival. The non-responders in these studies could reflect strong tolerance to tumor antigens or other immunosuppressive mechanisms. Therefore, we decided to test the new formulations in an *in vivo* system that could reveal LNP potency at inducing T cell responses that can overcome immunological ignorance. INTENT-1.1/3gp and INTENT-2.1/3gp LNPs were examined for their ability to stimulate T cell-mediated immunity in the rat insulin promoter (RIP)-gp transgenic mouse model. In this model, LCMV gp is expressed in the pancreatic islet β cells under control of the RIP.[39] Strong antigen stimulation, such as LCMV infection or administration of gp-peptides with TLR agonists such as CpG or polyI:C, can break the immune ignorance and induce cytotoxic gp-specific T cells that kill the insulin-producing β cells. This results in an increase in blood glucose that can be measured over time.[40] RIP-gp mice were administered LNP or buffer control on days 0, 4, and 18 and blood samples were collected for glucose measurements (Figure 2A). As in the C57BL/6 mice (Figure 1B), INTENT-1.1/3gp induced significantly more circulating gp33-specific CD8⁺ T cells in the peripheral blood collected on day 7 than INTENT-2.1/3gp (Figure 2B). Interestingly, vaccination of

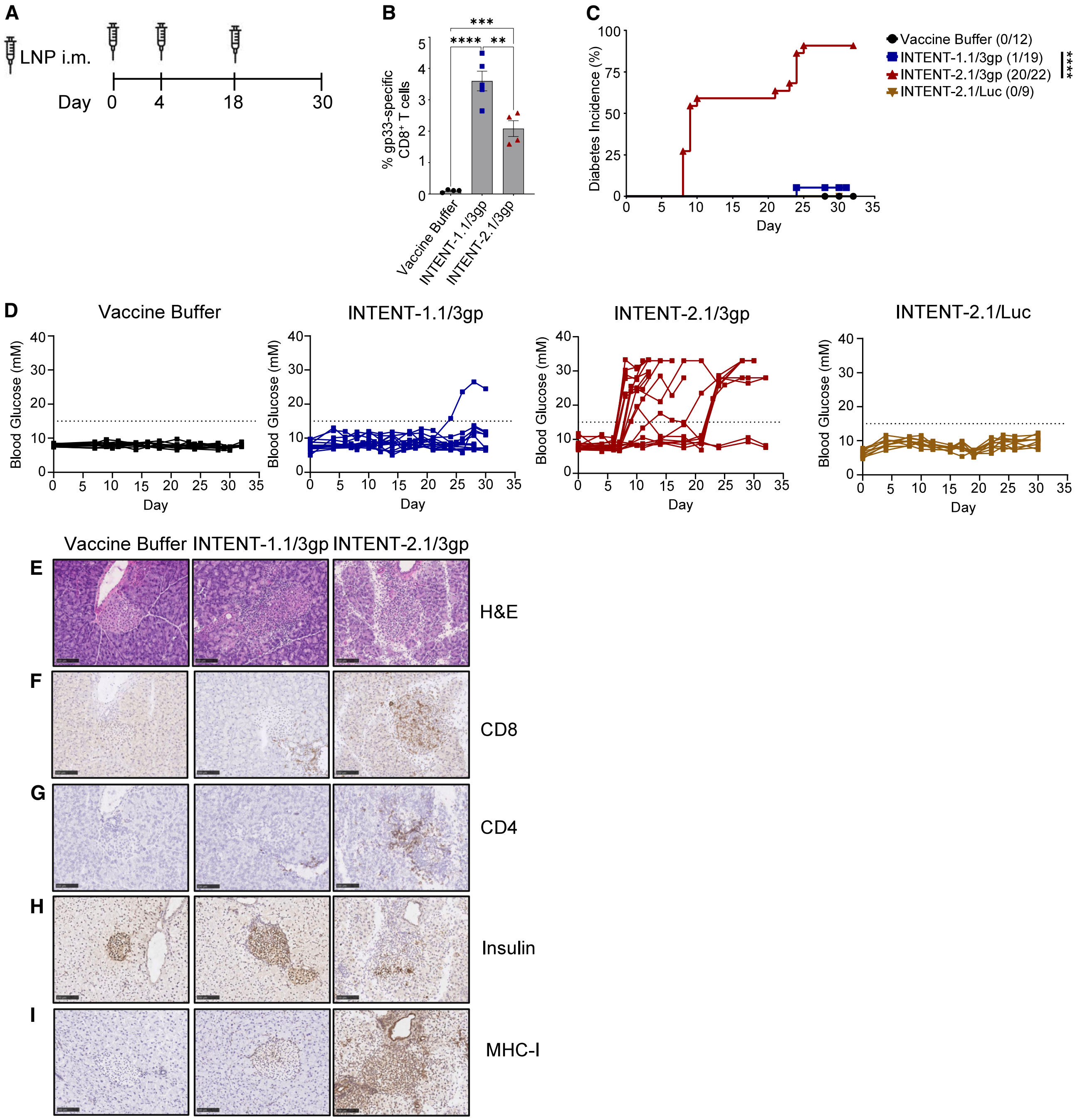

Figure 2. INTENT-2.1/3gp breaks immunological ignorance in RIP-gp mice

(A) RIP-gp mice were administered i.m. with 12.5 μg/50 μL INTENT LNPs or vaccine formulation buffer on days 0, 4, and 18 and peripheral blood glucose was measured 2–3 times per week. (B) On day 7, peripheral blood was sampled and gp33-specific $CD8^+$ T cells were quantified by flow cytometry (*n* = 4). (C) Diabetes incidence from RIP-gp mice administered with the indicated INTENT LNPs, the number of the total treated animals per group and the animals becoming diabetic are shown in parentheses. (D) Blood glucose measurements from RIP-gp mice vaccinated with the indicated INTENT LNP, each line represents an individual mouse. (E–I) Pancreas tissues were collected from RIP-gp mice on day 7 following the indicated treatment and subjected to (E) H&E or IHC for (F) CD8, (G) CD4, (H) Insulin or (I) MHC-I staining, scale bars, 100 μm. Data are shown as mean ± SEM and analyzed by one-way ANOVA followed by Tukey's multiple comparison test or Log rank test, $^{**}p < 0.01$, $^{***}p < 0.001$, $^{****}p < 0.0001$.

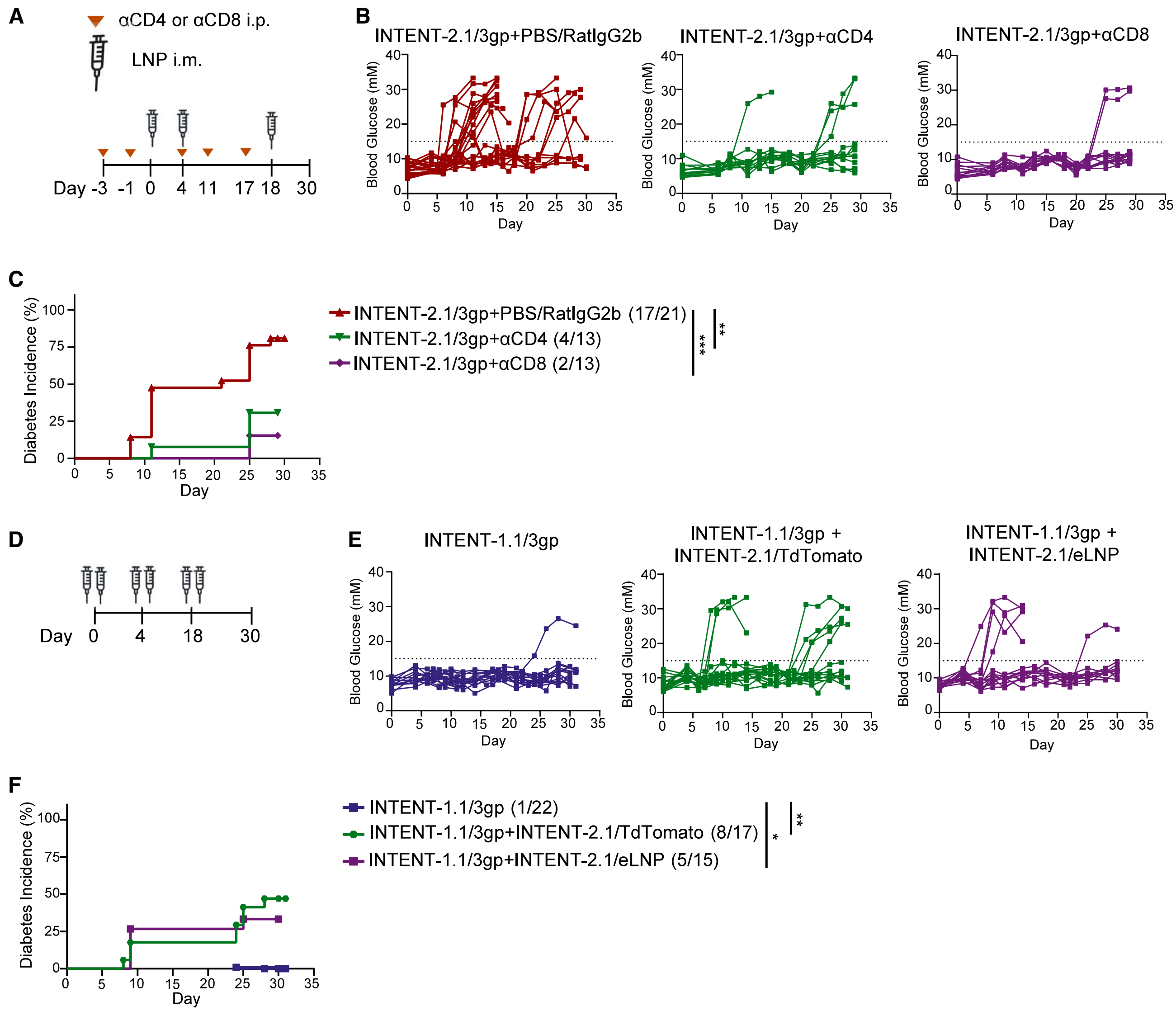

Figure 3. T cell dependence and adjuvant activity in INTENT-2.1-mediated diabetes incidence of RIP-gp mice

(A) RIP-gp mice were administered isotype (Rat IgG2b) or depletion antibodies for CD4 or CD8 i.p. on the indicated dates prior to and during INTENT-2.1/3gp vaccination (12.5 μg/50 μL i.m.) and blood glucose was measured 2–3 times per week. (B) Blood glucose measurements from RIP-gp animals with the indicated treatment, each line represents an individual mouse. (C) Diabetes incidence from RIP-gp animals administered the indicated treatment, the number of the total treated animals per group and the animals becoming diabetic are shown in parentheses. (D) RIP-gp mice were administered INTENT-1.1/3gp either alone or with the indicated INTENT-2.1 LNP at separate injection sites in the same muscle tissue (ipsilateral) on days 0, 4, and 18. (E) Blood glucose measurements from RIP-gp animals with the indicated treatment, each line represents an individual mouse. (F) Diabetes incidence from RIP-gp animals as in (E), the number of the total treated animals per group and the animals becoming diabetic are shown in parentheses. Compiled INTENT-1.1/3gp RIP-gp data with Figures 2C and 2D. Statistics were performed using a Log rank test, *$p < 0.05$, **$p < 0.01$, and ***$p < 0.001$.

the RIP-gp mice with INTENT-2.1/3gp, but not INTENT-1.1/3gp, caused diabetes as exemplified by a persistent increase of blood glucose levels (≥15 mM, Figures 2C and 2D). The diabetes incidence following INTENT-2.1/3gp administration exceeds that of DC vaccination and is similar to rates seen with LCMV infection, suggestive of an immune response effective at breaking immunological ignorance without viral or cellular addition.[41] LNPs formulated with INTENT-2 but containing a non-specific mRNA (Luc, INTENT-2.1/Luc) administered to RIP-gp mice failed to elicit any detectable circulating gp33-specific $CD8^+$ T cells and the mice maintained a normal glucose level (Figures 2C and 2D). Histological analysis of the pancreas showed that pancreatic islets from INTENT-2.1/3gp-vaccinated RIP-gp mice were infiltrated with abundant $CD4^+$ and $CD8^+$ T cells, while infiltration following INTENT-1.1/3gp vaccination was primarily localized

perivascularly (Figures 2E–2G). This T cell infiltration in the islets resulted in the loss of insulin production, as immunostaining indicated abundant insulin-producing islet cells remaining in buffer or INTENT-1.1/3gp-treated mice, while they were mostly destroyed by day 7 following administration of INTENT-2.1/3gp (Figure 2H). The inability for INTENT-1.1/3gp to induce a positive response was remarkable, as they induce comparable or higher frequency of gp-specific $CD8^+$ T cells with equivalent CTL activity to INTENT-2.1/3gp (Figures 1B–1G and 2B). Previous studies have shown that the presence of antigen-specific $CD8^+$ T cells in isolation is insufficient to induce diabetes unless accompanied by upregulation of MHC-I expression on pancreatic islet β cells.[40] Consistent with the previous findings, we found that INTENT-2.1/3gp, but not INTENT-1.1/3gp, could upregulate MHC-I expression on islet β cells (Figure 2I), allowing their recognition and destruction by gp-specific $CD8^+$ T cells. To determine the role of T cells in INTENT-2.1/3gp-induced tissue destruction, antibodies were delivered to selectively deplete either $CD4^+$ or $CD8^+$ T cells during INTENT-2.1/3gp treatment, and depletion efficiency was confirmed in the peripheral blood (Figures S3A and S3B). Depletion of $CD4^+$ or $CD8^+$ T cells during INTENT-2.1/3gp vaccination (Figure 3A) significantly reduced the incidence of diabetes, indicating that both $CD4^+$ and $CD8^+$ T cells were involved in producing the autoimmune response against the pancreatic β islet cells (Figures 3B and 3C). Depletion of $CD4^+$ and $CD8^+$ T cells during INTENT-1.1/3gp treatment was also tested. Interestingly, depletion of $CD4^+$ T cells resulted in a significant increase in gp33-specific $CD8^+$ T cell frequency in the blood, but could not induce diabetes in the majority of animals, suggesting INTENT-1.1/3gp-elicited $CD8^+$ T cells in isolation are insufficient in driving an effective response (Figures S3B–S3D). While INTENT-1.1/3gp was incapable of breaking immune ignorance as a single agent, we asked whether the addition of the INTENT-2 ionizable lipid could act as an antigen-independent adjuvant and enhance the T cell response induced by INTENT-1.1/3gp. To test this, INTENT-1.1/3gp was injected either alone or in combination with INTENT-2.1 LNP containing a non-specific mRNA (INTENT-2.1/TdTomato) or as an empty LNP (eLNPs) without mRNA (INTENT-2.1/eLNP, Figure 3D). While INTENT-1.1/3gp alone was insufficient to break immune ignorance, the addition of INTENT-2.1/TdTomato or INTENT-2.1/eLNP as a non-antigen specific adjuvant produced the necessary immune-stimulating signals required for a strong anti-tissue T cell response (Figures 3E and 3F). This suggests that the INTENT-2 ionizable lipid itself has functional adjuvant activity capable of breaking immunological ignorance during an antigen-specific-driven response.

Therapeutic effect of INTENT LNPs in established MC38gp tumors

The therapeutic effect of INTENT-1.1/3gp and INTENT-2.1/3gp formulations were further evaluated in mice with established subcutaneously (s.c.) transplanted tumors from MC38 cells engineered to express LCMV gp_{1-100} (MC38gp, Figure 4A).[42] Administration of INTENT-1.1/3gp or INTENT-2.1/3gp induced a significant population of circulating gp33-specific $CD8^+$ T cells (Figure 4B) and a delay in tumor growth (Figure 4C). However, only treatment with INTENT-2.1/3gp resulted in the complete clearance of established tumors (complete response, CR) in ∼50% of treated animals when delivered as a monotherapy in a therapeutic setting (Figure 4D). Animals treated with INTENT-2.1/3gp that cleared established tumors maintained long-term tumor control and immunity against tumor re-challenge (Figure S4A). INTENT-2.1 LNPs formulated with non-specific Luc mRNA (INTENT-2.1/Luc) displayed no induction of antigen-specific T cells nor tumor control, highlighting the T cell dependence of INTENT-2.1/3gp in mediating tumor control (Figures 4B–4D). To determine the role of $CD4^+$ or $CD8^+$ T cells in the INTENT-2.1/3gp-mediated therapeutic response, antibodies were utilized to deplete either of the cell types during the immunization phase (Figure 4E; Figures S3E and S3F). Depletion of $CD4^+$ T cells alone had a mild but significant therapeutic effect compared to untreated animals (Figures 4F and 4G), likely due to depletion of Tregs.[43] Depletion of $CD4^+$ T cells had limited additional effect in combination with INTENT-2.1/3gp, with both groups having similar tumor growth kinetics and survival (Figures 4F and 4G). Depletion of $CD8^+$ T cells, either alone or in combination with INTENT-2.1/3gp vaccination, resulted in rapid tumor growth equivalent to vaccine buffer-treated animals (Figures 4F and 4G). This suggests that $CD8^+$ T cells play a critical role in vaccine-mediated tumor control, and that while both INTENT-1.1/3gp and INTENT-2.1/3gp induce similar frequencies of tumor-specific $CD8^+$ T cells peripherally, INTENT-2.1/3gp has additional immunogenic effects that potentiates the activity of the vaccine-elicited T cells allowing improved tumor control and clearance. To compare the anti-tumor efficacy of INTENT-2.1 to commercial standards, LNPs were formulated with 3gp mRNA using either ALC-0315 or SM-102 ionizable lipids.[1,2] When tracking changes in body mass as a surrogate marker for inflammation and toxicity, INTENT-2.1/3gp and ALC-0315 had similar minor drops in weight 48 h after injection that resolved within 96 h (Figure S4B). Induction of antigen-specific T cells was equivalent between INTENT-2.1/3gp and ALC-0315/3gp, while SM-102/3gp resulted in a significantly higher frequency (Figure S4C); however, only INTENT-2.1/3gp treatment resulted in a significant delay in tumor growth and complete eradication of tumor burden in some responding animals (Figures S4D and S4E).

Distinct immunogenicity profiles of INTENT ionizable lipids

The ionizable lipid component of LNPs has been shown to be important for the adjuvant activity of LNP vaccinations and responsible for the robust humoral and cell-mediated immunity of these formulations.[19,44,45] To discriminate between the specific innate immune activation induced by administration of either INTENT-1.1 or INTENT-2.1 LNPs, animals were administered with a single dose and changes in mRNA expression of inflammatory mediators was evaluated at 6 and 24 h later in the muscle (injection site), draining iLN and spleen (Figure 5A; Figures S5–S7) while protein levels were quantified in the serum (Figure 5B; Figure S8). While both ionizable lipids induced an increase in expression of multiple inflammatory cytokines, INTENT-2.1/3gp typically resulted in a more rapid and greater response suggestive of a more potent adjuvant activity (Figures 5A–5C; Figures S5–S8). The IL-1 pathway has been reported

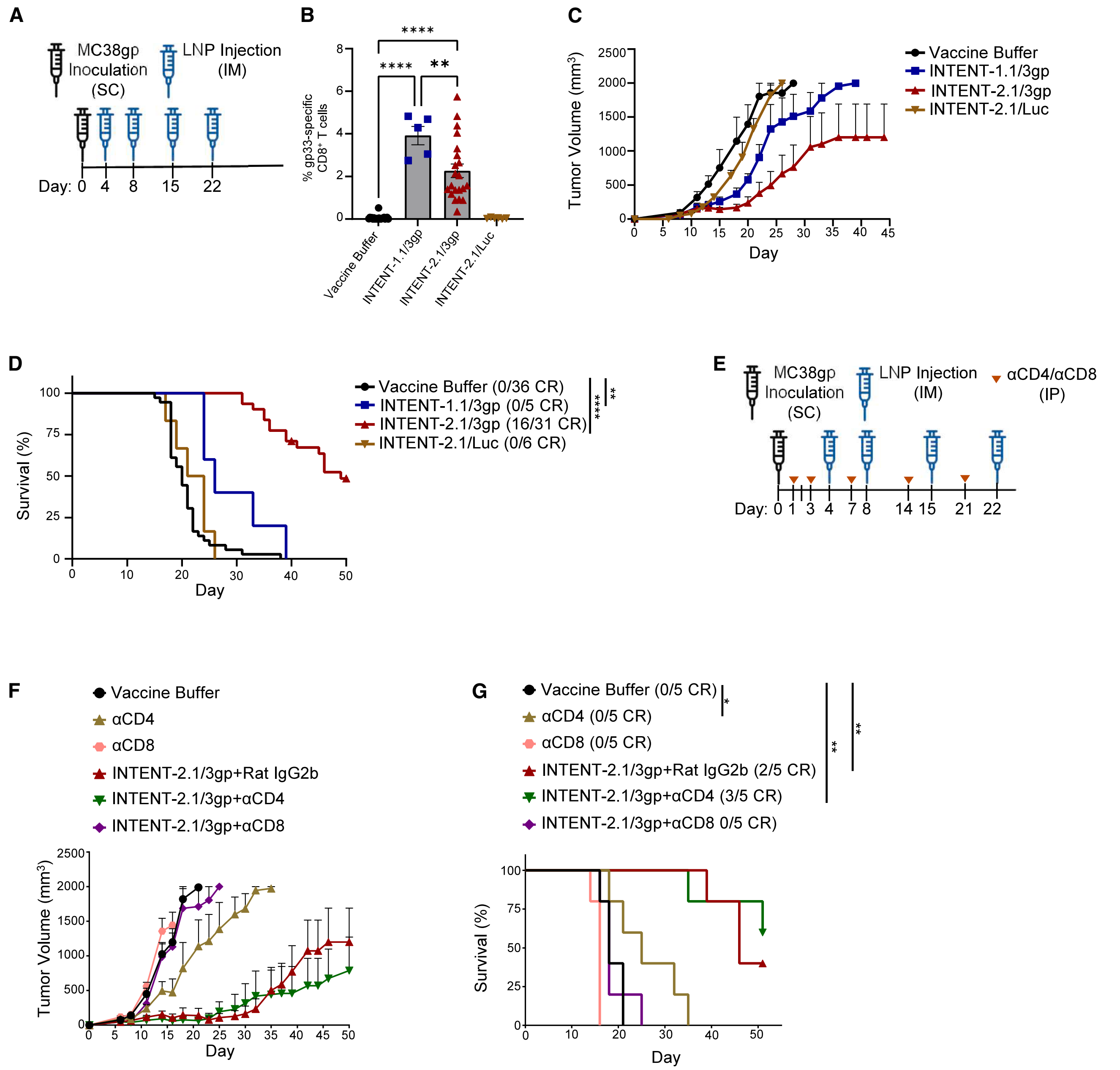

Figure 4. INTENT-2.1/3gp provide therapeutic tumor control in MC38gp solid tumor model

(A) Experimental timeline of MC38gp tumor inoculation on day 0 (1×10^6 s.c.) followed by vaccine formulation buffer or INTENT LNP administration on days 4, 8, 15, and 22 (12.5 μg/50 μL i.m.). (B) Peripheral blood was collected on day 11 and incubated with gp33 tetramers to quantify antigen-specific $CD8^+$ T cells (n = 5–21). (C) Average tumor growth curves for the indicated treatment groups (n = 5–6). (D) Survival percentages for the indicated treatment groups. The number of the total treated animals per group and the animals displaying a complete response (CR) are shown in parentheses. (E) Experimental schedule for subcutaneous MC38gp tumor inoculation and INTENT-2.1/3gp LNP treatment including the schedule of αCD4, αCD8, or isotype (Rat IgG2b) antibody treatment (200 μL i.p.). (F) Average tumor growth curves for the indicated treatment groups (n = 5). (G) Survival percentages for the indicated treatment groups. The number of the total treated animals per group and the animals displaying a CR are shown in parentheses. Statistics were performed using a one-way ANOVA followed by Tukey's multiple comparison test or a Log rank test, $^*p < 0.05$, $^{**}p < 0.01$, $^{****}p < 0.0001$.

to be strongly induced by some ionizable lipids,[18] and we observed that administration of either INTENT LNP induced significantly increased expression of members of the IL-1 pathway (*Il1a*, *Il1b*, and *Il1rn*, Figure 5A; Figures S5–S7). INTENT-2.1/3gp vaccination led to a significant increase in expression of several chemokines responsible for monocyte, neutrophil, and T cell recruitment (*Ccl2*,

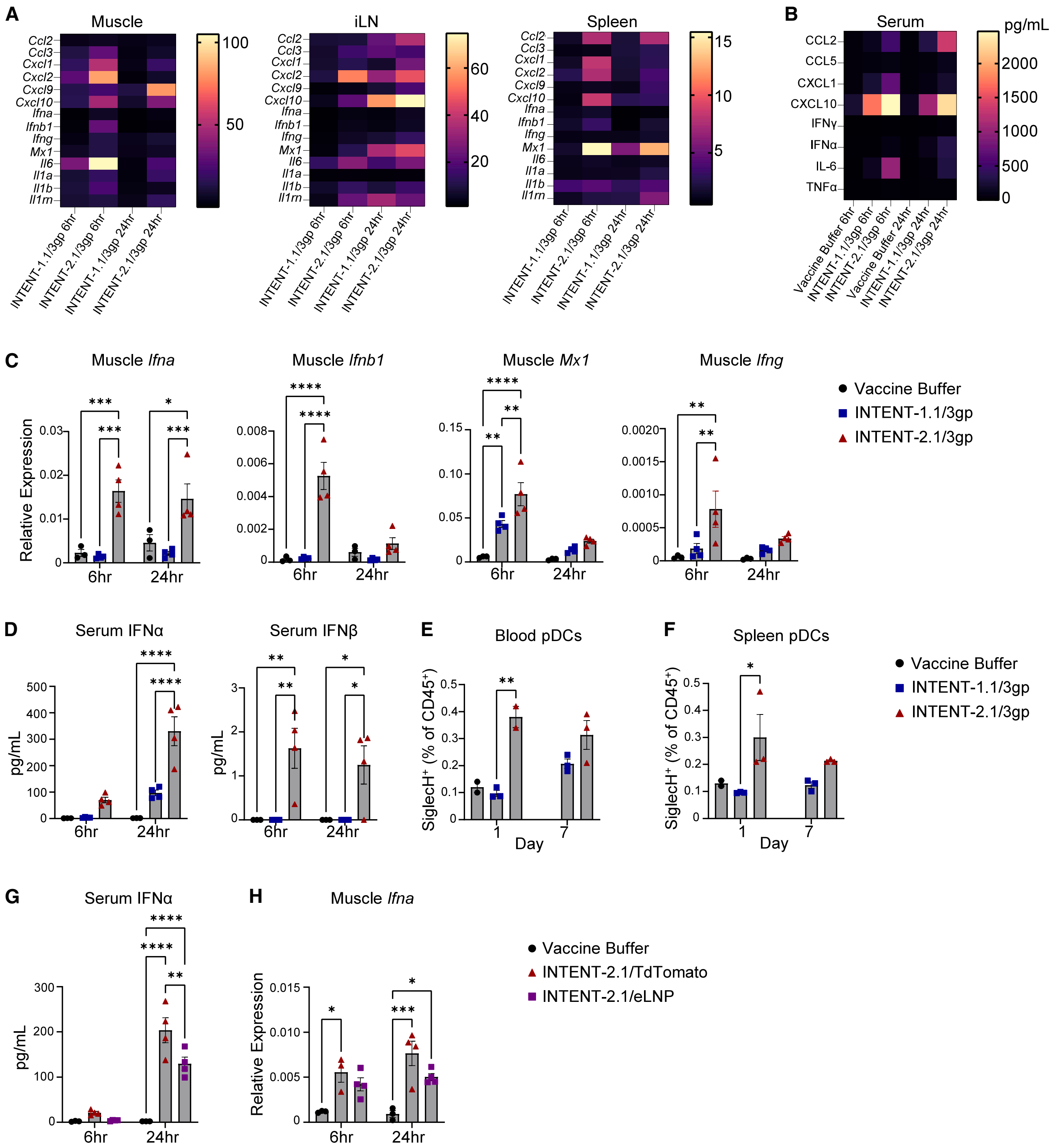

Figure 5. INTENT LNPs stimulates the production of innate cytokines

(A) Heatmap showing the expression of innate cytokine genes measured by quantitative reverse-transcription PCR (RT-qPCR) in the muscle, dLN, and spleen of C57BL/6J mice (n = 3–4) administered buffer, INTENT-1.1/3gp, or INTENT-2.1/3gp 6 and 24 h after i.m. administration, represented as fold increase over vaccine buffer-treated controls (individual RT-qPCR samples shown in Figures S5–S7 are shown as expression relative to *Actb*). (B) Heatmap of the cytokine concentration in the serum was detected by ELISA or multiplex bead array and shown as pg/mL (individual concentration data shown in Figure S8). (C) Relative expression value of *Ifna*, *Ifnb1*, *Ifng* and *Mx1* genes in the muscle at 6 and 24 h (relative to *Actb,* displayed in Figure 5A as fold change). (D) IFNα and IFNβ protein levels measured by ELISA in the serum (as displayed in

(legend continued on next page)

Ccl3, *Cxcl1*, *Cxcl2*, *Cxcl9*, and *Cxcl10*) and IL-6, a cytokine with pleiotropic activities promoting T and B cell responses (Figures 5A–5C; Figures S5–S8).[19] INTENT-2.1/3gp was uniquely capable of inducing a significant type I IFN response, marked by increased expression of *Ifna*, *Ifnb1*, and *Mx1* in the muscle after injection as well as increased serum IFNα and IFNβ (Figures 5C and 5D). INTENT-2.1/3gp also induced an increase in circulating and splenic plasmacytoid DC (pDC) frequency, a cell type that produces IFNα (Figures 5E and 5F). LNPs have previously been found to strongly induce the production of type I IFNs, either through the mRNA or ionizable lipid component.[21,46] While modified mRNA utilized in our studies is purified to remove dsRNA contamination, to confirm the selective role of the ionizable lipid component in the induction of type I IFN INTENT-2.1/eLNP were used. INTENT-2.1 LNPs formulated with or without mRNA induced similar production of IFNα and other cytokines/chemokines (Figures 5G and 5H; Figures S9A–S9C), suggesting the adjuvant activity of INTENT-2.1 LNPs is primarily in response to the lipid, and the INTENT-2 ionizable lipid is capable of driving expression of type I IFNs. We found that INTENT-2.1/3gp induced a similar cytokine profile in the serum and muscle as ALC-0315/3gp and SM-102/3gp LNPs; however, ALC-0315 and SM-102 LNPs induced a stronger response in the lymph nodes, spleen, and liver (Figures S10 and S11). ALC-0315 and SM-102 induced particularly high levels of *Cxcl9*, *Cxcl10*, and *Mx1* in the liver (Figure S11C). While SM-102 LNPs induced a higher biodistribution signal, frequency of antigen-specific T cells and similar cytokine induction post-injection (Figures S2, S4C, S10, and S11), the ability of SM-102/3gp LNP to control tumor growth was significantly lower than INTENT-2.1/3gp (Figures S4D and S4E), suggesting a mechanism driven by local muscle responses unique to INTENT-2.1/3gp that are important for effective anti-tumor immunity.

Type I IFNs are crucial for INTENT-2.1/3gp-mediated anti-tissue responses

Administration of INTENT-2.1/3gp leads to a rapid and significant production of several immune mediators, including IL-6, IFN-γ, and type I IFNs, IFN-α and IFN-β. To determine the relative contribution of each of these cytokines, neutralizing antibodies were delivered in conjunction with INTENT-2.1/3gp immunization in the RIP-gp model (Figure 6A). We found that neutralization of either IL-6 or IFN-γ had limited effect on diabetes incidence following INTENT-2.1/3gp treatment, with IL-6 neutralization slightly increasing the rate of onset of diabetes, suggesting that while these cytokines are produced following immunization, their activity was not required (Figures 6B and 6C). Inhibition of type-I IFNs through the administration of antibodies to the shared IFNα/IFNβ receptor IFNAR-1[47] during INTENT-2.1/3gp immunizations resulted in a complete inhibition of T cell infiltration into the pancreatic islet (Figures 6D–6F) and animals maintained normal blood glucose levels (Figures 6B and 6C). Indeed, neutralization of type I IFNs significantly reduced the induction of IL-6, IFN-γ, and chemokine expression directly following INTENT-2.1/3gp administration (Figure S12). This highlights a critical role of type I IFNs in promoting a T cell-driven anti-tissue response and drives downstream changes in the inflammatory response.

Type I IFN signaling is required for INTENT-2.1/3gp anti-tumor response

To determine the role of IFN-I in INTENT-2.1/3gp-mediated tumor control, MC38gp tumor-bearing animals were treated with INTENT-2.1/3gp in combination with αIFNAR1 or an isotype control (Figure 7A). As seen in the RIP-gp model, blockade of IFN-I signaling completely abolished the therapeutic effect of INTENT-2.1/3gp treatment, with all animals rapidly succumbing to tumor growth with similar kinetics to vaccine buffer-treated animals (Figures 7B and 7C). IFN-Is have pleiotropic effects on a wide array of cell types. To determine the potential mechanism of IFN-I-mediated INTENT-2.1/3gp therapeutic effects, we characterized the tumor infiltrating lymphocyte (TIL) and myeloid populations in MC38gp tumors (Figure 7D). When examining the myeloid compartment, INTENT-2.1/3gp treatment led to a significant increase in (TANs, Figure 7E). A similar increase in neutrophil frequency in the blood and spleen was found following INTENT-2.1/3gp treatment (Figure 7F). Interestingly, while the increase in splenic neutrophil content was independent of IFN-Is, addition of αIFNAR1 significantly reduced their accumulation in the blood and tumor (Figure 7F). MC38gp tumor tissue displayed increased expression of *Cxcl2* following INTENT-2.1/3gp treatment, an important chemokine for neutrophil migration via CXCR2, and blockade of IFN-I led to reduced expression of *Cxcl2* (Figure 7G). Inhibition of IFN-Is alongside INTENT-2.1/3gp also led to an increased representation of tumor-associated macrophages (TAM), while the frequencies of monocytes and DCs were equivalent (Figure 7E). Due to the dependence of $CD8^+$ T cells on INTENT-2.1/3gp-mediated tumor control and a complete lack of tumor control following αIFNAR1 treatment, we further measured the gp33-specific $CD8^+$ TIL. INTENT-2.1/3gp-treated animals had similar frequencies of gp33-specific $CD8^+$ T cells within the tumor as well as in the draining inguinal LN (dLN), blood, and spleen (Figure 7H). While expression of PD-1 was equivalent between the treatment groups and tissues (Figure 7I), gp33-specific $CD8^+$ T cells in αIFNAR1-treated TIL and dLN had a drastically reduced expression of the co-stimulatory molecule 4-1BB/CD137 (Figures 7J and 7K). The increased 4-1BB expression could be related to INTENT-2.1/3gp-mediated increase in MHC-I expression as 4-1BB expression is rapidly increased on tumor-specific T cells following TCR/pMHC binding.[48]

Figure 5B). Frequency of pDCs ($SiglecH^+CD11c^{mid}$) in the (E) blood and (F) spleen was measured by flow cytometry on day 1 or 7 following buffer or INTENT LNP administration (n = 2–3, gating strategy available in Figures S14A and S14B). INTENT-2.1/TdTomato or INTENT-2.1/eLNP was injected i.m. and (G) IFNα protein in the serum was measured by ELISA or (H) *Ifna* expression in the muscle was measured by RT-qPCR at the indicated time points. Statistics were performed by two-way ANOVA followed by Tukey's test, *$p < 0.05$, **$p < 0.01$, ***$p < 0.001$, ****$p < 0.0001$.

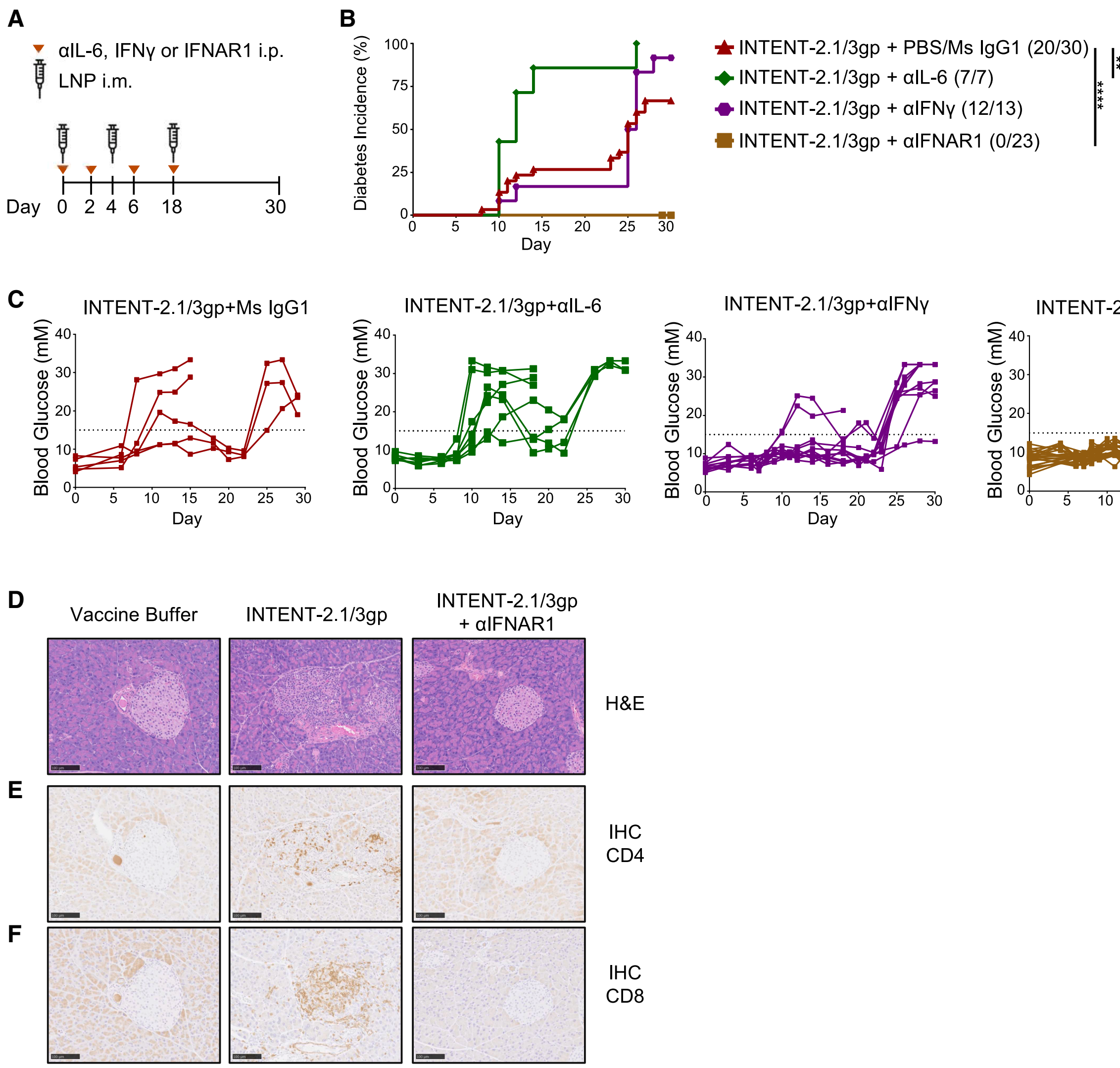

Figure 6. Type I IFNs but not IL-6 or IFN-γ, are required for INTENT-2.1/3gp-induced diabetes in RIP-gp

(A) Experimental outline of INTENT-2.1/3gp immunization and delivery of neutralizing antibodies. (B) Diabetes incidence and (C) blood glucose measurements from RIP-gp animals with the indicated treatment, each line represents an individual mouse. Pancreas tissue was collected on day 9 and processed for (D) H&E, (E) CD4 IHC, and (F) CD8 IHC staining. Scale bars, 100 μm. Statistics were performed using a Log rank test, $^{**}p < 0.01$, $^{****}p < 0.0001$.

DISCUSSION

Therapeutic vaccines utilizing mRNA technology have been shown to be effective at inducing broad $CD8^+$ T cell responses in cancer settings. Unfortunately, this effect has not generally translated well to effective therapeutic anti-tumor capacity. In this paper, we describe a screening method that can identify novel LNPs with the capacity to elicit highly functional tumor-infiltrating $CD8^+$ T cells, postulated to be a key component required for effective therapeutic responses against tumors. An experimental tumor mRNA vaccine formulated with the identified novel ionizable lipid, INTENT-2, controlled tumor growth, and conferred long-term immunity from tumor rechallenge, which is dependent on IFN-I signaling and $CD8^+$ T cells. Careful screening of ionizable lipids or the inclusion of additional adjuvants in the LNP formulation has been proposed to increase the immunogenicity of lipid formulations when utilizing nucleoside-modified mRNA.[22] Here, we report a novel ionizable lipid component of LNPs capable of inducing IFN-α production and with potent anti-tumor activity when complexed with fully modified mRNA encoding tumor-expressed antigens. The $CD8^+$ T cell mediated anti-tissue and anti-tumor response was found to be dependent on IFN-I signaling as responses were blocked with the addition of neutralizing antibodies to IFNAR1 or depletion of $CD8^+$ T cells. It has previously been shown that nucleoside-modified mRNA can induce IFN-α production dependent on the MDA5-MAVS

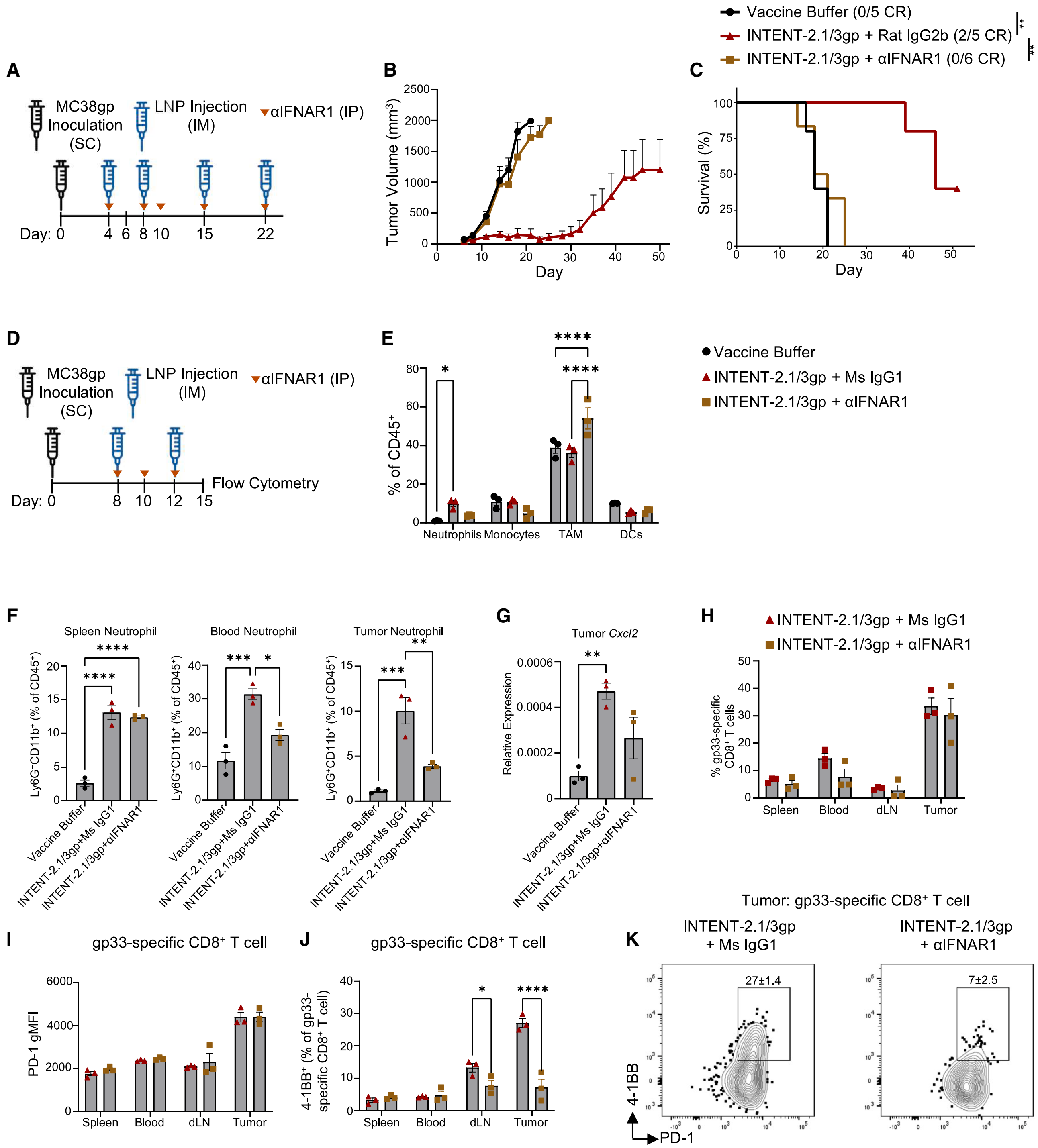

Figure 7. INTENT-2.1/3gp mediates tumor control through type I IFNs and results in increased 4-1BB expression on antigen-specific TIL

(A) Experimental outline of tumor inoculation, INTENT-2.1/3gp, and αIFNAR1 treatment. (B) Average tumor growth and (C) survival percentages for the indicated treatment groups (n = 5–6, vaccine buffer and INTENT-2.1/3gp+Rat IgG2b from Figures 4F and 4G). (D) Experimental outline of tumor inoculation, INTENT-2.1/3gp, and αIFNAR1 treatment used for characterization of tumor infiltrating myeloid and lymphoid populations. (E) Frequency of neutrophils, monocytes, tumor-associated macrophages (TAMs), and dendritic cells (DCs) in MC38gp tumor digests (representative gating strategy in Figure S15A). (F) Frequency of neutrophils (Ly6G⁺CD11b⁺) in the spleen, blood,

(legend continued on next page)

pathway.[20] We have found that INTENT-2.1/eLNPs are still capable of inducing IFN-α secretion. This suggests that a portion of the observed response is likely independent of RNA-sensing PRRs, such as MDA5, and instead due to other mechanisms. Select LNP formulations have been shown to induce the release of DNA into the extracellular space,[49] suggesting some damage-associated PRRs could be responsible for the type I IFN release and could stimulate cytokine production independent of mRNA payloads. Indeed, certain LNP formulations can directly bind to and activate the STING pathway independent of cGAS to elicit production of type I IFNs.[23] Type I IFNs have previously been used directly or induced through TLR/PRR agonists to promote anti-tumor immunity, with pleiotropic activity on a wide array of cell types.[28,29] We found that LNPs formulated with the ionizable lipids ALC-0315 and SM-102 also induced production of IFN-Is, yet did not provide significant tumor control, suggesting other factors unique to INTENT-2.1 LNPs factor into its anti-tumor efficacy. There is growing evidence surrounding the role of non-hematopoietic cells, particularly myocytes, in facilitating antigen presentation following i.m. LNP vaccinations.[50,51] While INTENT-2.1, ALC-0315, and SM-102 have similar immune responses in the muscle, ALC-0315 and SM-102 produce a significant amount of IFN-I-associated inflammatory genes in the liver. Recent reports have suggested that mRNA expression in hepatocytes can suppress anti-tumor T cell responses, suggesting one possible mechanism for the discrepancy in anti-tumor immunity between ALC-0315 and SM-102 LNPs and INTENT-2.1 LNPs.[50] It is unclear what specific mechanism of INTENT-2.1/3gp-elicited IFN-I facilitates the anti-tumor response. One mechanism used by tumor cells as a means of immune evasion is to down-regulate expression of MHC-I molecules.[52] We show that INTENT-2.1/3gp treatment can induce the upregulation of MHC-I on the target tissue, a requirement for $CD8^+$ T cell-mediated recognition and targeted destruction. An early marker of $CD8^+$ T cell recognition of its cognate antigen presented by MHC-I is the upregulation of 4-1BB,[48] and signaling provided by 4-1BB has been shown to play a key role in promoting T cell persistence and anti-tumor immunity.[53,54] It is possible that INTENT-2.1/3gp-induced IFN-I promotes the upregulation of MHC-I and antigen presentation to $CD8^+$ T cells that then increase expression of 4-1BB improving their functionality in the TME.[53] Further phenotypic analysis in future studies will explore the characteristics of INTENT-2.1/3gp-elicited tumor infiltrating lymphocytes, including expression of other co-stimulatory molecules and markers for exhaustion and memory. INTENT-2.1/3gp treatment increases the expression of key neutrophil chemokines such as *Cxcl1/2*, and TAN frequency was significantly increased following INTENT-2.1/3gp treatment. This increased TAN frequency was dependent on type I IFNs as αIFNAR1 treatment significantly reduced their tumor infiltration and mobilization in the blood. It is yet unclear the potential role neutrophils could play in INTENT-2.1/3gp-mediated anti-tumor immunity as they have both pro- and anti-tumor effects likely modulated by the local inflammatory milieu.[55,56] For instance, neutrophil extracellular traps (NETs) have been implicated in facilitating cancer metastasis and reducing T cell mediated cytotoxicity,[56,57] while in other situations the presence of NETs is associated with increased survival and can directly kill tumor cells.[58] TANs have the capacity to directly stimulate T cells and can express important T cell costimulatory molecules such as 4-1BBL, OX40L, and CD86 and secrete cytokines such as IL-12 supporting T cell activation.[59–61] Indeed, such cooperative activity between T cells and neutrophils to eliminate tumors has been reported and will need to be further explored in terms of the specific contribution of neutrophils in INTENT-2.1/3gp-mediated anti-tumor immunity.[62] One of the limitations of this study is that we used a highly immunogenic model antigen of viral origin (LCMVgp), and future studies using low affinity, less immunogenic tumor antigens, such as TAAs and neoantigens are needed to evaluate the potential of INTENT-2.1 LNP in tumor immunotherapy. In summary, we found a novel LNP prepared with INTENT-2, provides exceptional anti-tumor T cell immunity when formulated with a tumor antigen-encoding mRNA in part through the induction of IFN-Is and displays a good safety profile. INTENT-2.1 LNP is being developed for cancer vaccines advancing to clinical testing.

MATERIALS AND METHODS

mRNA synthesis and LNP production

The mRNAs used in these studies encode Luc, TdTomato or a combination of 3 separate epitopes to LCMV separated by cleavable spacers ($gp33_{33-41}$, $gp61_{61-80}$, and $gp276_{276-286}$), flanked by optimized 5′ and 3′ UTRs and a poly(A) tail. The 5′-capped mRNA was produced by an *in vitro* transcription method using T7 RNA polymerase (Roche) and underwent a series of chromatographic purification steps to remove DNA template, nucleotides, enzymes, and dsRNA (double stranded RNA). The purified RNA solution was buffer exchanged into nuclease-free water and concentrated using Amicon columns with 100 kDa molecular weight cutoff, followed by sterile filtration using 0.2 μm filter prior to formulation. The ionizable lipids and LNP used in this study were developed by Providence Therapeutics (and are reported in international patent publication numbers WO2025010504, WO2023115221, and WO2024259531). The mRNA-LNPs were prepared using a process developed in-house. Briefly, mRNA and lipids were mixed using a T-junction developed in-house. The two phases were mixed at a 1:1 flow rate ratio, with a total flow rate of 400–600 mL/min. The resulting mixture was diluted in line with an average of 11 volumes of an aqueous buffer at flow rate of 400–600 mL/min. The mRNA-LNP bulk product was processed by TFF (tangential flow filtration) using a Sartoflow SMART TFF system (Sartorius Stedim North America Inc.) with 300 kDa molecular weight cutoff regenerated cellulose Hydrosart ultrafiltration

and tumor. (G) Expression of *Cxcl2* in the tumor tissue was quantified by RT-qPCR and expressed relative to *Actb*. (H) Frequency of gp33-specific $CD8^+$ T cells in the spleen, blood, draining inguinal LN (dLN), and tumor. (I) gMFI of PD-1 expression and (J) percentage of 4-1BB expression on gp33-specific $CD8^+$ T cells in the indicated tissues (representative gating strategy in Figure S15B). (K) Representative flow cytometry plots of gp33-specific $CD8^+$ T cells collected from the MC38gp tumor. Statistics were performed by one or two-way ANOVA followed by Tukey's multiple comparison test or a Log rank test, *$p < 0.05$, **$p < 0.01$, ***$p < 0.001$ and ****$p < 0.0001$.

cassettes (Sartorius Stedim North America Inc.). The mRNA-LNP bulk product underwent concentration, followed by buffer exchange with 5 diavolumes of the same dilution aqueous buffer used in the previous step to remove residual ethanol and subsequently with 5 diavolumes of 5 mM Tris and 10% sucrose (pH 8.0) storage buffer.[63] eLNPs, mRNA free formulations, were prepared following the same procedures, but excluding the mRNA from the solution mixed with the lipids. The LNPs were sterile filtered using 0.2 μm filter and filled in sterile tubes and stored at −80°C. The standard testing panel of the mRNA-LNPs includes particle size, polydispersity index, percentage of encapsulation, mRNA integrity, mRNA identity, pH, osmolality, and bacterial endotoxin. LNPs formulated with ALC-0315 and SM-102 (heptadecan-9-yl 8-((2-hydroxyethyl)(6-oxo-6-(undecyloxy)hexyl)amino)octanoate) were produced using identical formulations as previously published.[1,2] The LNPs used in this study have similar physicochemical parameters summarized in Table S1.

Animals

All mice were maintained at the University Health Network Animal Resource Center of the Princess Margaret Cancer Center (OCI facility) according to institutional guidelines and with approval of the UHN (University Health Network) Animal Ethics Committee. Female C57BL/6J (strain #: 000664) and BALBc/J (strain #: 000651) mice, 6–8 weeks of age, were purchased from The Jackson Laboratory. RIP-gp transgenic mice were generated and bred as previously described,[39] and both male and female animals were used for diabetes experiments between 8 and 16 weeks of age.

Reagents

LCMV peptides gp33-41 (KAVYNFATM), gp276-286 (SGVENPG GYCL), gp61-80 (GLNGPDIYKGVYQFKSVEFD), and adenoviral (AV, SGPSNTPPEI) peptides were synthesized by New England Peptide (Gardner, MA, USA). Peptide MHC tetramers were produced from biotinylated monomers containing LCMV gp33 bound to H-2D^b, and gp34 bound to H-2K^b, provided by the NIH Tetramer Core Facility. Fluorescently labeled tetramers were produced by gradually combining monomer with Streptavidin-PE or APC (Thermo Fisher Scientific) at a 4:1 molar ratio. Anti-CD8 (clone YTS169) and anti-CD4 (clone YTS191) hybridomas were used for T cell depletion. Antibodies to IL-6 (MP5-20F3), IFN-γ (XMG1.2), and PD-1 (RMP1-14) were purchased from Bio X Cell and IFNAR1 (MAR1-5A3), Rat IgG2b (1–2), and Mouse IgG1 isotype (HKSP84) were purchased from Leinco. Mouse anti-virus response Legendplex (BioLegend) and Mouse IFN-alpha and IFN-beta ELISA kit (PBL Assay Science) were used for serum cytokine measurements.

Immunization and immune monitoring

C57Bl/6J or RIP-gp mice were immunized with mRNA-LNP formulations at the indicated doses in a volume of 50 μL, by i.m. injection of the hind leg (quadriceps femoris), or with formulation buffer alone (10% sucrose, 5 mM Tris pH 8.0), at the indicated time points. Diabetes was monitored three times per week in RIP-gp mice from a drop of blood taken from the lateral tail vein, using glucose test strips and AccuChek II glucose monitor (Roche). Mice with blood glucose levels >15 mM for 2 consecutive readings were considered diabetic. Results from multiple experiments were compiled to determine incidence of diabetes. C57BL/6J mice were inoculated with 1×10^6 MC38gp[42] cells s.c. and tumor growth was monitored three times per week using digital calipers to measure length and width dimensions. Tumor volume calculated according to the equation volume = (length $\times$ width^2)/2 and results from multiple experiments were compiled for survival graphs.

Flow cytometry

Cell preparations from splenocytes were passed through a 70 μm cell strainer, while lymph node, liver, lung, and tumor tissues were digested in a mixture of IMDM (Iscove's Modified Dulbecco's Medium), collagenase IV (Sigma) and DNase (Roche) prior to passing through a cell strainer. Single-cell suspensions and blood samples were lysed of RBCs in ACK buffer (Thermo Fisher Scientific) prior to antibody staining. For peptide restimulation assays, isolated splenocytes were cultured with peptides in the presence of brefeldin A and monensin (BioLegend) in IMDM for 4–6 h and permeabilized (Cytofix/Cytoperm, Becton Dickinson, BD) prior to intracellular antibody staining. Samples were acquired with an LSR Fortessa cytometer (BD Biosciences) and analyzed with FlowJo software (TreeStar). A full list of antibodies are listed in Table S2.

In vivo cytotoxic lymphocyte assay

C57BL/6J mice were injected i.m. with vaccine formulation buffer or INTENT LNPs on days 0 and 4. On day 8, target cells were collected from naive C57BL/6J splenocytes and pulsed with gp33 or a non-specific AV peptide and labeled with high or low concentrations of e450 cell proliferation dye (Thermo Fisher Scientific). Target cells (2–5×10^6) were injected i.v. into immunized mice and animals were euthanized 4 h post transfer and single-cell suspensions from the spleens were stained with propidium iodide (Thermo Fisher Scientific) prior to acquisition on a flow cytometer. Percentage specific lysis = (% AV-labeled cells − % gp33-labeled cells)/(% AV-labeled cells) $\times$ 100.

RNA extraction and qPCR

Tissues were homogenized in Trizol (Thermo Fisher Scientific) using the TissueLyser II (QIAGEN). Reverse transcription was performed using the high-capacity cDNA kit (Thermo Fisher Scientific) and gene-specific primers were utilized with SYBR green (Quanta bio). Primer sequences are listed in Table S3 and values are expressed relative to *Actb*.

Histology

Pancreas tissue was placed in cryomolds (Tissue-Tek) and snap frozen in OCT (optimal cutting temperature compound, Sakura) prior to sectioning 5 μm-thick sections on a cryostat or fixed with 10% formalin prior to paraffin embedding and sectioning. Slides were processed for H&E staining or immunostaining with antibodies against CD4, CD8, insulin, or MHC-I. Primary antibodies were incubated overnight at 4°C followed by secondary and ABC reagent and

development with DAB (3,3'-diaminobenzidine, Vector Laboratories). Slides were counterstained with hematoxylin and scanned on a whole-slide scanner (Nanozoomer, Hamamatsu Photonics) and images acquired using NDPview2 software (Hamamatsu Photonics).

Biodistribution

Female BALB/cJ mice were injected i.m. with the indicated LNPs and 4 or 24 h after LNP injection, luciferin substrate was injected i.p. (IVISbrite, Revvity, ∼2 mg/mouse). Mice were imaged 20 min following substrate injection and anesthetized with isoflurane on a Xenogen IVIS Spectrum, and the luminescence was quantified using the Living Image software (Revvity).

Toxicology

A non-GLP (good laboratory practice) tolerability study was conducted in Sprague-Dawley rats to assess the safety of LNP with identical formulation as INTENT-2.1. This study included a single-dose escalation phase during which rats received doses ranging from 12.5 to 200 μg via i.m. injection followed by a repeated-dose phase of 4 injections every 2 weeks over a course of 8 weeks at dose levels ranging from 25 to 200 μg. The repeat-dose phase was followed by a 14-day observation period to evaluate reversibility of any observed systemic or local toxic effects. The outcomes are summarized in Table S4.

Statistics

Statistical analysis was performed in GraphPad (v.9.5.1). Log rank or ANOVA followed by Tukey's multiple comparisons test were used to determine statistical significance. Values were considered significant with a p value <0.05.

DATA AND CODE AVAILABILITY

All experimental data included in this study are available from the corresponding authors.

ACKNOWLEDGMENTS

We gratefully acknowledge Andrew Elia and Rhoda Law of the Centre for Integrative Immune Analysis (UHN) for histology and imaging, and the NIH Tetramer Core Facility (NIH contract 75N93020D00005 and RRID:SCR_026557) for provision of gp33 and gp34 monomers. This work was supported by Providence Therapeutics Holdings Inc and the We Love You Connie Foundation. Graphical abstract created in BioRender. Krishnan, R. (2026) https://BioRender.com/vqulspw.

AUTHOR CONTRIBUTIONS

M.J.G., D.G.M., J.L., P.S.O., and N.M.O. conceptualized the study; Y.K., K.O., L.T.O., and R.K.G.P. designed and produced ionizable lipids and LNPs; M.J.G., D.G.M., and H.M. performed experiments. All authors provided revisions and approved the final manuscript.

DECLARATION OF INTERESTS

M.J.G., J.L., H.M., .Y.K., K.O., L.T.O., C.D., J.A.S., R.K.G.P., E.G.M., and N.M.O. are employees of and hold stock options of Providence Therapeutics Holdings Inc. E.G.M. and N.M.O. holds stocks and P.S.O. holds stock options and serves on the SAB and receives consulting fees from Providence Therapeutics Holdings Inc.

SUPPLEMENTAL INFORMATION

Supplemental information can be found online at https://doi.org/10.1016/j.omtn.2026.103076.

REFERENCES

1. Polack, F.P., Thomas, S.J., Kitchin, N., Absalon, J., Gurtman, A., Lockhart, S., Perez, J.L., Pérez Marc, G., Moreira, E.D., Zerbini, C., et al. (2020). Safety and Efficacy of the BNT162b2 mRNA Covid-19 Vaccine. N. Engl. J. Med. *383*, 2603–2615. https://doi.org/10.1056/NEJMoa2034577.
2. Baden, L.R., El Sahly, H.M., Essink, B., Kotloff, K., Frey, S., Novak, R., Diemert, D., Spector, S.A., Rouphael, N., Creech, C.B., et al. (2021). Efficacy and Safety of the mRNA-1273 SARS-CoV-2 Vaccine. N. Engl. J. Med. *384*, 403–416. https://doi.org/10.1056/NEJMoa2035389.
3. Nelde, A., Rammensee, H.G., and Walz, J.S. (2021). The Peptide Vaccine of the Future. Mol. Cell. Proteomics *20*, 100022. https://doi.org/10.1074/mcp.R120.002309.
4. Hilf, N., Kuttruff-Coqui, S., Frenzel, K., Bukur, V., Stevanović, S., Gouttefangeas, C., Platten, M., Tabatabai, G., Dutoit, V., van der Burg, S.H., et al. (2019). Actively personalized vaccination trial for newly diagnosed glioblastoma. Nature *565*, 240–245. https://doi.org/10.1038/s41586-018-0810-y.
5. Rojas, L.A., Sethna, Z., Soares, K.C., Olcese, C., Pang, N., Patterson, E., Lihm, J., Ceglia, N., Guasp, P., Chu, A., et al. (2023). Personalized RNA neoantigen vaccines stimulate T cells in pancreatic cancer. Nature *618*, 144–150. https://doi.org/10.1038/s41586-023-06063-y.
6. Awad, M.M., Govindan, R., Balogh, K.N., Spigel, D.R., Garon, E.B., Bushway, M.E., Poran, A., Sheen, J.H., Kohler, V., Esaulova, E., et al. (2022). Personalized neoantigen vaccine NEO-PV-01 with chemotherapy and anti-PD-1 as first-line treatment for non-squamous non-small cell lung cancer. Cancer Cell *40*, 1010–1026.e11. https://doi.org/10.1016/j.ccell.2022.08.003.
7. Disis, M.L.N., Guthrie, K.A., Liu, Y., Coveler, A.L., Higgins, D.M., Childs, J.S., Dang, Y., and Salazar, L.G. (2023). Safety and Outcomes of a Plasmid DNA Vaccine Encoding the ERBB2 Intracellular Domain in Patients With Advanced-Stage ERBB2-Positive Breast Cancer: A Phase 1 Nonrandomized Clinical Trial. JAMA Oncol. *9*, 71–78. https://doi.org/10.1001/jamaoncol.2022.5143.
8. Nichols, W.W., Ledwith, B.J., Manam, S.V., and Troilo, P.J. (1995). Potential DNA vaccine integration into host cell genome. Ann. N. Y. Acad. Sci. *772*, 30–39. https://doi.org/10.1111/j.1749-6632.1995.tb44729.x.
9. Ben-Akiva, E., Chapman, A., Mao, T., and Irvine, D.J. (2025). Linking vaccine adjuvant mechanisms of action to function. Sci. Immunol. *10*, eado5937. https://doi.org/10.1126/sciimmunol.ado5937.
10. Braun, D.A., Moranzoni, G., Chea, V., McGregor, B.A., Blass, E., Tu, C.R., Vanasse, A.P., Forman, C., Forman, J., Afeyan, A.B., et al. (2025). A neoantigen vaccine generates antitumour immunity in renal cell carcinoma. Nature *639*, 474–482. https://doi.org/10.1038/s41586-024-08507-5.
11. Gilleron, J., Querbes, W., Zeigerer, A., Borodovsky, A., Marsico, G., Schubert, U., Manygoats, K., Seifert, S., Andree, C., Stöter, M., et al. (2013). Image-based analysis of lipid nanoparticle-mediated siRNA delivery, intracellular trafficking and endosomal escape. Nat. Biotechnol. *31*, 638–646. https://doi.org/10.1038/nbt.2612.
12. Pardi, N., Hogan, M.J., Porter, F.W., and Weissman, D. (2018). mRNA vaccines - a new era in vaccinology. Nat. Rev. Drug Discov. *17*, 261–279. https://doi.org/10.1038/nrd.2017.243.
13. Kulkarni, J.A., Witzigmann, D., Chen, S., Cullis, P.R., and van der Meel, R. (2019). Lipid Nanoparticle Technology for Clinical Translation of siRNA Therapeutics. Acc. Chem. Res. *52*, 2435–2444. https://doi.org/10.1021/acs.accounts.9b00368.
14. Akinc, A., Querbes, W., De, S., Qin, J., Frank-Kamenetsky, M., Jayaprakash, K.N., Jayaraman, M., Rajeev, K.G., Cantley, W.L., Dorkin, J.R., et al. (2010). Targeted delivery of RNAi therapeutics with endogenous and exogenous ligand-based mechanisms. Mol. Ther. *18*, 1357–1364. https://doi.org/10.1038/mt.2010.85.
15. Chen, J., Ye, Z., Huang, C., Qiu, M., Song, D., Li, Y., and Xu, Q. (2022). Lipid nanoparticle-mediated lymph node-targeting delivery of mRNA cancer vaccine elicits robust CD8(+) T cell response. Proc. Natl. Acad. Sci. USA *119*, e2207841119. https://doi.org/10.1073/pnas.2207841119.

16. Luo, Z., Lin, Y., Meng, Y., Li, M., Ren, H., Shi, H., Cheng, Q., and Wei, T. (2024). Spleen-Targeted mRNA Vaccine Doped with Manganese Adjuvant for Robust Anticancer Immunity In Vivo. ACS Nano *18*, 30701–30715. https://doi.org/10.1021/acsnano.4c09902.

17. Sittplangkoon, C., Alameh, M.G., Weissman, D., Lin, P.J.C., Tam, Y.K., Prompetchara, E., and Palaga, T. (2022). mRNA vaccine with unmodified uridine induces robust type I interferon-dependent anti-tumor immunity in a melanoma model. Front. Immunol. *13*, 983000. https://doi.org/10.3389/fimmu.2022.983000.

18. Tahtinen, S., Tong, A.J., Himmels, P., Oh, J., Paler-Martinez, A., Kim, L., Wichner, S., Oei, Y., McCarron, M.J., Freund, E.C., et al. (2022). IL-1 and IL-1ra are key regulators of the inflammatory response to RNA vaccines. Nat. Immunol. *23*, 532–542. https://doi.org/10.1038/s41590-022-01160-y.

19. Alameh, M.G., Tombácz, I., Bettini, E., Lederer, K., Sittplangkoon, C., Wilmore, J.R., Gaudette, B.T., Soliman, O.Y., Pine, M., Hicks, P., et al. (2021). Lipid nanoparticles enhance the efficacy of mRNA and protein subunit vaccines by inducing robust T follicular helper cell and humoral responses. Immunity *54*, 2877–2892.e7. https://doi.org/10.1016/j.immuni.2021.11.001.

20. Li, C., Lee, A., Grigoryan, L., Arunachalam, P.S., Scott, M.K.D., Trisal, M., Wimmers, F., Sanyal, M., Weidenbacher, P.A., Feng, Y., et al. (2022). Mechanisms of innate and adaptive immunity to the Pfizer-BioNTech BNT162b2 vaccine. Nat. Immunol. *23*, 543–555. https://doi.org/10.1038/s41590-022-01163-9.

21. Bevers, S., Kooijmans, S.A.A., Van de Velde, E., Evers, M.J.W., Seghers, S., Gitz-Francois, J.J.J.M., van Kronenburg, N.C.H., Fens, M.H.A.M., Mastrobattista, E., Hassler, L., et al. (2022). mRNA-LNP vaccines tuned for systemic immunization induce strong antitumor immunity by engaging splenic immune cells. Mol. Ther. *30*, 3078–3094. https://doi.org/10.1016/j.ymthe.2022.07.007.

22. Li, B., Jiang, A.Y., Raji, I., Atyeo, C., Raimondo, T.M., Gordon, A.G.R., Rhym, L.H., Samad, T., MacIsaac, C., Witten, J., et al. (2025). Enhancing the immunogenicity of lipid-nanoparticle mRNA vaccines by adjuvanting the ionizable lipid and the mRNA. Nat. Biomed. Eng. *9*, 167–184. https://doi.org/10.1038/s41551-023-01082-6.

23. Miao, L., Li, L., Huang, Y., Delcassian, D., Chahal, J., Han, J., Shi, Y., Sadtler, K., Gao, W., Lin, J., et al. (2019). Delivery of mRNA vaccines with heterocyclic lipids increases anti-tumor efficacy by STING-mediated immune cell activation. Nat. Biotechnol. *37*, 1174–1185. https://doi.org/10.1038/s41587-019-0247-3.

24. Rehwinkel, J.A.-O.X., and Gack, M.A.-O. (2020). RIG-I-like receptors: their regulation and roles in RNA sensing. Nat. Rev. Immunol. *20*, 537–551.

25. Karikó, K., Muramatsu, H., Welsh, F.A., Ludwig, J., Kato, H., Akira, S., and Weissman, D. (2008). Incorporation of pseudouridine into mRNA yields superior nonimmunogenic vector with increased translational capacity and biological stability. Mol. Ther. *16*, 1833–1840. https://doi.org/10.1038/mt.2008.200.

26. Holm, C.K., Rahbek, S.H., Gad, H.H., Bak, R.O., Jakobsen, M.R., Jiang, Z., Hansen, A.L., Jensen, S.K., Sun, C., Thomsen, M.K., et al. (2016). Influenza A virus targets a cGAS-independent STING pathway that controls enveloped RNA viruses. Nat. Commun. *7*, 10680. https://doi.org/10.1038/ncomms10680.

27. Baharom, F., Ramirez-Valdez, R.A., Khalilnezhad, A., Khalilnezhad, S., Dillon, M., Hermans, D., Fussell, S., Tobin, K.K.S., Dutertre, C.A., Lynn, G.M., et al. (2022). Systemic vaccination induces CD8(+) T cells and remodels the tumor microenvironment. Cell *185*, 4317–4332.e15. https://doi.org/10.1016/j.cell.2022.10.006.

28. Sanlorenzo, M., Novoszel, P., Vujic, I., Gastaldi, T., Hammer, M., Fari, O., De Sa Fernandes, C., Landau, A.D., Göcen-Oguz, B.V., Holcmann, M., et al. (2025). Systemic IFN-I combined with topical TLR7/8 agonists promotes distant tumor suppression by c-Jun-dependent IL-12 expression in dendritic cells. Nat. Cancer *6*, 175–193. https://doi.org/10.1038/s43018-024-00889-9.

29. Sikora, A.G., Jaffarzad, N., Hailemichael, Y., Gelbard, A., Stonier, S.W., Schluns, K.S., Frasca, L., Lou, Y., Liu, C., Andersson, H.A., et al. (2009). IFN-alpha enhances peptide vaccine-induced CD8+ T cell numbers, effector function, and antitumor activity. J. Immunol. *182*, 7398–7407. https://doi.org/10.4049/jimmunol.0802982.

30. Chen, W., Teo, J.M.N., Yau, S.W., Wong, M.Y.M., Lok, C.N., Che, C.M., Javed, A., Huang, Y., Ma, S., and Ling, G.S. (2022). Chronic type I interferon signaling promotes lipid-peroxidation-driven terminal CD8(+) T cell exhaustion and curtails anti-PD-1 efficacy. Cell Rep. *41*, 111647. https://doi.org/10.1016/j.celrep.2022.111647.

31. Sahin, U., Oehm, P., Derhovanessian, E., Jabulowsky, R.A., Vormehr, M., Gold, M., Maurus, D., Schwarck-Kokarakis, D., Kuhn, A.N., Omokoko, T., et al. (2020). An RNA vaccine drives immunity in checkpoint-inhibitor-treated melanoma. Nature *585*, 107–112. https://doi.org/10.1038/s41586-020-2537-9.

32. Kübler, H., Scheel, B., Gnad-Vogt, U., Miller, K., Schultze-Seemann, W., Vom Dorp, F., Parmiani, G., Hampel, C., Wedel, S., Trojan, L., et al. (2015). Self-adjuvanted mRNA vaccination in advanced prostate cancer patients: a first-in-man phase I/IIa study. J. Immunother. Cancer *3*, 26. https://doi.org/10.1186/s40425-015-0068-y.

33. Sebastian, M., Schröder, A., Scheel, B., Hong, H.S., Muth, A., von Boehmer, L., Zippelius, A., Mayer, F., Reck, M., Atanackovic, D., et al. (2019). A phase I/IIa study of the mRNA-based cancer immunotherapy CV9201 in patients with stage IIIB/IV non-small cell lung cancer. Cancer Immunol. Immunother. *68*, 799–812. https://doi.org/10.1007/s00262-019-02315-x.

34. Giles, J.R., Globig, A.M., Kaech, S.M., and Wherry, E.J. (2023). CD8(+) T cells in the cancer-immunity cycle. Immunity *56*, 2231–2253. https://doi.org/10.1016/j.immuni.2023.09.005.

35. Gairin, J.E., Mazarguil, H., Hudrisier, D., and Oldstone, M.B. (1995). Optimal lymphocytic choriomeningitis virus sequences restricted by H-2Db major histocompatibility complex class I molecules and presented to cytotoxic T lymphocytes. J. Virol. *69*, 2297–2305. https://doi.org/10.1128/JVI.69.4.2297-2305.1995.

36. Hudrisier, D., Oldstone, M.B., and Gairin, J.E. (1997). The signal sequence of lymphocytic choriomeningitis virus contains an immunodominant cytotoxic T cell epitope that is restricted by both H-2D(b) and H-2K(b) molecules. Virology *234*, 62–73. https://doi.org/10.1006/viro.1997.8627.

37. Oxenius, A., Bachmann, M.F., Zinkernagel, R.M., and Hengartner, H. (1998). Virus-specific MHC-class II-restricted TCR-transgenic mice: effects on humoral and cellular immune responses after viral infection. Eur. J. Immunol. *28*, 390–400. https://doi.org/10.1002/(SICI)1521-4141(199801)28:01<390::AID-IMMU390>3.0.CO;2-O.

38. Palmer, C.D., Rappaport, A.R., Davis, M.J., Hart, M.G., Scallan, C.D., Hong, S.J., Gitlin, L., Kraemer, L.D., Kounlavouth, S., Yang, A., et al. (2022). Individualized, heterologous chimpanzee adenovirus and self-amplifying mRNA neoantigen vaccine for advanced metastatic solid tumors: phase 1 trial interim results. Nat. Med. *28*, 1619–1629. https://doi.org/10.1038/s41591-022-01937-6.

39. Ohashi, P.S., Oehen, S., Buerki, K., Pircher, H., Ohashi, C.T., Odermatt, B., Malissen, B., Zinkernagel, R.M., and Hengartner, H. (1991). Ablation of "tolerance" and induction of diabetes by virus infection in viral antigen transgenic mice. Cell *65*, 305–317. https://doi.org/10.1016/0092-8674(91)90164-t.

40. Lang, K.S., Recher, M., Junt, T., Navarini, A.A., Harris, N.L., Freigang, S., Odermatt, B., Conrad, C., Ittner, L.M., Bauer, S., et al. (2005). Toll-like receptor engagement converts T-cell autoreactivity into overt autoimmune disease. Nat. Med. *11*, 138–145. https://doi.org/10.1038/nm1176.

41. Lin, A.C.C., Dissanayake, D., Dhanji, S., Elford, A.R., and Ohashi, P.S. (2011). Different toll-like receptor stimuli have a profound impact on cytokines required to break tolerance and induce autoimmunity. PLoS One *6*, e23940. https://doi.org/10.1371/journal.pone.0023940.

42. Guo, M., Abd-Rabbo, D., Bertol, B.C., Carew, M., Lukhele, S., Snell, L.M., Xu, W., Boukhaled, G.M., Elsaesser, H., Halaby, M.J., et al. (2023). Molecular, metabolic, and functional CD4 T cell paralysis in the lymph node impedes tumor control. Cell Rep. *42*, 113047. https://doi.org/10.1016/j.celrep.2023.113047.

43. Hurez, V., Daniel, B.J., Sun, L., Liu, A.J., Ludwig, S.M., Kious, M.J., Thibodeaux, S.R., Pandeswara, S., Murthy, K., Livi, C.B., et al. (2012). Mitigating age-related immune dysfunction heightens the efficacy of tumor immunotherapy in aged mice. Cancer Res. *72*, 2089–2099. https://doi.org/10.1158/0008-5472.CAN-11-3019.

44. Dai, W., Xing, M., Sun, L., Lv, L., Wang, X., Wang, Y., Pang, X., Guo, Y., Ren, J., and Zhou, D. (2024). Lipid nanoparticles as adjuvant of norovirus VLP vaccine augment cellular and humoral immune responses in a TLR9- and type I IFN-dependent pathway. J. Virol. *98*, e0169924. https://doi.org/10.1128/jvi.01699-24.

45. Dowell, W., Dearborn, J., Languon, S., Miller, Z., Kirch, T., Paige, S., Garvin, O., Kjendal, L., Harby, E., Zuchowski, A.B., et al. (2024). Distinct Inflammatory Programs Underlie the Intramuscular Lipid Nanoparticle Response. ACS Nano *18*, 33058–33072. https://doi.org/10.1021/acsnano.4c08490.

46. Kim, S., Jeon, J.H., Kim, M., Lee, Y., Hwang, Y.H., Park, M., Li, C.H., Lee, T., Lee, J.A., Kim, Y.M., et al. (2024). Innate immune responses against mRNA vaccine promote cellular immunity through IFN-beta at the injection site. Nat. Commun. *15*, 7226. https://doi.org/10.1038/s41467-024-51411-9.

47. Sheehan, K.C.F., Lai, K.S., Dunn, G.P., Bruce, A.T., Diamond, M.S., Heutel, J.D., Dungo-Arthur, C., Carrero, J.A., White, J.M., Hertzog, P.J., and Schreiber, R.D. (2006). Blocking monoclonal antibodies specific for mouse IFN-alpha/beta receptor subunit 1 (IFNAR-1) from mice immunized by in vivo hydrodynamic transfection. J. Interferon Cytokine Res. *26*, 804–819. https://doi.org/10.1089/jir.2006.26.804.

48. Wolfl, M., Kuball, J., Ho, W.Y., Nguyen, H., Manley, T.J., Bleakley, M., and Greenberg, P.D. (2007). Activation-induced expression of CD137 permits detection, isolation, and expansion of the full repertoire of CD8+ T cells responding to antigen without requiring knowledge of epitope specificities. Blood *110*, 201–210. https://doi.org/10.1182/blood-2006-11-056168.

49. Oyama, R., Ishigame, H., Tanaka, H., Tateshita, N., Itazawa, M., Imai, R., Nishiumi, N., Kishikawa, J.I., Kato, T., Anindita, J., et al. (2023). An Ionizable Lipid Material with a Vitamin E Scaffold as an mRNA Vaccine Platform for Efficient Cytotoxic T Cell Responses. ACS Nano *17*, 18758–18774. https://doi.org/10.1021/acsnano.3c02251.

50. Marks, A., Siu, S., Bianchini, F., Wang, C., Lakshmi, A., Phelan, M., Zhu, A., Moon, C., Morla-Folch, J., Teunissen, A.J.P., et al. (2026). mRNA vaccine immunity is enhanced by hepatocyte detargeting and not dependent on dendritic cell expression. Nat. Biotechnol. https://doi.org/10.1038/s41587-026-03099-z.

51. Jo, S., Li, L., Thakur, C., Telfer, K.A., Sultan, H., Ohara, R.A., He, M., Nam, G., Chen, J., Ou, F., et al. (2026). mRNA vaccines engage unconventional pathways in CD8(+) T cell priming. Nature *654*, 485–494. https://doi.org/10.1038/s41586-026-10353-6.

52. Dhatchinamoorthy, K., Colbert, J.D., and Rock, K.L. (2021). Cancer Immune Evasion Through Loss of MHC Class I Antigen Presentation. Front. Immunol. *12*, 636568. https://doi.org/10.3389/fimmu.2021.636568.

53. Melero, I., Shuford, W.W., Newby, S.A., Aruffo, A., Ledbetter, J.A., Hellström, K.E., Mittler, R.S., and Chen, L. (1997). Monoclonal antibodies against the 4-1BB T-cell activation molecule eradicate established tumors. Nat. Med. *3*, 682–685. https://doi.org/10.1038/nm0697-682.

54. Pichler, A.C., Carrié, N., Cuisinier, M., Ghazali, S., Voisin, A., Axisa, P.P., Tosolini, M., Mazzotti, C., Golec, D.P., Maheo, S., et al. (2023). TCR-independent CD137 (4-1BB) signaling promotes CD8(+)-exhausted T cell proliferation and terminal differentiation. Immunity *56*, 1631–1648.e10. https://doi.org/10.1016/j.immuni.2023.06.007.

55. Gungabeesoon, J., Gort-Freitas, N.A., Kiss, M., Bolli, E., Messemaker, M., Siwicki, M., Hicham, M., Bill, R., Koch, P., Cianciaruso, C., et al. (2023). A neutrophil response linked to tumor control in immunotherapy. Cell *186*, 1448–1464.e20. https://doi.org/10.1016/j.cell.2023.02.032.

56. Teijeira, Á., Garasa, S., Gato, M., Alfaro, C., Migueliz, I., Cirella, A., de Andrea, C., Ochoa, M.C., Otano, I., Etxeberria, I., et al. (2020). CXCR1 and CXCR2 Chemokine Receptor Agonists Produced by Tumors Induce Neutrophil Extracellular Traps that Interfere with Immune Cytotoxicity. Immunity *52*, 856–871.e8. https://doi.org/10.1016/j.immuni.2020.03.001.

57. Albrengues, J., Shields, M.A., Ng, D., Park, C.G., Ambrico, A., Poindexter, M.E., Upadhyay, P., Uyeminami, D.L., Pommier, A., Küttner, V., et al. (2018). Neutrophil extracellular traps produced during inflammation awaken dormant cancer cells in mice. Science *361*, eaao4227. https://doi.org/10.1126/science.aao4227.

58. Li, Y., Wu, S., Zhao, Y., Dinh, T., Jiang, D., Selfridge, J.E., Myers, G., Wang, Y., Zhao, X., Tomchuck, S., et al. (2024). Neutrophil extracellular traps induced by chemotherapy inhibit tumor growth in murine models of colorectal cancer. J. Clin. Investig. *134*, e175031. https://doi.org/10.1172/JCI175031.

59. Eruslanov, E.B., Bhojnagarwala, P.S., Quatromoni, J.G., Stephen, T.L., Ranganathan, A., Deshpande, C., Akimova, T., Vachani, A., Litzky, L., Hancock, W.W., et al. (2014). Tumor-associated neutrophils stimulate T cell responses in early-stage human lung cancer. J. Clin. Investig. *124*, 5466–5480. https://doi.org/10.1172/JCI77053.

60. Tang, Z., Hu, J., Li, X.C., Wang, W., Zhang, H.Y., Guo, Y.Y., Shuai, X., Chu, Q., Xie, C., Lin, D., and Zhong, B. (2025). A subset of neutrophils activates anti-tumor immunity and inhibits non-small-cell lung cancer progression. Dev. Cell *60*, 379–395.e8. https://doi.org/10.1016/j.devcel.2024.10.010.

61. Benguigui, M., Cooper, T.J., Kalkar, P., Schif-Zuck, S., Halaban, R., Bacchiocchi, A., Kamer, I., Deo, A., Manobla, B., Menachem, R., et al. (2024). Interferon-stimulated neutrophils as a predictor of immunotherapy response. Cancer Cell *42*, 253–265.e12. https://doi.org/10.1016/j.ccell.2023.12.005.

62. Hirschhorn, D., Budhu, S., Kraehenbuehl, L., Gigoux, M., Schröder, D., Chow, A., Ricca, J.M., Gasmi, B., De Henau, O., Mangarin, L.M.B., et al. (2023). T cell immunotherapies engage neutrophils to eliminate tumor antigen escape variants. Cell *186*, 1432–1447.e17. https://doi.org/10.1016/j.cell.2023.03.007.

63. Wu, W., Oliveira, L.T., Jain, A., Karpov, Y., Olsen, K., Wu, Y., and Gopalakrishna Panicker, R.K. (2025). Process development of tangential flow filtration and sterile filtration for manufacturing of mRNA-lipid nanoparticles: A study on membrane performance and filtration modeling. Int. J. Pharm. *675*, 125520. https://doi.org/10.1016/j.ijpharm.2025.125520.

OMTN, Volume 37

Supplemental information

Novel lipid nanoparticle in a mRNA cancer vaccine drives tumor control via type I IFNs and effector CD8^{+} T cells

Matthew J. Gold, Douglas G. Millar, Jun Liu, Haritha Menon, Yury Karpov, Kirstin Olsen, Liliam Teixeira Oliveira, Charlotte Dubé, Charles W. Tran, Alisha R. Elford, Jordan A. Schwartz, Rajesh Krishnan Gopalakrishna Panicker, Eric G. Marcusson, Pamela S. Ohashi, and Natalia Martin Orozco

Table S1 – Summary of physiochemical characteristics of mRNA-LNP formulations (mean±SEM)

Formulation	INTENT-1.1	INTENT-2.1	ALC-0315	SM-102
Size (nm)	79 ± 12	71±4	140	80±2
PDI	0.23±0.09	0.09±0.02	0.09	0.06±0.01
mRNA encapsulation (%)	91±3	96±0.3	79	94±0.8
Zeta Potential (mV)	5±0.6	4±3	-10.3	-1±7
mRNA integrity (%)	96±1	97±1	99	96±2

Table S2 – Flow Cytometry Antibodies

Antibody	Vendor	Clone
Fixable viability dye e506	Thermo Fisher	n/a
CD16/32	BD Pharmingen	2.4G2
CD4	Thermo Fisher	GK1.5
CD8a	Thermo Fisher	53-6.7
CD11b	Thermo Fisher	M1/70
CD11c	Thermo Fisher	N418
CD45	Thermo Fisher	30-F11
CD80	Thermo Fisher	16-10A1
CD137/4-1BB	Thermo Fisher	17B5
CD279/PD-1	Thermo Fisher	J43
TCRβ	BD Pharmingen	H57-597
MHC-II	Thermo Fisher	AF6-120.1
SiglecH	Thermo Fisher	eBio440c
Ly6C	Thermo Fisher	HK1.4
Ly6G	Thermo Fisher	1A8
iNOS	Thermo Fisher	CXNFT
IFNγ	Thermo Fisher	XMG1.2

TNFα	Thermo Fisher	MPX-XT22

Table S3 – qRT-PCR Primers

**Gene**	**Forward**	**Reverse**
Actb	GGCTGTATTCCCCTCCATCG	CCAGTTGGTAACAATGCCATGT
Ccl2	CTCGGACTGTGATGCCTTAAT	TGGATCCACACCTTGCATTTA
Ccl3	TTCTCTGTACCATGACACTCTGC	CGTGGAATCTTCCGGCTGTAG
Cxcl1	GCTGGGATTCACCTCAAGAA	TGGCTATGACTTCGGTTTGG
Cxcl2	AGTGAACTGCGCTGTCAATGC	AGGCAAACTTTTTGACCGCC
Cxcl9	GGAGTTCGAGGAACCCTAGTG	GGGATTTGTAGTGGATCGTGC
Cxcl10	CCAAGTGCTGCCGTCATTTTC	GGCTCGCAGGGATGATTTCAA
Il1a	GCTTGAGTCGGCAAAGAAATC	GAGAGATGGTCAATGGCAGAA
Il1b	AAGGAGAACCAAGCAACGACAAAA	TGGGGAACTCTGCAGACTCAAACT
Il1rn	TTGTGCCAAGTCTGGAGATG	CTCAGAGCGGATGAAGGTAAAG
Il6	TAG TCC TTC CTA CCC CAA TTT CC	TTG GTC CTT AGC CAC TCC TTC
Ifna-pan	CCTGAGAAAGAAGAAACACAGCC	GGCTCTCCAGACTTCTGCTCTG
	CCTGAGAGAGAAGAAACACAGCC	GGCTCTCCAGATTTCTGCTCTG
Ifnb1	GCACTGGGTGGAATGAGACT	AGTGGAGAGCAGTTGAGGACA
Ifng	CTCTTCCTCATGGCTGTTTCT	TTCTTCCACATCTATGCCACTT
Mx1	CCAACTGGAATCCTCCTGGAA	GCCGCACCTTCTCCTCATAG

Table S4 – Outcomes for Repeat-Dose Phase of the INTENT-2.11 Tolerability Study in Rats

**Parameter**	**Time of Assessment**	**Outcomes**	
		**Dosing Period**	**Recovery Period**
**Mortality**	Daily	No mortality observed in any animal group.	No mortality observed in any animal group.
**Clinical observations**	Prior and daily for 3 days post each administration, otherwise twice weekly until the end of dosing/recovery period.	No systemic clinical signs were observed.	No systemic clinical signs were observed.

Parameter	Time of Assessment	Outcomes	
		Dosing Period	Recovery Period
**Injection Site Observations (Draize Score)**	Daily for 3 days post each administration, otherwise weekly until the end of dosing/recovery period.	Dose-proportionate edema (slight to moderate) associated with abnormal gait (up to 2 days post-dose) occurred after each administration, with a higher severity in females and fully resolving between doses. No erythema, except for few female animals (3/10) at 200 µg dose.	No longer present by the end of the recovery period.
**Body Weight**	Prior and daily for 3 days post each administration, otherwise twice weekly until the end of dosing/recovery period.	Body weight gain over time with a transient decrease (1-2 days) following each administration (males only).	Similar body weight gain between all groups.
**Food Consumption**	Weekly until the end of dosing/recovery period.	Decrease of food intake during the first week following each administration (males and females).	Similar food intake between all groups.
**Hematology** (1)	End of dosing /recovery period.	Marked increases in white blood cell counts (neutrophils and monocytes) observed at all doses (males and females).<br>Slight reduction in reticulocyte counts in males (all doses) and females (100 and 200 µg doses).	Persistent increases in white blood cell counts at higher doses with a lower magnitude indicating an ongoing recovery.
**Clinical Chemistry** (2)	End of dosing /recovery period.	Slight decrease in cholesterol levels (male only) at higher doses.	Return to normal at the end of the recovery.
**Gross Examination** (3)	End of dosing /recovery period.	Enlargement of the popliteal lymph node (1/10 male, 100 µg), dark area at the injection site (1/10 female, 200 µg).	No abnormal findings.

Parameter	Time of Assessment	Outcomes	
		**Dosing Period**	**Recovery Period**
**Organ Weight** [(4)]	End of dosing /recovery period.	No changes.	No changes.
**Histopathology** [(5)]	End of dosing /recovery period.<br><br>*(Performed on control and 200 µg dose group animals; (n=2/sex))*	Injection site: Minimal to marked inflammation of the perimysium and/or the endomysium in all animals (2/2 males and 2/2 females) dosed at 200 µg.<br>Lymph nodes: Increased incidence of mild increased cellularity of the inguinal lymph node noted in animals dosed at 200 µg (1/2 male and 1/2 female) when compared to controls (1/2 male).<br>Liver: Minimal to mild periportal vacuolation of the hepatocytes was noted in all animals (2/2 males and 2/2 females) dosed at 200 µg. The same observation was also noted in a single control female animal at a lower severity (minimal).	Microscopic findings in the liver had resolved by the end of the recovery period. Inflammation at the injection site and vacuolated macrophages in the lymph nodes were still present with a decreased severity, suggesting an ongoing recovery.

(1) Hematology: Red blood cell, hematocrit, hemoglobin, white blood cell, mean corpuscular hemoglobin, morphology of cells, platelet, reticulocyte

(2) Clinical chemistry: A/G ratio, alanine aminotransferase, albumin, alkaline phosphatase, aspartate aminotransferase, bilirubin, calcium, chloride, cholesterol, gamma-glutamyl transferase, creatinine, creatine kinase, globulin, glucose, phosphorus, potassium, sodium, total protein, triglycerides, urea

(3) Gross examination: Adrenals, aorta, brain, cecum, colon, epididymis, esophagus, eyes, femur and marrow, heart, injection sites, kidneys, liver, lungs, axillary/inguinal/mandibular/mesenteric/popliteal lymph nodes, mammary glands, optic nerves, ovaries, pancreas, pituitary, prostate, rectum, salivary glands, sciatic nerve, seminal vesicles, skeletal muscle, skin and subcutis, duodenum, jejunum, ileum, spinal cord (cervical), spleen, sternum and marrow, stomach, testes, thymus, thyroid gland, tongue, trachea, urinary bladder, uterus, vagina

(4) Organ weight: Adrenals, brain, heart, kidneys, liver, lungs, ovaries, pituitary, prostate, spleen, testes, thymus, thyroid gland and parathyroids, uterus

(5) Histopathology: Liver, spleen, biceps femoris, inguinal lymph nodes

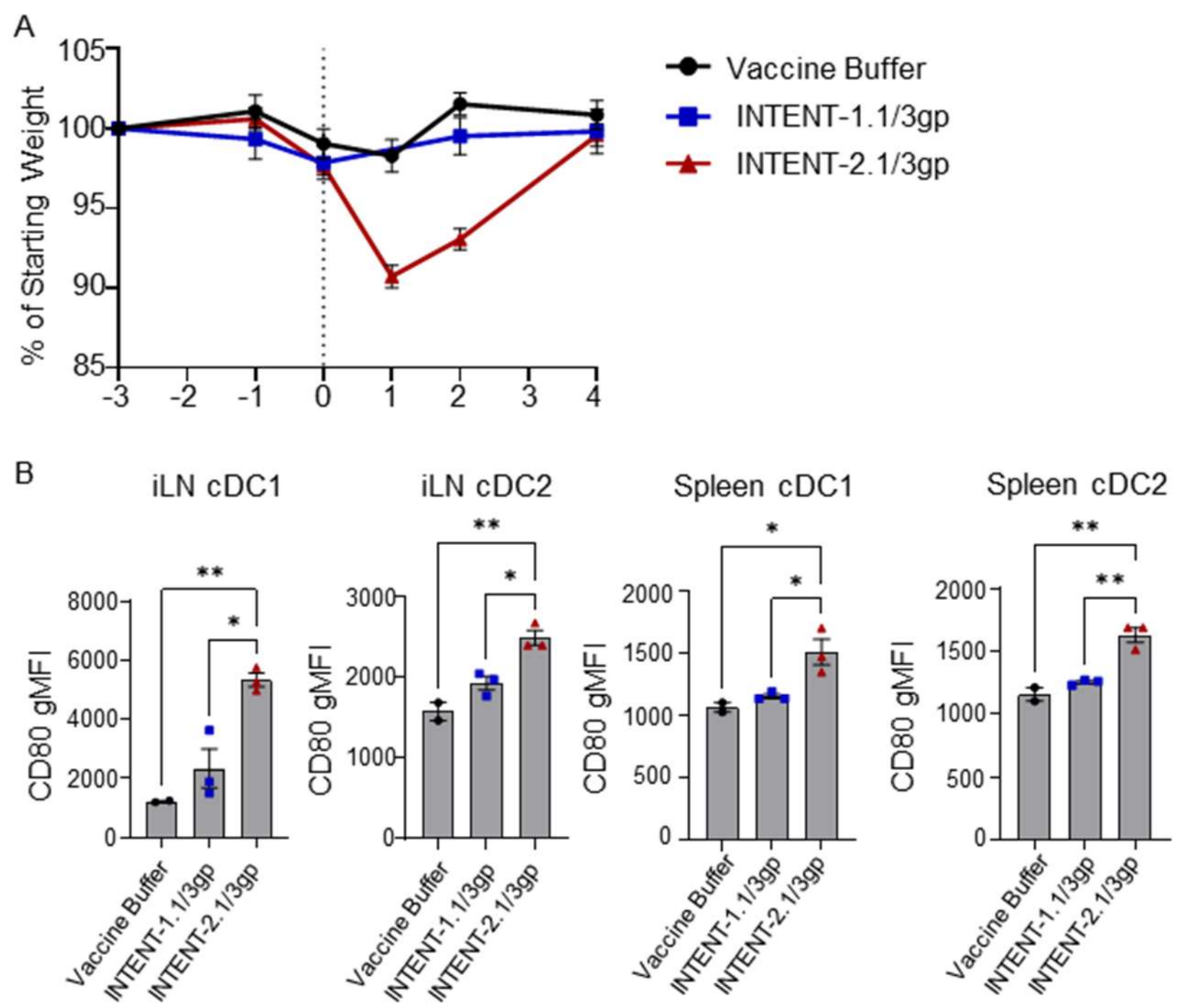

<u>Figure S1 – INTENT-1.1 and INTENT-2.1 Toxicity and APC Activation</u>

(A) Total body mass was measured prior to and following vaccine buffer or LNP administration (day 0) and shown as a percentage of starting weight (n=6-12). (B) Draining inguinal lymph nodes (iLN) and spleens were collected from C57BL/6 mice 24hrs after vaccine buffer or INTENT LNP administration (n=2-3). Dendritic cells were identified as $CD11c^{+}MHC\text{-}II^{+}CD3^{-}CD19^{-}$ and defined as cDC1 or cDC2 based on CD8a and CD11b expression, respectively. Statistics in (B) were performed by one-way ANOVA with Tukey's multiple comparison test, *$p<0.05$, **$p<0.01$.

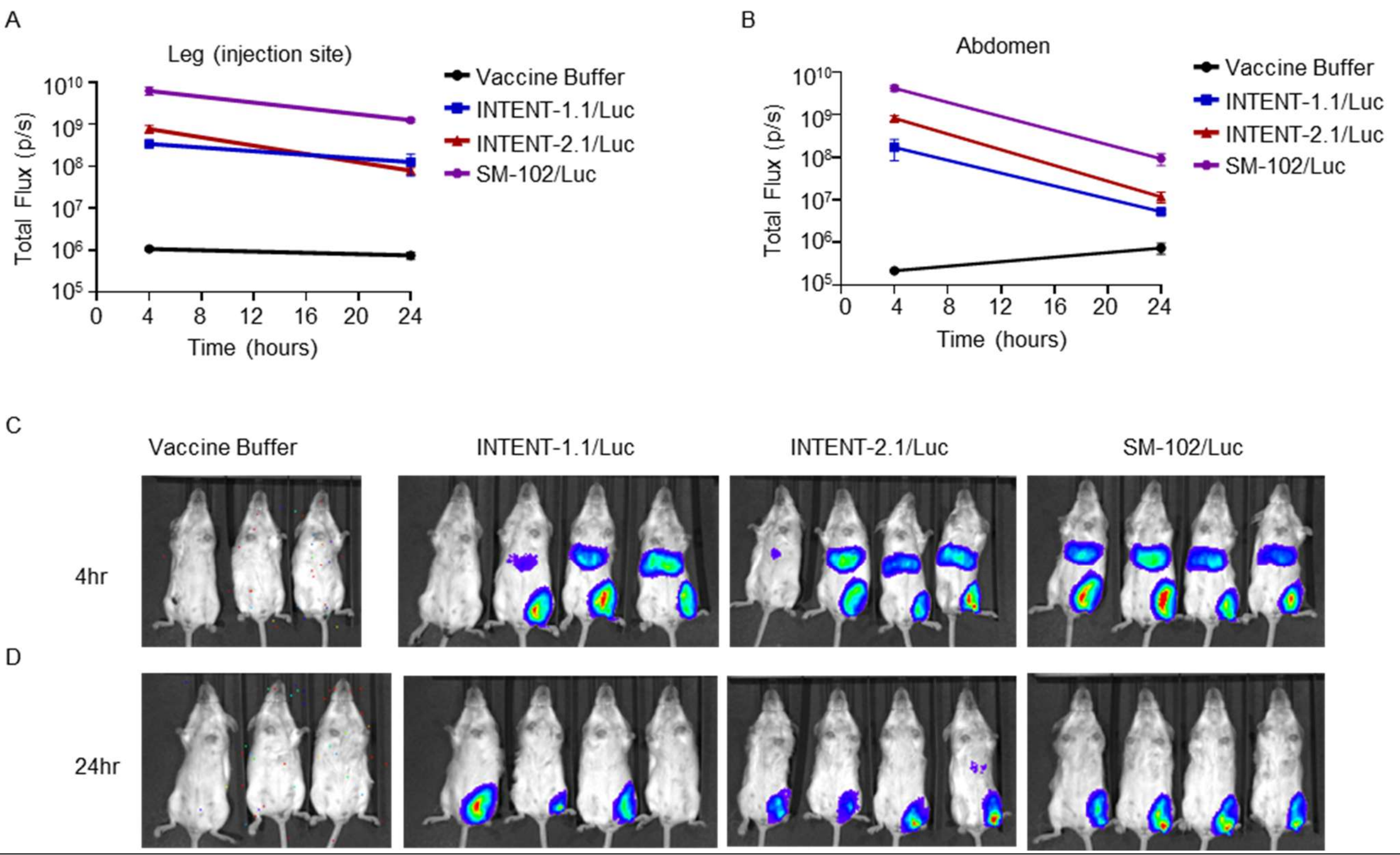

Figure S2 – *In vivo* biodistribution

BALBc mice were administered the indicated LNPs and 4hr or 24hr post-injection luciferin substrate was delivered prior to imaging. (A) Luminescence in the injection site area or (B) abdomen was quantified. Representative images (C) 4hrs and (D) 24hrs after LNP administration.

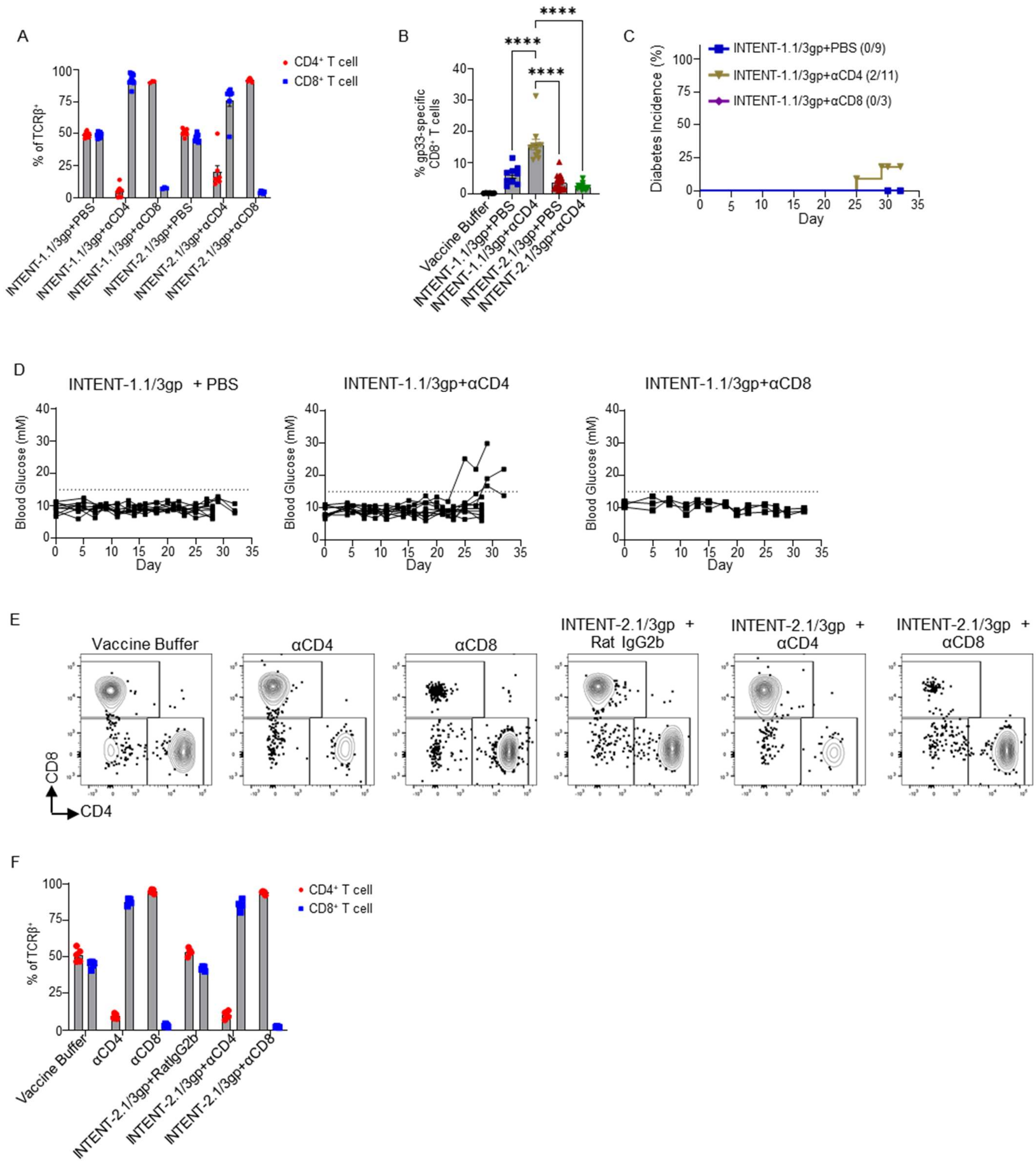

Figure S3 – CD4⁺ and CD8⁺ T cell depletion in RIPgp and MC38gp models

(A) Peripheral blood was collected on day 6-8 from RIPgp animals treated as in Figure 3A and depletion of $CD4^+$ and $CD8^+$ T cells was confirmed by flow cytometry, gated on $TCR\beta^+CD45^+$ cells . (B) Frequency of gp33-specific $CD8^+$ T cells in the blood from RIPgp animals treated with the indicated LNPs. (C) Diabetes incidence and (D) individual animal blood glucose from RIPgp mice. (E) Peripheral blood was collected on day 9 from MC38gp tumor bearing animals as treated in Figure 4E-G and shown gated on $TCR\beta^+CD45^+$ cells from the indicated treatment groups and summarized in (F). Statistics in (B) was performed by one-way ANOVA and Tukey's multiple comparisons test, ****$p<0.0001$.

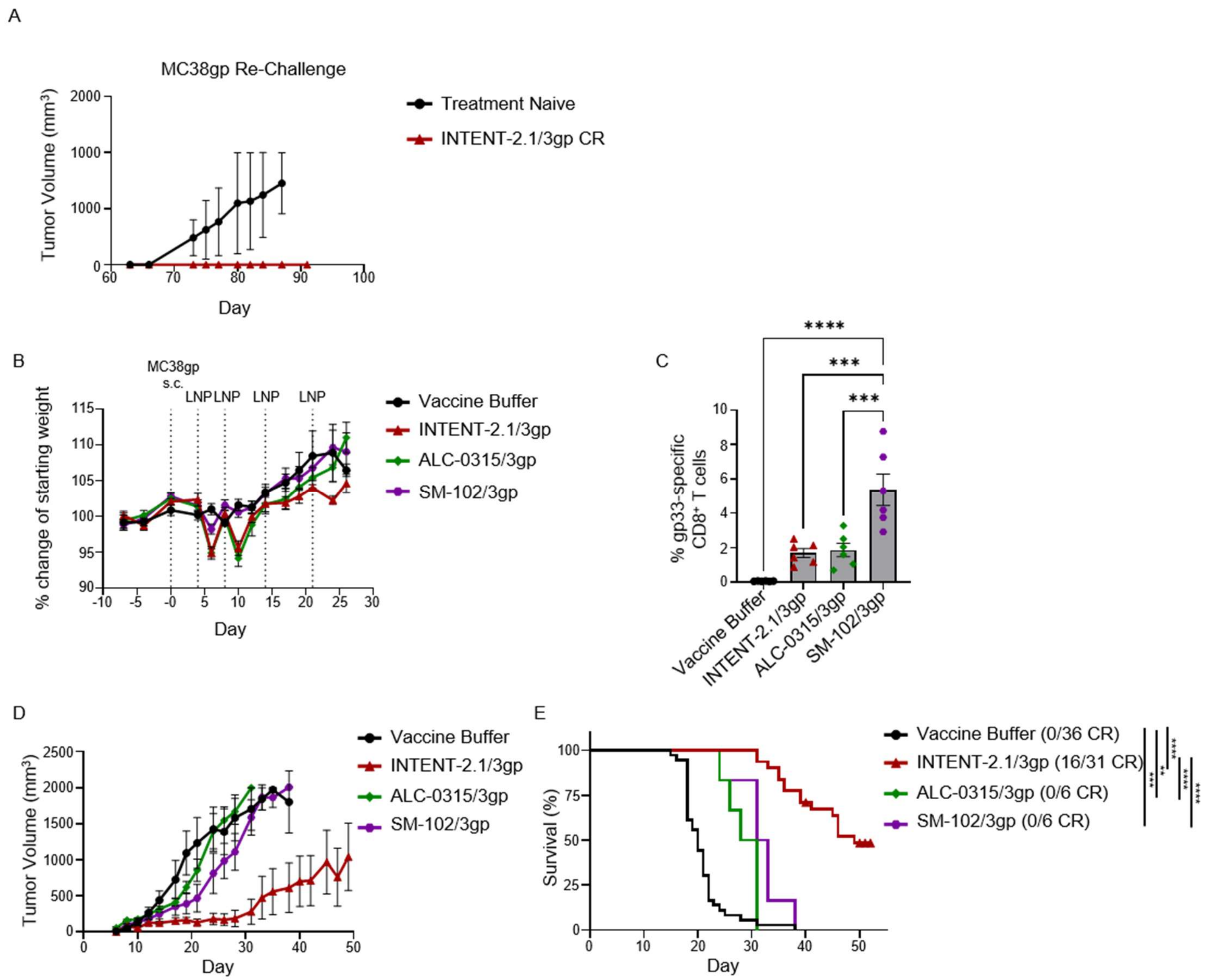

<u>Figure S4 – INTENT-2.1/3gp provides immune memory to tumor re-challenge and superior tumor control to benchmark LNPs</u>

(A) INTENT-2.1/3gp treated C57BL/6 mice that successfully cleared established MC38gp tumor (n=3) or naïve controls (n=2) were re-challenged with MC38gp tumor cells (1x10^6 cells s.c.) and tumor growth was monitored. (B) Percent change in body weight from C57BL/6 mice administered MC38gp cells (day 0) and the indicated LNPs (days 4, 8, 14 & 21). (C) Frequency of gp33-specific $CD8^+$ T cells in the blood on day 11, shown as frequency of total $CD8^+$ T cells. (D) Tumor volume and (E) survival of MC38gp tumor bearing animals treated with the indicated LNP. Statistics in (C) was performed by one-way ANOVA and Tukey's multiple comparison test and in (E) performed by log-rank test, **p<0.01, ***p<0.001 and ****p<0.0001.

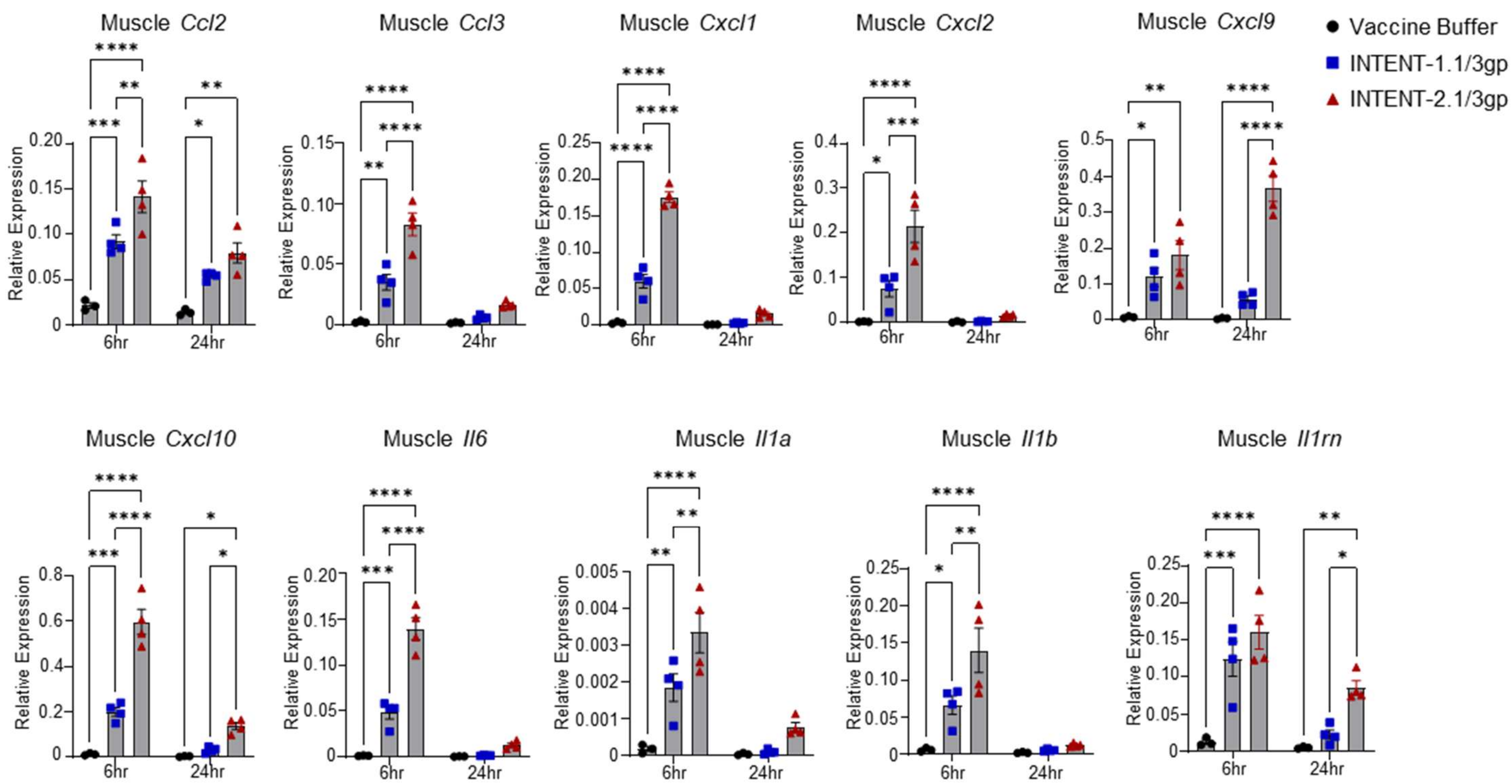

<u>Figure S5 – Adjuvant activity of INTENT LNPs in muscle tissue</u>

Expression data of the indicated genes in muscle tissue collected from the muscle tissue (injection site) following either vaccine formulation buffer, INTENT-1.1/3gp or INTENT-2.1/3gp immunization (n=3-4/group). Expression data is shown relative to *Actb*. Statistics was performed by two-way ANOVA followed by Tukey's multiple comparison test, *p<0.05, **p<0.01, ***p<0.001 and ****p<0.0001.

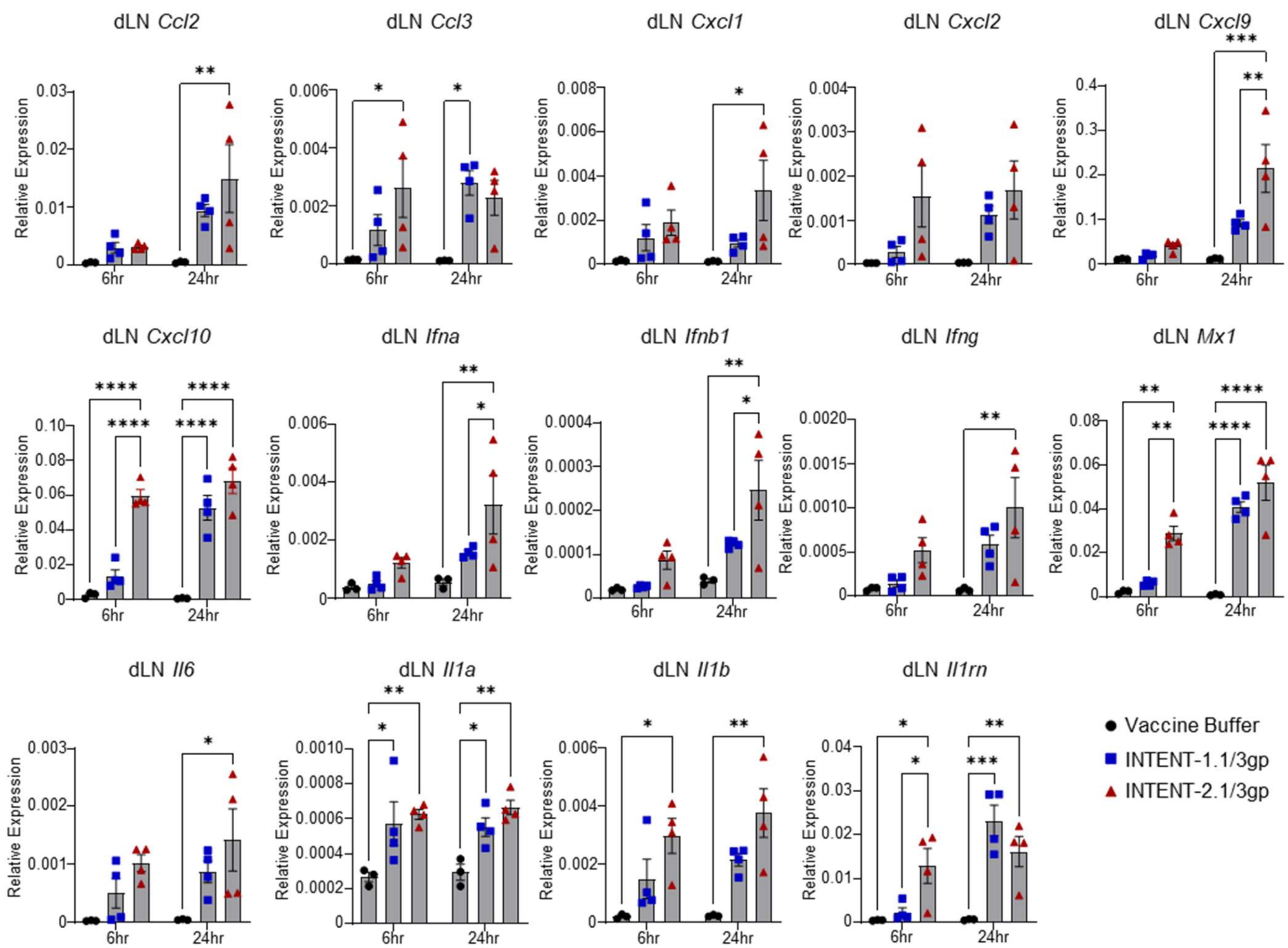

Figure S6 – Adjuvant activity of INTENT LNPs in the dLN

Expression data of the indicated genes in the draining inguinal lymph node (dLN) following either vaccine formulation buffer, INTENT-1.1/3gp or INTENT-2.1/3gp immunization (n=3-4/group). Expression data is shown relative to *Actb*. Statistics was performed by two-way ANOVA followed by Tukey's multiple comparison test, *p<0.05, **p<0.01, ***p<0.001 and ****p<0.0001.

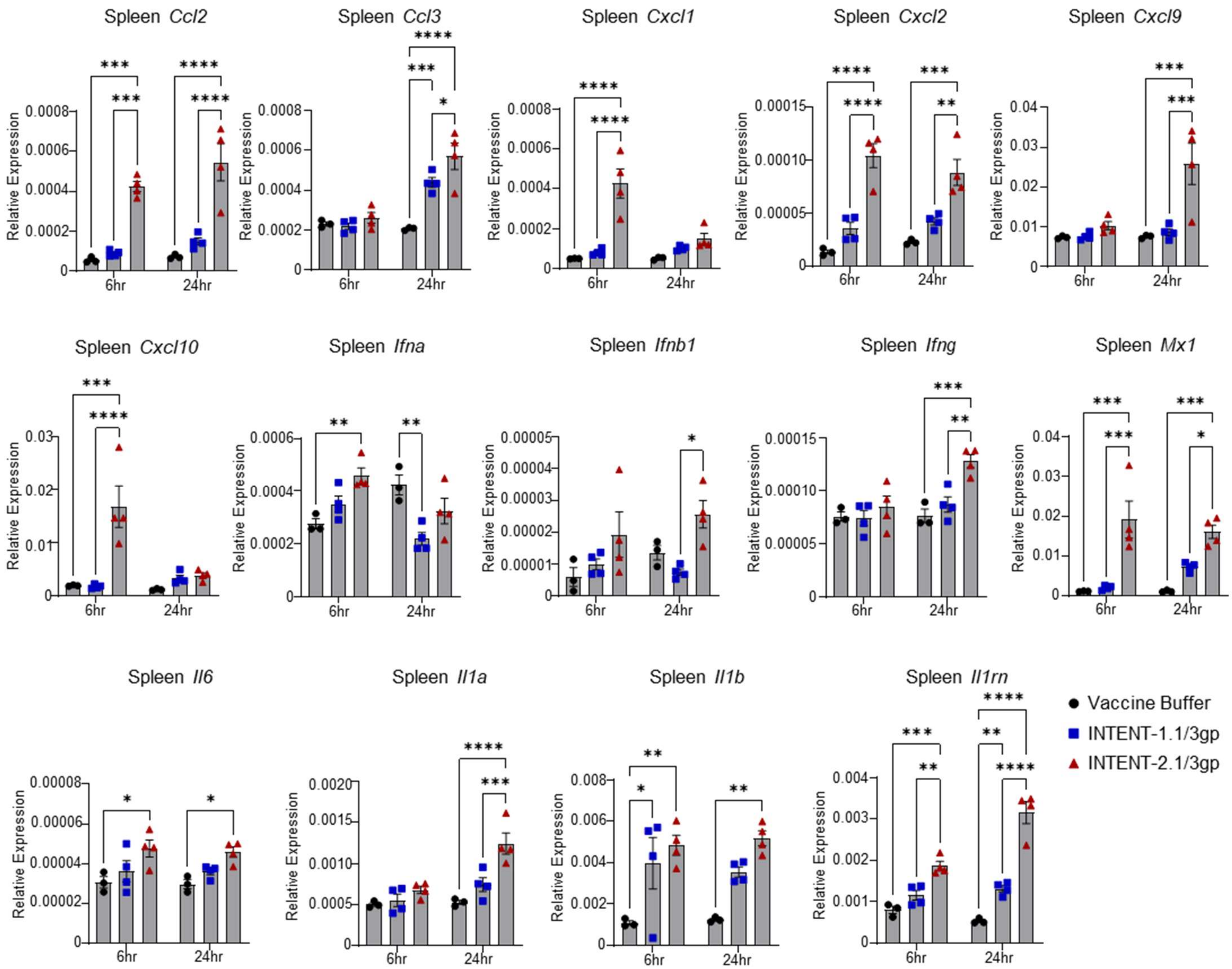

<u>Figure S7 – Adjuvant activity of INTENT LNPs in the spleen</u>

Expression data of the indicated genes in the spleen following either vaccine formulation buffer, INTENT-1.1/3gp or INTENT-2.1/3gp immunization (n=3-4/group). Expression data is shown relative to *Actb*. Statistics was performed by two-way ANOVA followed by Tukey's multiple comparison test, *p<0.05, **p<0.01, ***p<0.001 and ****p<0.0001.

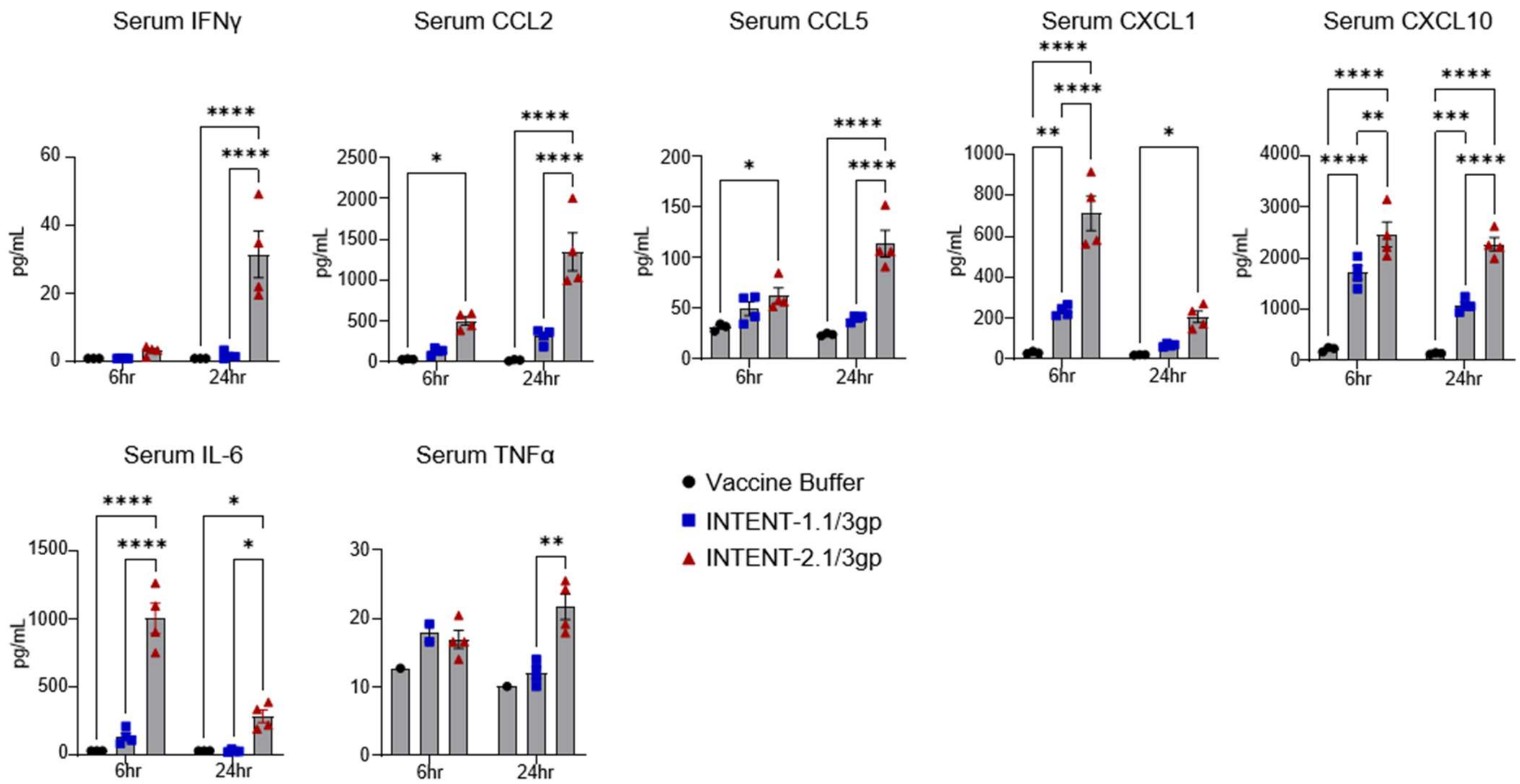

Figure S8 – Serum cytokine levels following INTENT LNP injection

Serum protein expression of the indicated cytokines/chemokines following either vaccine formulation buffer, INTENT-1.1/3gp or INTENT-2.1/3gp immunization (n=3-4/group). Statistics was performed by two-way ANOVA followed by Tukey's multiple comparison test, *p<0.05, **p<0.01, ***p<0.001 and ****p<0.0001.

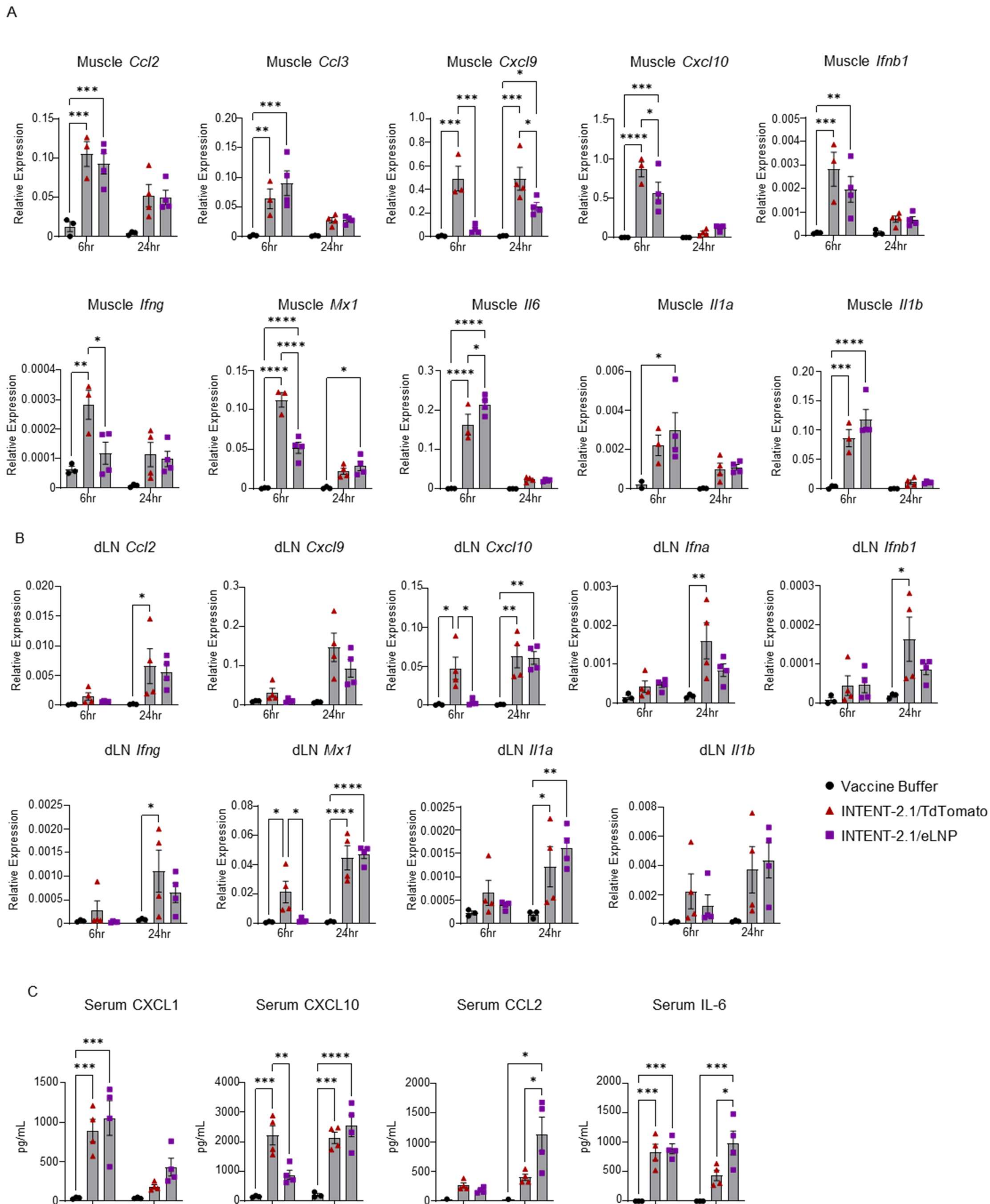
A
Muscle Ccl2
Muscle Ccl3
Muscle Cxcl9
Muscle Cxcl10
Muscle Ifnb1
Muscle Ifng
Muscle Mx1
Muscle Il6
Muscle Il1a
Muscle Il1b
B
dLN Ccl2
dLN Cxcl9
dLN Cxcl10
dLN Ifna
dLN Ifnb1
dLN Ifng
dLN Mx1
dLN Il1a
dLN Il1b
C
Serum CXCL1
Serum CXCL10
Serum CCL2
Serum IL-6
Relative Expression
pg/mL
6hr
24hr
Vaccine Buffer
INTENT-2.1/TdTomato
INTENT-2.1/eLNP

Figure S9 – INTENT-2.1/eLNP immunogenicity

Expression data of the indicated genes in the (A) muscle and (B) dLN and (C) protein levels in the serum following either vaccine formulation buffer, INTENT-2.1/3gp or INTENT-2.1/eLNP immunization (n=3-4/group). Expression data is shown relative to *Actb*. Statistics was performed by two-way ANOVA followed by Tukey's multiple comparison test, *p<0.05, **p<0.01, ***p<0.001 and ****p<0.0001.

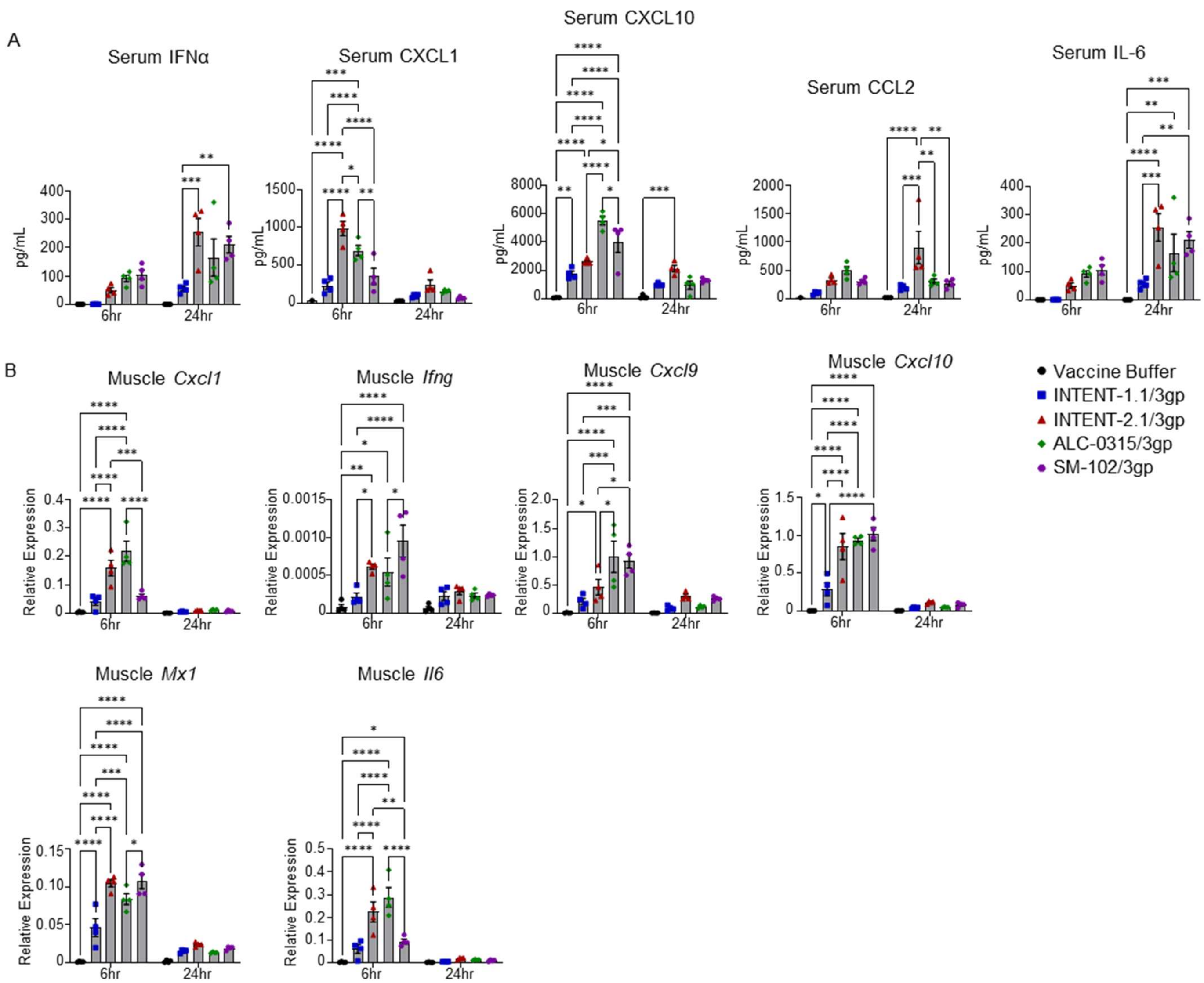

Figure S10 – Induction of cytokines and chemokines by INTENT, ALC-0315 and SM-102 LNPs in serum and muscle

C57BL/6 mice were administered vaccine buffer or the indicated LNP and (A) serum or (B) muscle tissue was collected 6 and 24 hours post injection for cytokine quantification (n=3-4). Statistics was performed by two-way ANOVA followed by Tukey's multiple comparison test, *p<0.05, **p<0.01, ***p<0.001 and ****p<0.0001.

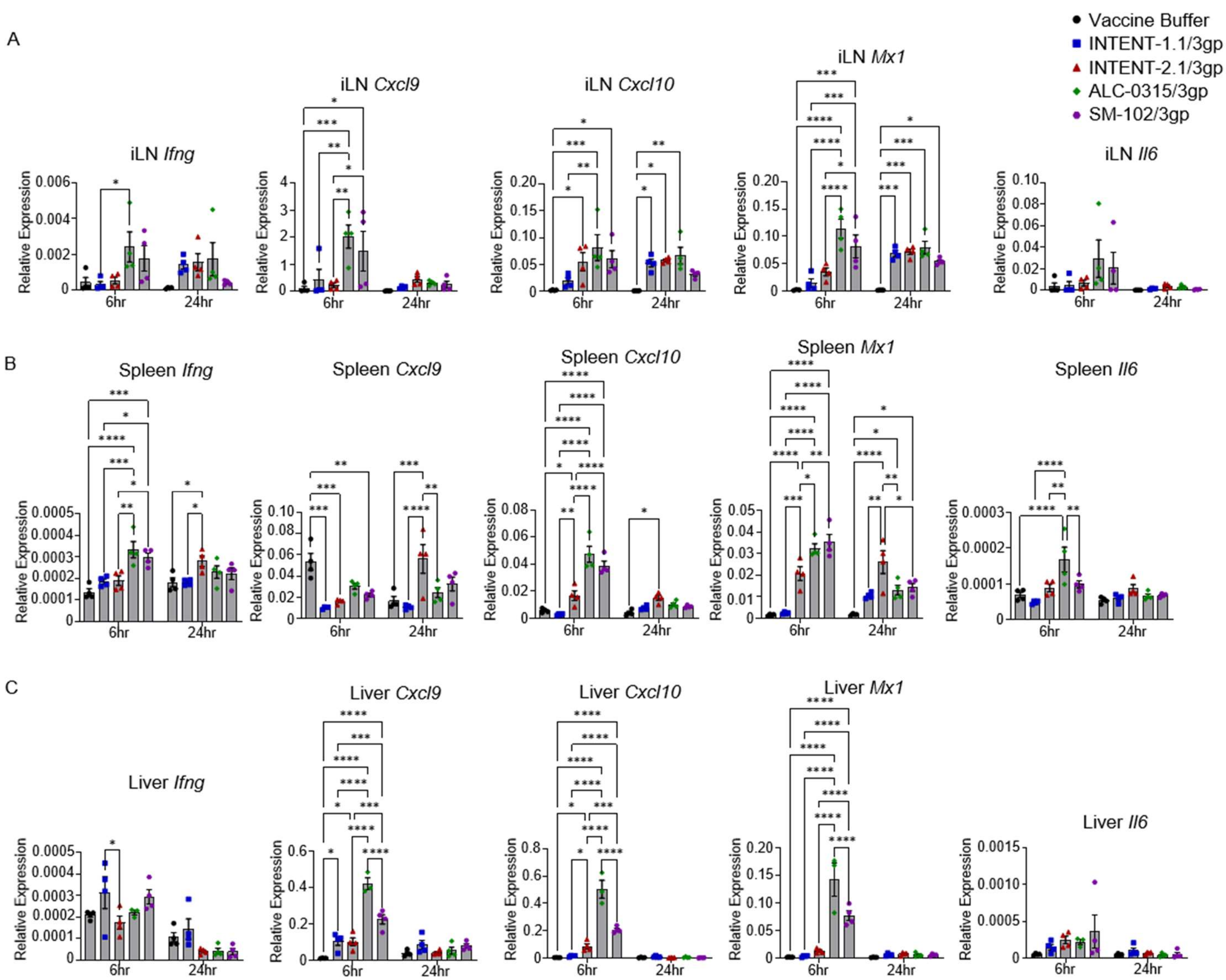

Figure S11 – Induction of cytokines and chemokines by INTENT, ALC-0315 and SM-102 LNPs in iLN, spleen and liver

C57BL/6 mice were administered vaccine buffer or the indicated LNP and (A) iLN, (B) spleen and (C) liver tissue was collected 6 and 24 hours post injection for cytokine quantification (n=3-4). Statistics was performed by two-way ANOVA followed by Tukey's multiple comparison test, *p<0.05, **p<0.01, ***p<0.001 and ****p<0.0001.

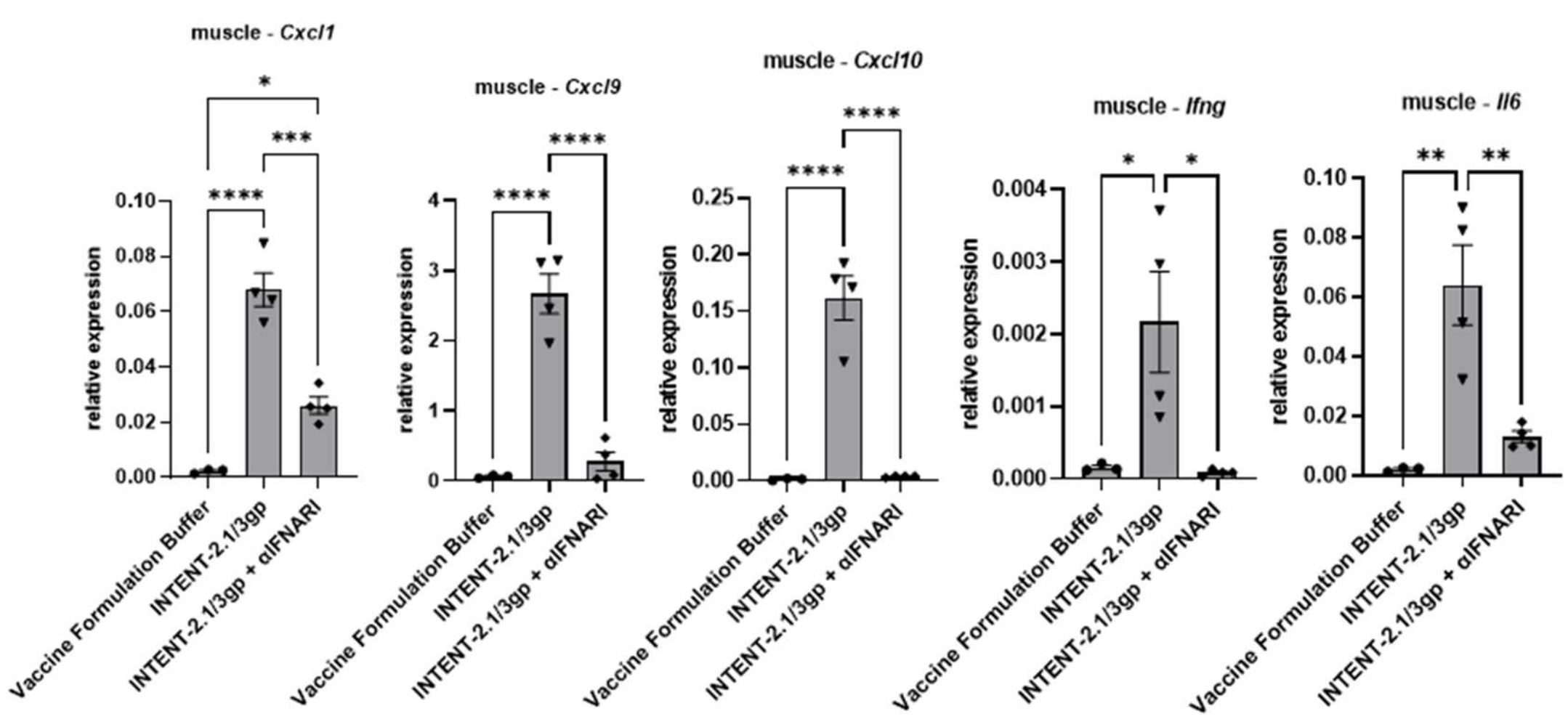

Figure S12 – INTENT-2.1/3gp immunogenicity is regulated by type I IFNs

C57BL/6 mice were administered PBS (200µl/i.p.) or αIFNARI (500µg/i.p.) prior to vaccine formulation buffer or INTENT-2.1/3gp administration (12.5µg/50µl i.m.) and expression of the indicated genes was quantified by qRT-PCR and shown relative to *Actb*. Statistics was performed by one-way ANOVA followed by Tukey's multiple comparison test, *p<0.05, **p<0.01, ***p<0.001 and ****p<0.0001.

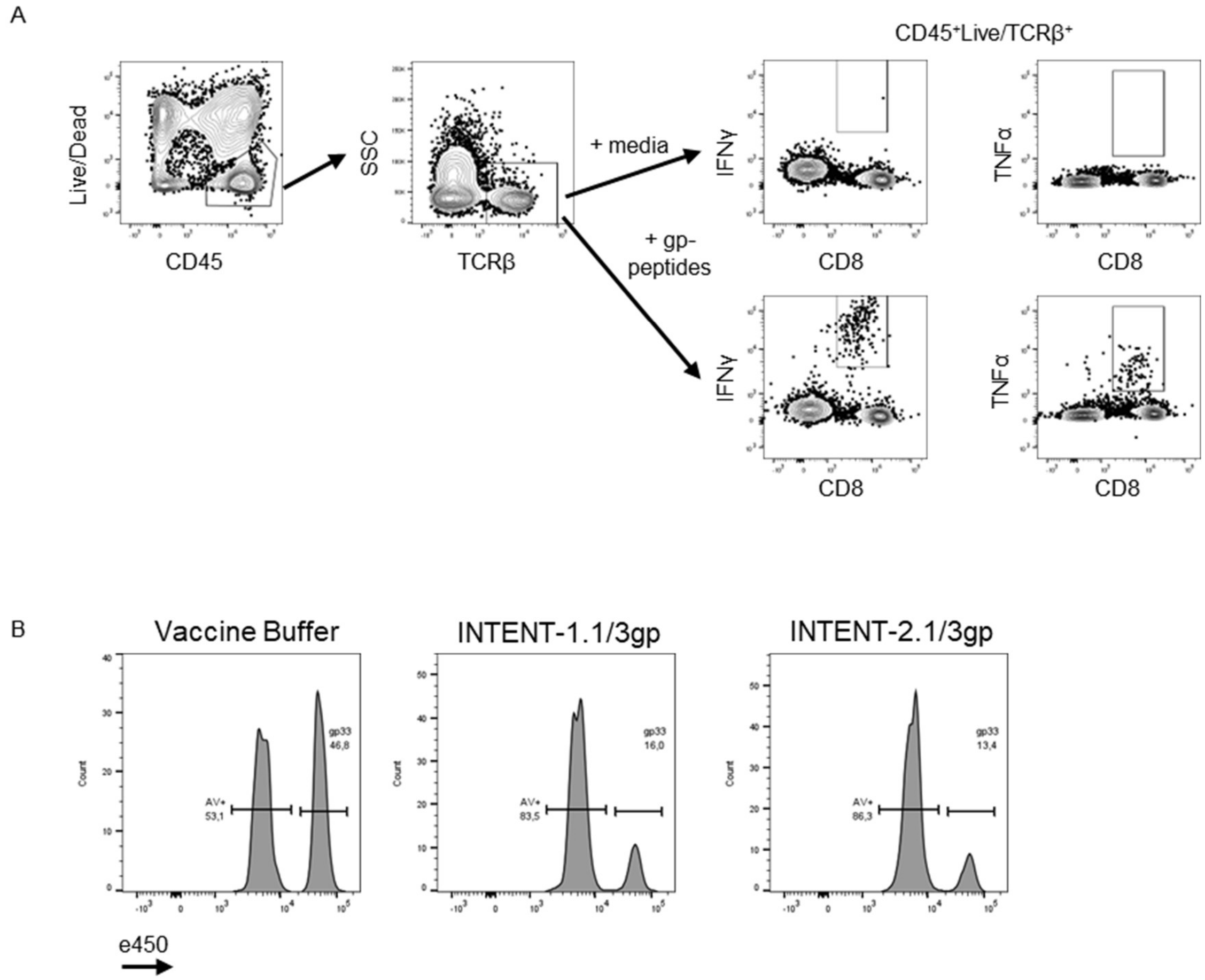

<u>Figure S13 – gp-peptide re-stimulation ICS & CTL assay</u>

(A) C57BL/6J mice were injected on days 0 and 4 with INTENT-2.1/3gp and splenocytes were collected on day 8. Cells were cultured in media alone or with the addition of gp33, gp61 and gp276 peptides (1µg/mL of each) in the presence of BrefeldinA and monensin and cultured at 37°C for 4-6 hours. Representative flow plot and gating of IFN-γ and TNF-α producing $CD8^+$ T cells. (B) Representative flow plot of splenocytes harvested from buffer or INTENT treated C57BL/6 mice following transfer of AV ($e450^{dim}$) or gp33 ($e450^{bright}$)-peptide pulsed target cells.

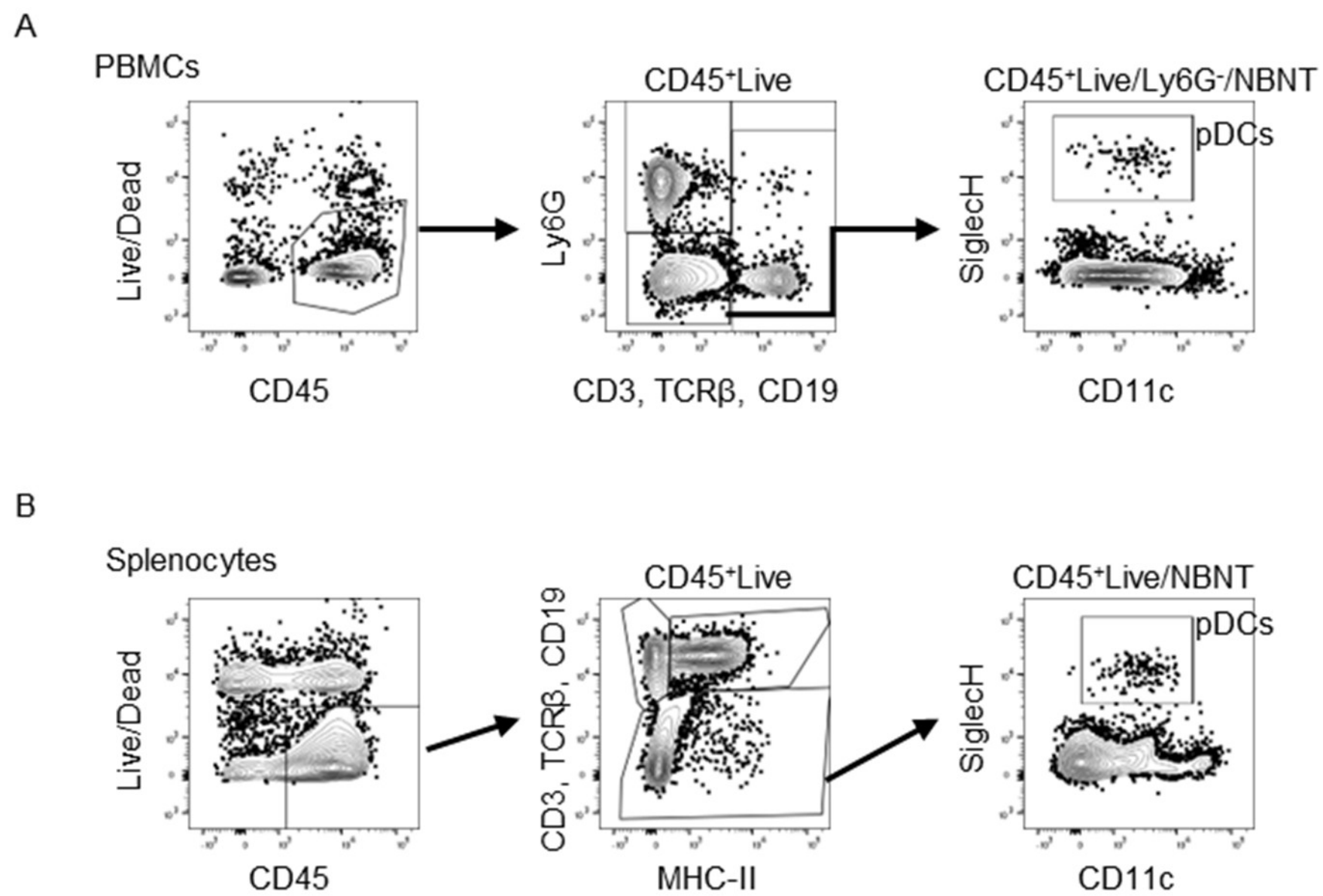

<u>Figure S14 – Representative pDC gating</u>

pDC frequency in the (A) peripheral blood and (B) splenocytes was quantified by flow cytometry and identified as SiglecH^{+}CD11c^{mid} and lacking expression of neutrophil (Ly6G) and B and T cell markers (NBNT).

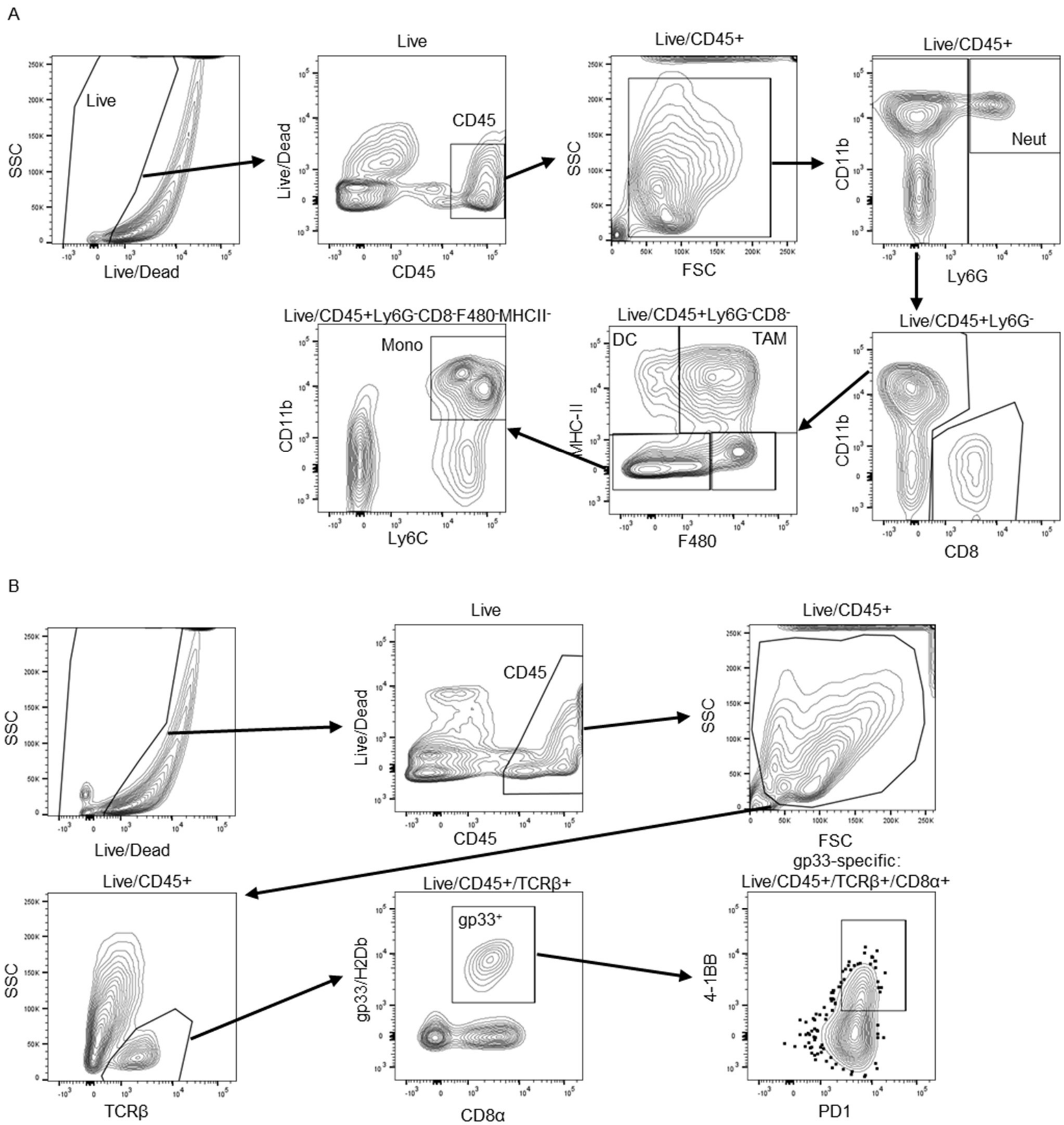

Figure S15 – Representative gating of MC38gp TME

Representative flow gating from cells isolated from MC38gp tumors with gating for (A) myeloid cells including neutrophils (Neut), tumor associated macrophages (TAM), dendritic cells (DC) and monocytes (Mono).(B) 4-1BB expression on gp33-specific $CD8^+$ tumor infiltrating lymphocytes (TIL).
